# Design, Synthesis, Biological Evaluation and Silico Prediction of Novel Sinomenine Derivatives

**DOI:** 10.3390/molecules26113466

**Published:** 2021-06-07

**Authors:** Shoujie Li, Mingjie Gao, Xin Nian, Liyu Zhang, Jinjie Li, Dongmei Cui, Chen Zhang, Changqi Zhao

**Affiliations:** 1 Gene Engineering and Biotechnology Beijing Key Laboratory, College of Life Science, Beijing Normal University, 19 XinjiekouWai Avenue, Beijing 100875, China; 201831200027@mail.bnu.edu.cn; 2 Research Group Pharmaceutical Bioinformatics, Institute of Pharmaceutical Sciences, Albert-Ludwigs-Universität Freiburg, 79106 Freiburg im Breisgau, Germany; mingjie.gao@pharmazie.uni-freiburg.de; 3 College of Pharmaceutical Science, Zhejiang University of Technology, 18 Chaowang Road, Hangzhou 310014, China; zjdqnx@126.com; 4 College of Pharmaceutical Sciences, Zhejiang University, 866 Yuhangtang Road, Hangzhou 310058, China; liyuzhang@zju.edu.cn; 5 Beijing Key Laboratory of Bioactive Substances and Functional Foods, Beijing Union University, 197 Beitucheng West Road, Beijing 100191, China; lijinjie.7785004@163.com

**Keywords:** sinomenine, synthesis, cancer, cytotoxic activity, docking study

## Abstract

Sinomenine is a morphinan alkaloid with a variety of biological activities. Its derivatives have shown significant cytotoxic activity against different cancer cell lines in many studies. In this study, two series of sinomenine derivatives were designed and synthesized by modifying the active positions C_1_ and C_4_ on the A ring of sinomenine. Twenty-three compounds were synthesized and characterized by spectroscopy (IR, ^1^H-NMR, ^13^C-NMR, and HRMS). They were further evaluated for their cytotoxic activity against five cancer cell lines, MCF-7, Hela, HepG2, SW480 and A549, and a normal cell line, Hek293, using MTT and CCK8 methods. The chlorine-containing compounds exhibited significant cytotoxic activity compared to the nucleus structure of sinomenine. Furthermore, we searched for cancer-related core targets and verified their interaction with derivatives through molecular docking. The chlorine-containing compounds **5g**, **5i**, **5j**, **6a**, **6d**, **6e**, and **6g** exhibited the best against four core targets AKT1, EGFR, HARS and KARS. The molecular docking results were consistent with the cytotoxic results. Overall, results indicate that chlorine-containing derivatives might be a promising lead for the development of new anticancer agents.

## 1. Introduction

Natural products, also known as secondary metabolites, are organic compounds with biological functions and activities synthesized by microorganisms or plants in their cells. They play an irreplaceable role and have broad application prospects in pharmaceutical, agricultural, chemical industries and other fields [1,2]. In fact, 6.1% of the total 1394 small molecule drugs developed from 1981 to 2019 were natural compounds, and 27.5% were developed by structural optimization. Furthermore, small molecule cancer drugs developed between 1940 and 2019 are directly or indirectly derived from natural products [3].

Alkaloids are nitrogen-containing organic compounds widely found in plants, animals and microorganisms. Most alkaloids have complex structures with basic nitrogen-containing heterocycles. A few alkaloids are organic amine alkaloids in which nitrogen atoms are absent in the ring structure. Thus far, around 12,000 alkaloids have been isolated from nature [4]. Based on their source or chemical structure, the alkaloids are divided into about 60 categories, including tropine, isoquinoline, indole alkaloids, terpenoids and steroidal alkaloids. Tropine alkaloids contain a tropinyl skeleton which is formed by the combination of pyrrole and piperidine rings [5]. Isoquinoline alkaloids, such as papaverine, tetrandrine, berberine, sanguinarine, sinomenine and lycorine, are derived from the phenylalanine and tyrosine pathways based on isoquinoline or tetrahydroisoquinoline [6,7,8,9,10]. Indole alkaloids, such as vinblastine and vincristine have indole skeletons and complex structures [11]. Terpenoids and steroidal alkaloids such as dendrobine, aconitine, and peiminine have a terpenoid or steroidal structure [12,13]. Alkaloids are well known for their high efficiency and low toxicity in antitumor activity.

**Figure 1:**
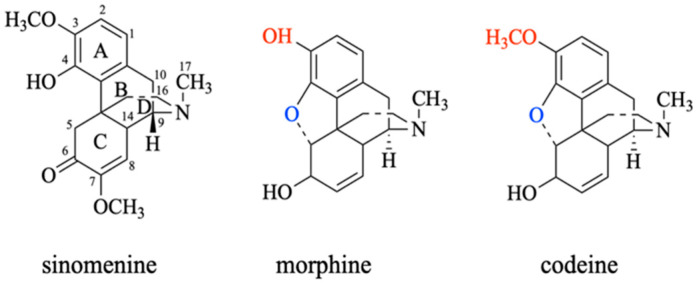
The structures of sionmenine, morphine and codeine.

The dry canes of *Sinomenium acutum* (Thunb.) Rehd. et Wils. and *Sinomenium acutum* (Thunb.) Rehd. et Wils. var. *cinereum* Rehd. et Wils., also known as Qingfeng Teng in traditional Chinese medicine, have been used in the treatment diseases [14]. In the 1920s, sinomenine was isolated from Qing Feng Teng (Japanese sabia stem) grown in Japan [15]. Several studies reported that sinomenine (an isoquinoline alkaloid monomer) carrying a phenanthrene nucleus and ethylamine bridge, has a structure is similar to the narcotics morphine and codeine. Its chemical structure is shown in Figure 1.

In previous work, structural modification of sinomenine was carried out in the active groups of its four rings [9]. The A ring is a benzene ring where C_3_ and C_4_ are substituted by methoxy and hydroxyl groups. Structural modification on the A ring is often present on the active reaction sites C_1_ and C_4_, and sometimes on C_2_ or C_3_, but it is relatively rare [16]. Different sinomenine derivatives were reported in the previous studies as having good anti-inflammatory, immunosuppressive and neuroprotective activities compared to sinomenine [17]. In this study, we designed and synthesized two new series of sinomine derivatives in a quest to obtain potent compounds.

Many scholars have reported the structural modification of the sinomenine A ring. Due to the good application effect of piperidine and tetrahydropyrrole in medicine, we took sinomenine as the leading compound and introduced piperidine methylene or tetrahydropyrrole methylene at C_1_ by Mannich reaction to obtain 1-piperidine methylated sinomenine (**5a**–**5k**) and 1-tetrahydropyrrole methylated sinomenine (**6a**–**6l**) [18]. As the phenolic hydroxyl group of C_4_ is easy to oxidize and decompose and may be a factor leading to allergic reactions in vivo, it is necessary to modify the structure to protect the phenolic hydroxyl group. Esters are important intermediates of organic synthesis. The phenol hydroxyl ester can not only increase liposolubility, enhance substance activity and change metabolic characteristics, but it can also slow down the oxidation rate of compounds and greatly reduce their allergic reactions. Therefore, a series of 1,4-bisubstituted sinomenine derivatives were synthesized by the reaction of phenylacetyl chloride with 1-tetrahydropyrrole methylated sinomenine. All structures of the products were determined by IR, ^1^H-NMR, ^13^C-NMR, and HRMS.

Furthermore, the cytotoxicity of the synthesized compounds was assessed. Compounds **5a**–**5k** were tested against three cancer cell lines, MCF-7, Hela, and HepG2, using the MTT method. Compounds **6a**–**6l** were tested against four cancer cell lines, MCF-7, Hela, SW480, and A549, and a normal cell line Hek293 with the CCK8 method. Finally, all the synthesized compounds underwent network pharmacology and molecular-docking studies in order to forecast the possible targets of the most active compounds.

## 2. Results and Discussion

### 2.1. Synthesis

**Scheme 1:**
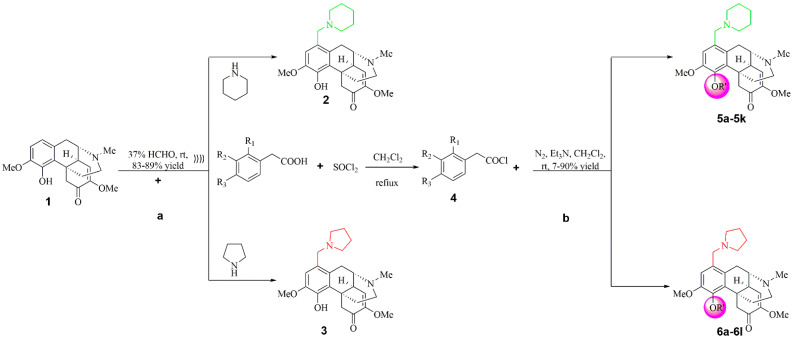
Synthetic scheme of compounds **2**–**6** (R′ = Compound **4**, the red gradient circle represents the ester substitution R.)

Two series of sinomenine derivatives were synthesized as shown in Scheme 1 and Table 1. A total of 23 new 1,4-disubstituted sinomenine derivatives (**5a**–**5k** and **6a**–**6l**) were obtained by the introduction of piperidine and tetrahydropyrrole methylene (at 1- position, step **a**) using the Mannich reaction, and then the substitution of the phenylacetyl group by an acylation reaction was performed (at 4- OH, step **b**).

### 2.2. Anticancer Activity of the Synthesized Compounds **5a**–**5k**

**Figure 2:**
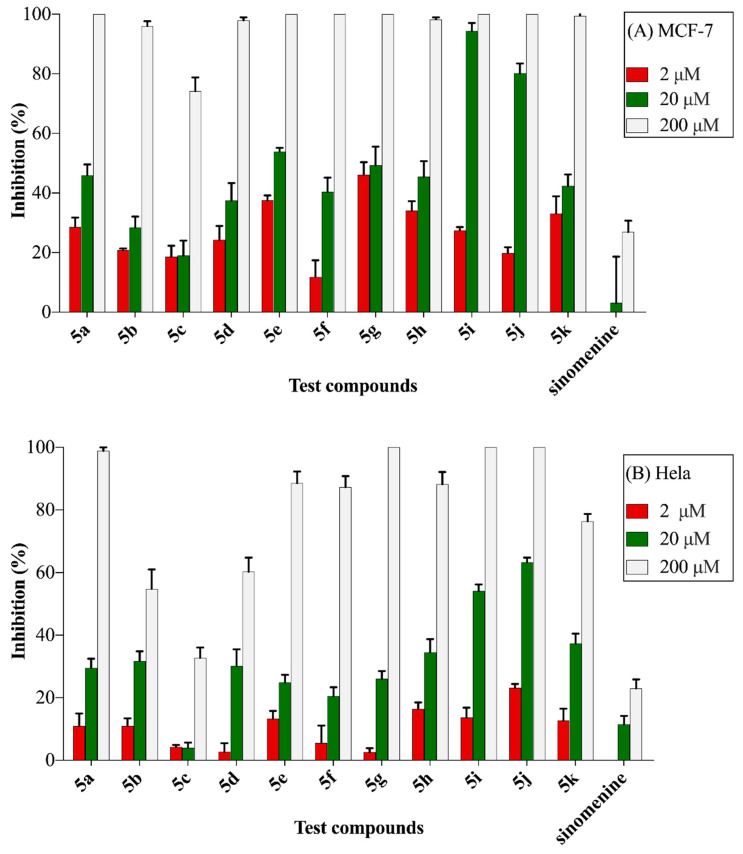

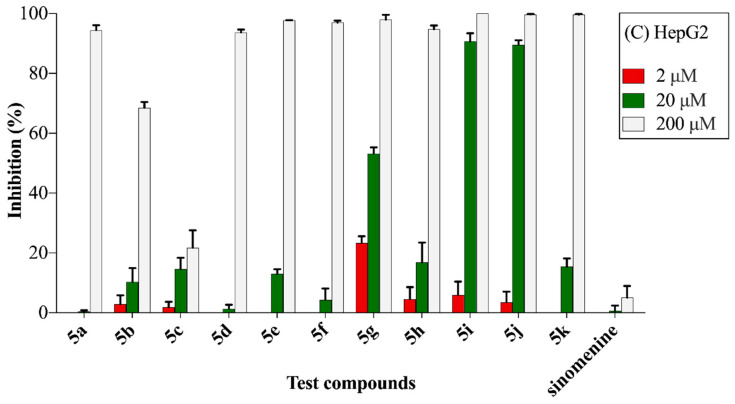
Anticancer activity of compounds **5a**–**5k** against MCF-7, Hela, and HepG2 cell lines at 2, 20, and 200 μM concentrations. (**A**) Human breast cancer cell lines, (**B**) human cervical cancer cells lines, (**C**) human hepatocellular carcinoma cell lines.

The in vitro growth inhibitory activity of the sinomenine derivatives (**5a**–**5k**) was evaluated against different three cell lines, MCF-7, Hela, and HepG2, using sinomenine as the reference compound kept under the same conditions in an MTT assay [19]. The inhibition ratios of synthetic compounds were determined at concentrations of 2, 20 and 200 μM. The obtained data in triplicate for each concentration were plotted, as shown in Figure 2. 

The results indicated that all the compounds had significant activity against the cancer cell lines. The synthesized compounds had a structure and dose-dependent relationship. At a low drug concentration of 2 μM, all of the tested compounds showed more toxicity towards the three cancer cells lines with inhibition ratios in the lower to medium percentage range for Hela and HepG2 cell lines, or higher inhibition ratios for MCF-7 cell lines. At a moderate drug concentration of 20 μM, the compounds **5i** and **5j** had significant effects on MCF-7, Hela, and HepG2 cell lines with the inhibition ratios of 54.13 and 63.35%, 94.43 and 80.22%, and 90.69 and 89.57%, respectively. Moreover, the compound **5g** showed better anticancer activity against HepG2 cell lines with inhibition ratios reaching more than 53.2%. Other tested compounds showed moderate to weak inhibition against both cancer cell lines. At a high drug concentration of 200 μM, the inhibition ratios of all synthetic compounds, except **5c**, against three cell lines exceeded 50%. In conclusion, the presence of two chlorine atoms in the R group was critical for the observed cytotoxicity activities.

### 2.3. Anticancer Activity of the Synthesized Compounds **6a**–**6l**

**Figure 3:**
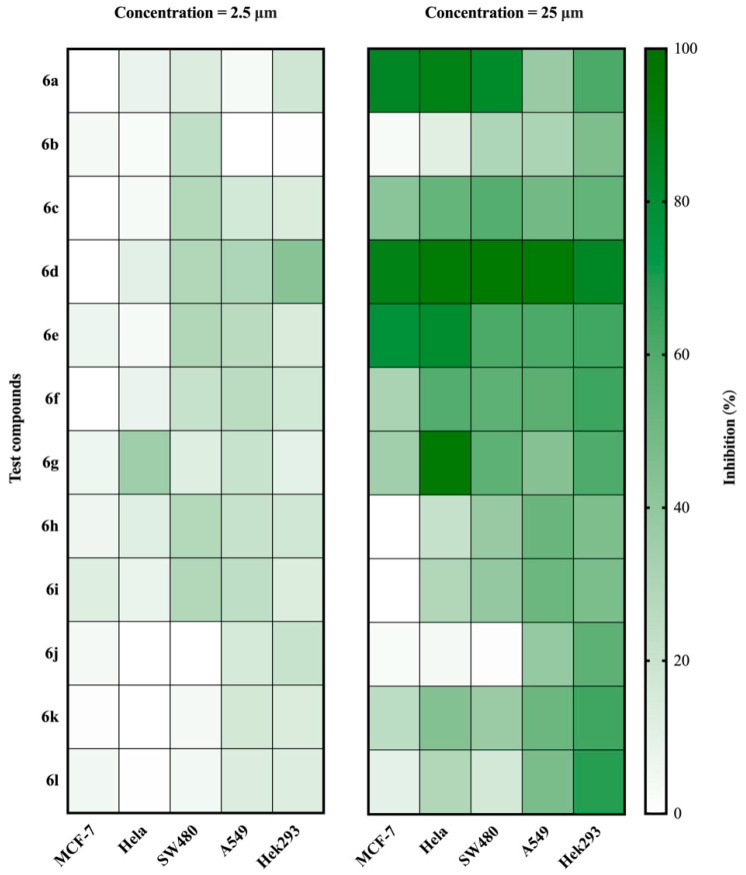
Heat map representation of the primary cytotoxic effects of novel sinomenine derivatives on different cell lines (concentration = 2.5 and 25 μM; incubation time = 60 h).

Cytotoxicity screens were performed to assess the in vitro anticancer activity of the synthesized sinomenine derivatives **6a**–**6l** on the various cancer cell lines MCF-7, Hela, SW480, and A549, as well as on Hek293 as a normal cell line, at doses of 2.5 and 25 μM. The obtained inhibition ratios data were used to construct a heat map (Figure 3, Tables S1 and S2) in order to select compounds that exert cancer cell line-specific cytotoxicity. Then, the IC_50_ values of the promising compound were determined. Cisplatin was used as a reference standard. The antiproliferative activity was based on the evaluation of the percent viability using the CCK8 assay, which is a rapid and sensitive method for determining the number of viable cells in culture. In the presence of electronic coupling reagents, WST-8 can be reduced by a dehydrogenase in the mitochondria to produce a highly water-soluble orange-yellow formazan product. Its color is proportional to the proliferation of the number of viable cells and is related to cytotoxicity. Using a microplate reader to measure the OD value can indirectly reflect the number of living cells at 450 nm wavelength [20].

Based on the heat map, **6a** and **6c**–**6g** exhibited a minimum of 50% inhibition ratios on at least one cancer cell line and had no or a very mild effect on normal cell lines. To understand structure–function relationships, these selected compounds were subjected to subsequent analyses. All six compounds (**6a** and **6c**–**6g**) were further examined to determine their IC_50_ concentrations on the previously mentioned cell lines, and their efficacy was compared to the reference drug cisplatin. For this, six compounds and cisplatin were evaluated on Hela, MCF-7, SW480, and A549, and on noncancerous Hek293 cell lines at various concentrations for 60 h. IC_50_ values were calculated (Table 2).

In agreement with the primary cytotoxicity screen, the obtained IC_50_ concentrations clearly indicated which compounds were selectively effective on one or more cancer cell lines with IC_50_ values less than 20 μM. In compounds **6a**, **6d**, **6e** and **6g**, with a chlorine atom attached to the R group, significant anticancer activities were observed compared to **6f**. The derivative **6d**, being the superior compound, displayed an IC_50_ of 5.73, 8.20, and 6.08 μM against MCF-7, Hela, and SW480, respectively. Concerning the cytotoxicity of compound **6e**, significant IC_50_ values were observed for MCF-7 and Hela cell lines (**6e**, IC_50_ = 14.86, 13.28, and 16.57 μM for MCF-7, Hela, and Hek293, respectively), indicating **6e** had more selective toxicity toward cancer cells than normal cells.

### 2.4. Computer-Aided Evaluation

#### 2.4.1. Number of Gene Screens

**Figure 4:**
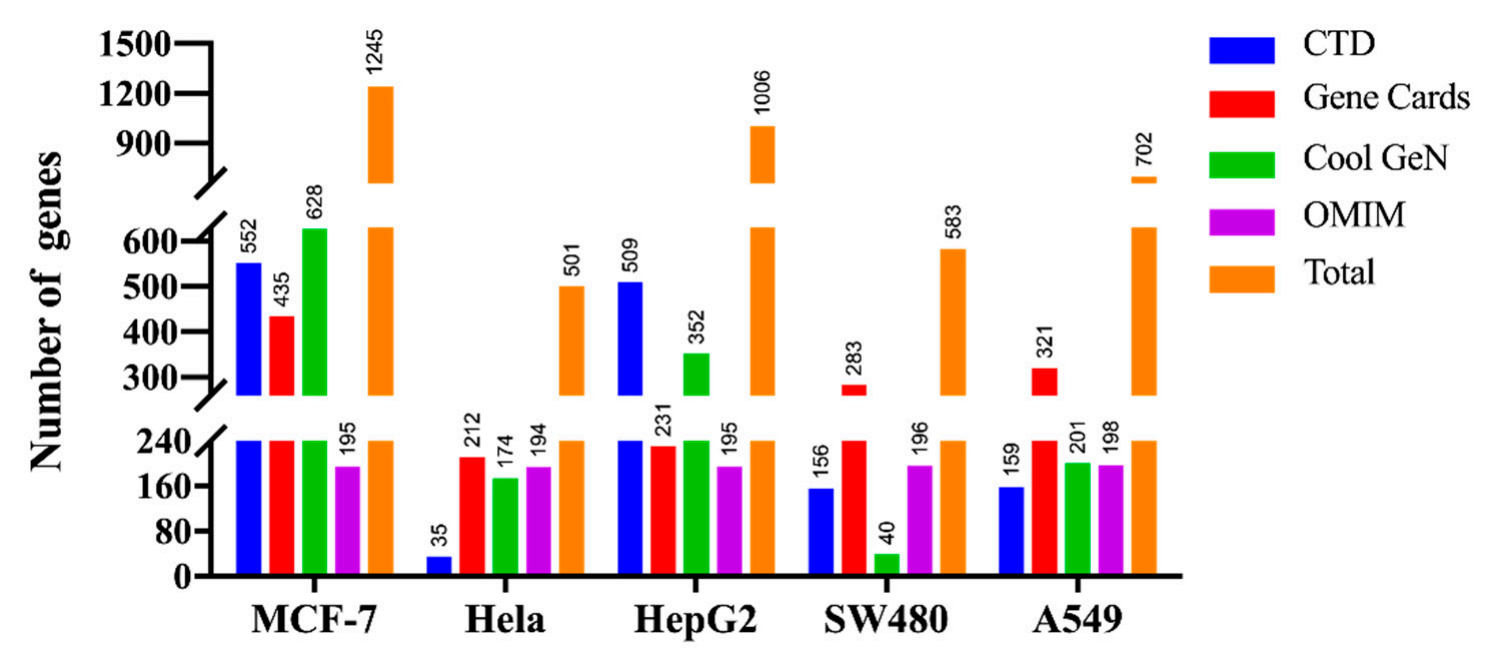
The number of genes collected in each database and the total number of genes after duplicates removed for each cancer cell.

The candidate genes of the five cell-line-related diseases viz. MCF-7 (1245 genes), Hela (501 genes), HepG2 (1006 genes), SW480 (583 genes) and A549 (702 genes), were searched, and duplicates were removed through a series of databases (Figure 4).

#### 2.4.2. PPI Interaction Network Construction

**Figure 5:**
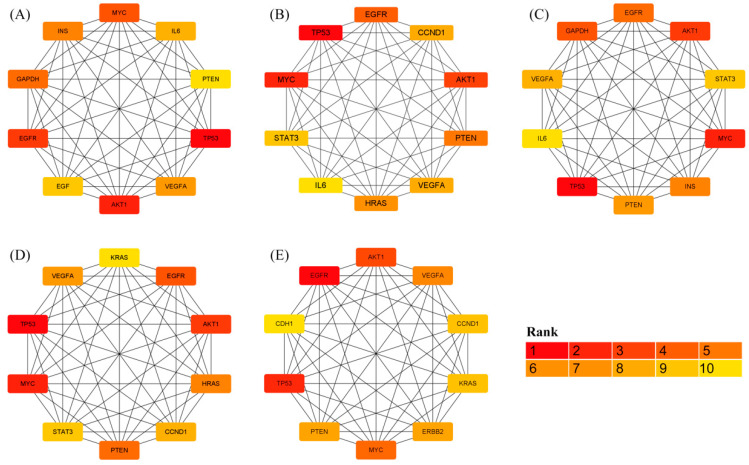
Five cancer disease-related protein interaction networks constructed by Cytoscape. Each network has 10 nodes with the top 10 degrees. (**A**) Breast cancer-related genes, (**B**) cervical cancer-related genes, (**C**) hepatocellular cancer-related genes, (**D)** colonic cancer-related genes, and (**E**) lung adenocarcinoma-related genes.

The gene was uploaded to the String database (https://string-db.org/, version 11.0b, accessed on 17 October 2020) for the analysis of PPI; the species were limited to *Homo sapiens* and the minimum interaction threshold was set to “medium confidence” 0.4. The network construction used Cytoscape software (https://cytoscape.org/, release 3.8.0, accessed on 15 April 2020). We calculated the degree of each node through the CytoHubba plug-in and selected the top 10 targets as potential targets for cancer diseases according to their associated degree. A total of 16 targets playing an important role in cancer diseases were obtained (Table 3). Subsequently, the PPI network was constructed for the top ten targets of each cancer disease and visual analysis (Figure 5). The genes AKT1, TP53, EGFR, MYC and PTEN were determined as high-frequency genes that may have a potential anticancer role.

#### 2.4.3. GO and KEGG Enrichment Analysis

**Figure 6:**
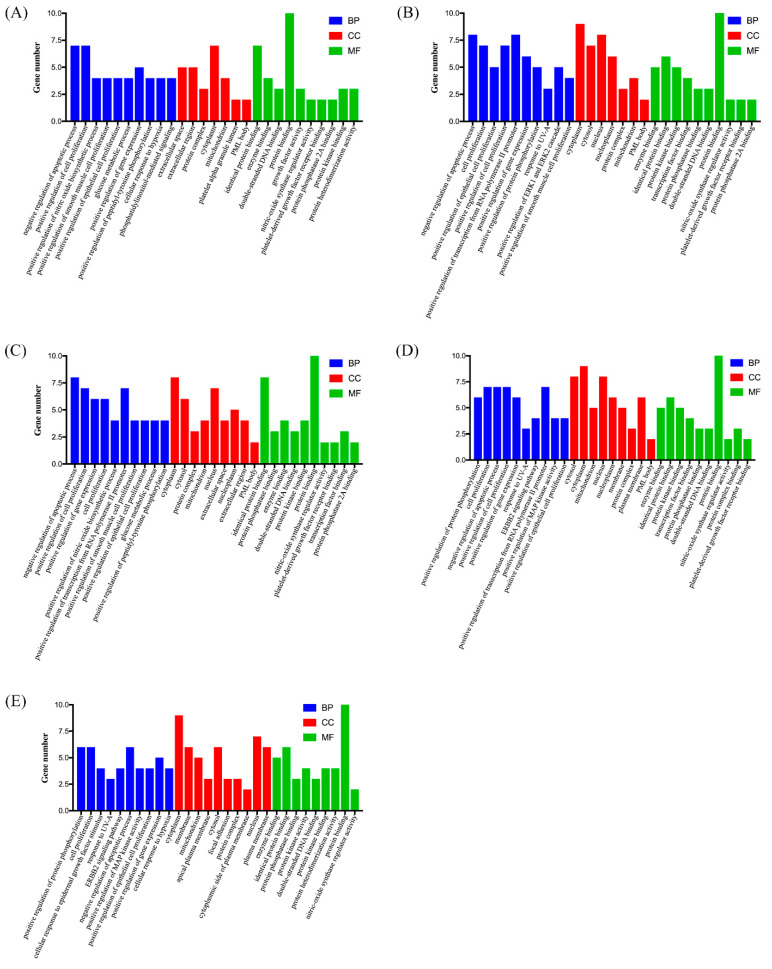
Gene ontology terms of top 10 targets of five cancer diseases (*p*-value < 0.05). BP, MF, and CC represent Biological Process, Molecular Function, and Cellular Component groups of GO, respectively. (**A**) Breast cancer, (**B**) cervical cancer, (**C**) hepatocellular cancer, (**D**) colonic cancer, and (**E**) lung adenocarcinoma.

The GO and KEGG enrichment analysis of the top 10 targets related to five cancer diseases were analyzed with the DAVID gene annotation tool. GO analysis was applied to enrich and functionally interpret differentially expressed key candidate genes (KCGs) at the molecular and cellular levels. The result of the KCGs GO was decomposed into three subontologies viz. Biological Process (BP), Cellular Component (CC) and Molecular Function (MF) as shown in Figure 6. For BP, the KCGs were mainly enriched in positive regulation of transcription from RNA polymerase II promoter, negative regulation of the apoptotic process, positive regulation of protein phosphorylation and positive regulation of cell proliferation. The KCGs in MF mainly participate in protein binding. In addition, most of the KCGs were localized to regions called the cytoplasm, plasma membrane, cytosol and the nucleus of the CC. 

**Figure 7:**
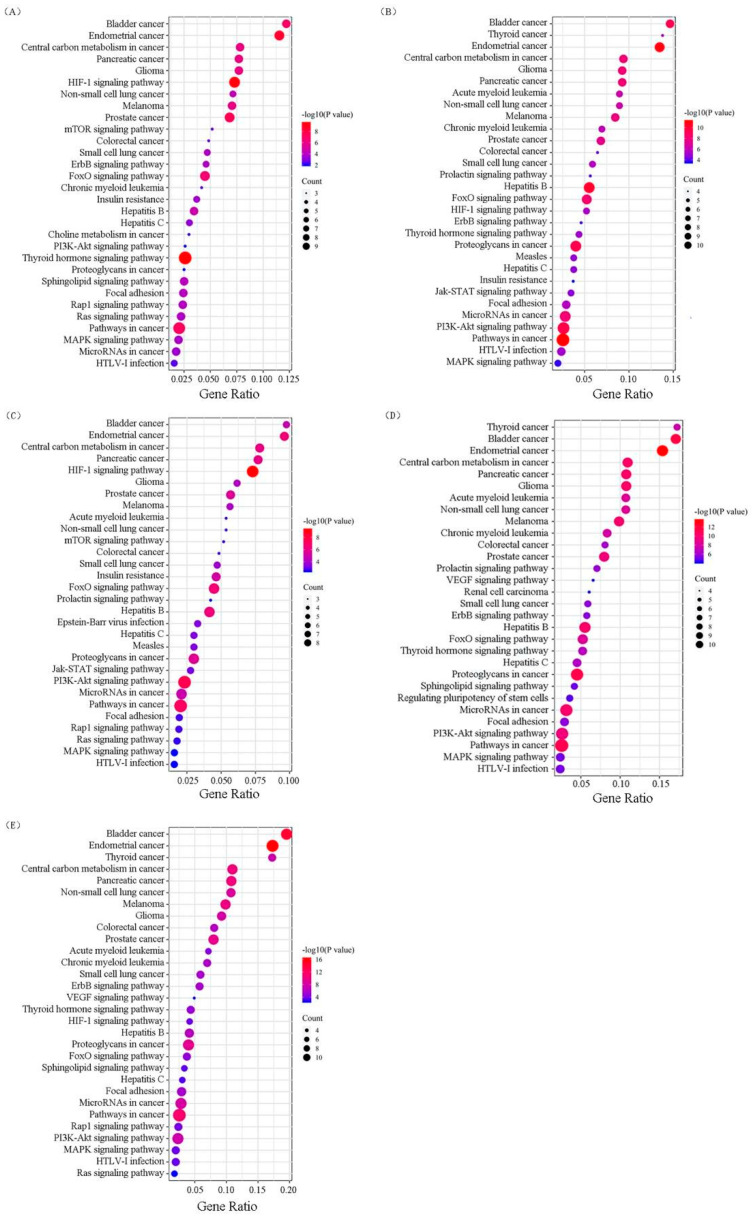
Bubble plot of KEGG pathway enrichment analysis of the genes related to the five cancer diseases. Bubble plot: letters on the left are KEGG names, numbers on the bottom are the proportions of genes, sizes of the circles indicate the numbers of enriched genes, and colors reflect *p*-values. The redder the colors are, the more enriched the genes, and the smaller the *p* values. (*p* is normalized according to −log10). (**A**) Breast cancer, (**B**) cervical cancer, (**C**) hepatocellular cancer, (**D**) colonic cancer and (**E**) lung adenocarcinoma.

KEGG pathway databases contain vital information for systematic pathway enrichment analysis of gene functions. Significantly enriched pathways as therapeutic targets in cancer, including the PI3K/Akt signaling pathway, MAPK signaling pathway, ErbB signaling pathway, p53 signaling pathway and FoxO signaling pathway, were identified. The top 30 integrated KEGG pathways of each disease are shown in Figure 7.

#### 2.4.4. Molecular Docking

**Figure 8:**
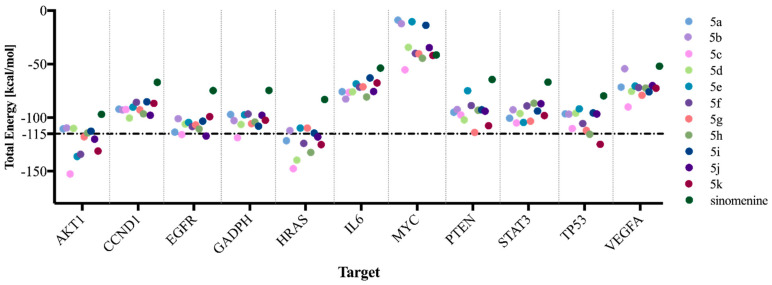
The total energy of molecular docking between compounds **5a**–**5k** and 11 potential targets.

**Figure 9:**
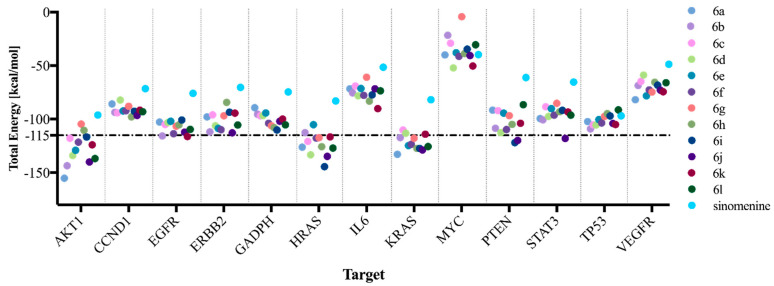
The total energy of molecular docking between compounds **6a**–**6l** and 13 potential targets.

Molecular docking was carried out to elucidate the binding modes of the 24 compounds to the 16 targets (AKT1, CCND1, CDH1, EGF, EGFR, ERBB2, GAPDH, HRAS, IL6, INS, KRAS, MYC, PTEN, STAT3, TP53 and VEGFA) for which crystal structures were known. Interestingly, most of the compounds showed a relatively much higher binding affinity against targets such as AKT1 (PDB ID: 4EJN), KRAS (PDB ID: 4LYH), HRAS (PDB ID: 121P) and EGFR (PDB ID: 1M17) than the other 10 targets (Figure 8 and Figure 9). For example, **6a** interacted with AKT1 with a docking score of −155.153 kcal/mol, and **6d** interacted with HRAS with a docking score of −133.267, which should be deemed as potent binding. The docking scores of the best-ranked molecules against the selected targets are shown in Tables S3 and S4 and Figure S59.

**Figure 10:**
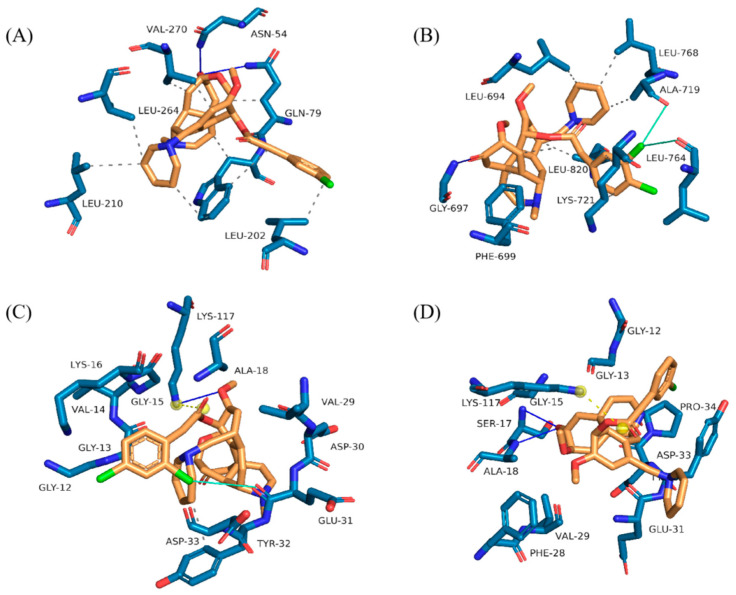
The binding pose of the selected ligands. (**A**) **5g** docked into the binding cavity of the protein AKT1; (**B**) **5j** docked into the binding cavity of the protein EGFR; (**C**) **5i** docked into the binding cavity of the protein HRAS; (**D**) **5g** docked into the binding cavity of the protein HRAS.

**Figure 11:**
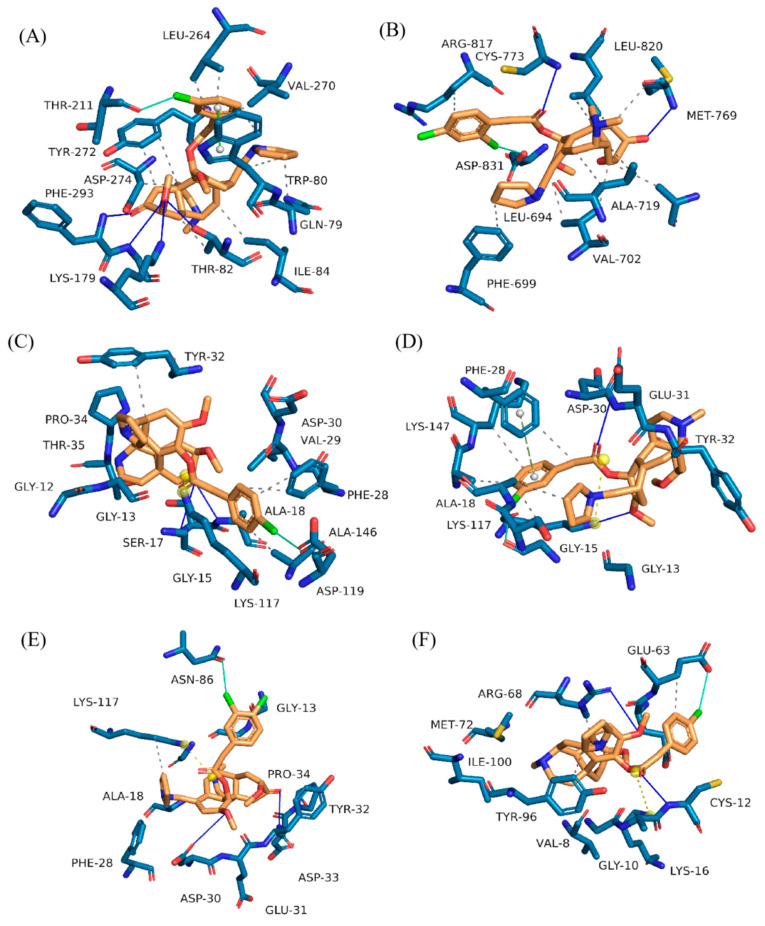
The binding pose of the selected ligands. (**A**) **6e** docked into the binding cavity of the protein AKT1; (**B**) **6d** docked into the binding cavity of the protein EGFR; (**C**) **6a** docked into the binding cavity of the protein HRAS; (**D**) **6e** docked into the binding cavity of the protein HRAS; (**E**) **6g** docked into the binding cavity of the protein HRAS; (**F**) **6a** docked into the binding cavity of the protein KRAS. The ligand is represented by orange sticks. The active site residues are shown as blue sticks. The main atoms involving hydrogen bonds are indicated by blue lines. The main atoms involving hydrophobic bonds are indicated by grey dashes. The main atoms involving halogen bonds are indicated by green lines. The main atoms involving salt bridges are indicated by yellow dotted lines. The main atoms involving π-stacking are indicated by green dotted lines. The key residues participating in hydrogen bonds and hydrophobic interactions are labeled.

As shown in Figure 10 and Figure 11 and Tables S5–S8, a stable composite structure was formed between the compound and the protein through hydrophobic interaction, hydrogen bonding, halogen bonding, salt bridges and π stacking, thereby affecting the structure and function of the protein and causing cancer cell death. For example, **6e** bound to the cavity of 121P (HRAS) and interacted with eight amino acid residues. The interaction between **6e** and six amino acid residues (ALA18, PHE28, TYR32, LYS117, ALA146 and LYS147) was hydrophobic. Moreover, **6e** interacted with ASP30 and LYS117 through hydrogen bonds, with GLY15A through halogen bonds, with LYS117 through salt bridges and with PHE28 through π stacking interaction.

## 3. Materials and Methods

### 3.1. Chemistry

Unless specified otherwise, all the materials were obtained from commercial suppliers and used without further purification. Thin layer chromatography (TLC) was performed using silica gel 60 F_254_ and visualized using UV light. Column chromatography was performed with silica gel (mesh 300–400). ^1^H NMR and ^13^C NMR spectra were recorded on a Bruker Avance 500 MHz spectrometer in CDCl_3_ with Me_4_Si as an internal standard. Data were reported as follows: chemical shift in ppm (*δ*), multiplicity (*s* = singlet, *d* = doublet, *t* = triplet, *q* = quartet, *br* = broad and *m* = multiplet), coupling constant in Herts (Hz), and integration. IR spectra were recorded on an FT-IR spectrometer, and only major peaks are reported in cm^−1^. Mass data were recorded by ESI on an FT mass spectrometer.

#### 3.1.1. General Procedure for the Synthesis of Compounds **5a**–**5k**

To a mixture of 1-(1-pyrrolidinylmethyl)-Sinomenine or 1-(1-piperidinylmethyl)-Sinomenine (1.0 mmol) and Et_3_N (2.5 mmol) in CH_2_Cl_2_ (5 mL) was added acyl chloride (2.5 mmol) at 0 °C. The reaction mixture was then stirred at room temperature. After the completion of the reaction, sat. NaHCO_3_ was added and then extracted with CH_2_Cl_2_. The organic layers were combined and dried with anhydrous Na_2_SO_4_ and evaporated under reduced pressure. The mixture was evaporated under vacuum, and the residue was purified by flash chromatography with dichloromethane and methanol as the eluents to produce the pure product.

(4bR,8aS,9S)-3,7-dimethoxy-11-methyl-6-oxo-1-(piperidin-1-ylmethyl)-6,8a,9,10-tetrahydro-5H-9,4b-(epiminoethano)phenanthren-4-yl 2-(4-chlorophenyl)acetate (**5a**)

White solid; Yield: 493.0 mg, 90%; m.p.:105–107 °C; IR (KBr, cm^−1^): 3426, 2931, 1761, 1693, 1633, 1469, 1384, 1120, 1016, 867; ^1^H NMR (500 MHz, CDCl3): δ 7.37 (d, *J* = 8.0 Hz, 2H), 7.32 (d, *J* = 8.3 Hz, 2H), 6.70 (s, 1H), 5.45 (s, 1H), 3.92 (d, *J* = 15.1 Hz, 1H), 3.82 (d, *J* = 15.1 Hz, 1H), 3.66 (d, *J* = 15.9 Hz, 1H), 3.59 (s, 3H), 3.44 (s, 3H), 3.21–3.39 (m, 3H), 3.19 (s, 1H), 2.91 (s, 1H), 2.62 (d, *J* = 16.3 Hz, 1H), 2.42–2.47 (m, 1H), 2.40 (s, 3H), 2.36 (d, *J* = 16.0 Hz, 1H), 2.21–2.39 (m, 4H), 1.98 (t, *J* = 11.0 Hz, 1H), 1.74 (t, *J* = 10.2 Hz, 1H), 1.44–1.53 (m, 5H), 1.35–1.43 (m, 2H) (Figure S1); ^13^C NMR (125 MHz, CDCl3): δ 192.2, 168.9, 152.4, 148.8, 138.5, 133.4, 133.1, 131.8, 131.1, 129.8, 129.5, 128.6, 115.2, 113.3, 61.9, 56.2, 55.8, 54.7, 54.4, 50.1, 46.6, 45.7, 42.7, 40.8, 40.6, 36.9, 26.2, 24.4, 20.8 (Figure S2). 

(4bR,8aS,9S)-3,7-dimethoxy-11-methyl-6-oxo-1-(piperidin-1-ylmethyl)-6,8a,9,10-tetrahydro-5H-9,4b-(epiminoethano)phenanthren-4-yl 2-phenylacetate (**5b**)

White solid; Yield: 513.5 mg, 64%; m.p.:192–194 °C; IR (KBr, cm^−1^): 3413, 2917, 1749, 1686, 1632, 1471, 1382, 1134, 1030, 700; ^1^H NMR (500 MHz, CDCl3): δ 7.44 (d, *J* = 7.4 Hz, 2H), 7.36 (t, *J* = 7.6, 2H), 7.24 -7.31 (m, 1H), 6.71 (s, 1H), 5.45 (d, *J* = 1.9 Hz, 1H), 3.96 (d, J = 14.9 Hz, 1H), 3.88 (d, *J* = 14.9 Hz, 1H,), 3.68 (d, *J* = 16.2 Hz, 1H), 3.61 (s, 3H), 3.45 (s, 3H), 3.23–3.39 (m, 3H), 3.17–3.22 (m, 1H), 2.91 (s, 1H), 2.64 (d, *J* = 14.9 Hz, 1H), 2.43–2.47 (m, 1H), 2.41 (s, 3H), 2.22–2.37 (m, 5H), 1.91–2.07 (m, 1H), 1.65–1.77 (m, 1H), 1.45–1.56 (m, 5H), 1.42 (d, *J* = 4.6 Hz, 2H) (Figure S3); ^13^C NMR (125 MHz, CDCl3): δ 192.3, 169.4, 152.4, 149.0, 138.7, 133.4, 133.3, 129.9, 129.7, 129.5, 128.5, 127.1, 115.2, 113.4, 62.0, 56.3, 55.8, 54.8, 54.5, 50.0, 46.7, 45.7, 42.7, 41.5, 40.6, 36.9, 26.3, 24.5, 20.8 (Figure S4). 

(4bR,8aS,9S)-3,7-dimethoxy-11-methyl-6-oxo-1-(piperidin-1-ylmethyl)-6,8a,9,10-tetrahydro-5H-9,4b-(epiminoethano)phenanthren-4-yl 2-(3,4-dimethoxyphenyl)acetate (**5c**)

White solid; Yield: 654.6 mg, 90%; m.p.:151–154 °C; IR (KBr, cm^−1^): 3415, 2935, 1762, 1690, 1618, 1466, 1384, 1106, 863; ^1^H NMR (500 MHz, CDCl3): δ 6.99–7.05 (m, 1H), 6.97 (dd, *J* = 8.2, 1.8 Hz, 1H), 6.87 (d, *J* = 8.2 Hz, 1H), 6.73 (s, 1H), 5.44 (d, *J* = 1.8 Hz, 1H), 3.94 (s, 3H), 3.79–3.91 (m, 5H), 3.65 (s, 3H), 3.58 (d, *J* = 15.5 Hz, 1H), 3.46 (s, 3H), 3.23–3.42 (m, 3H), 3.17–3.22 (m, 1H), 2.89 (s, 1H), 2.64 (d, *J* = 16.3 Hz, 1H), 2.43–2.50 (m, 1H), 2.42 (s, 3H), 2.26–2.35 (m, 4H), 2.23 (d, *J* = 16.1 Hz, 1H), 2.00 (t, *J* = 13.8 Hz, 1H), 1.66 (t, *J* = 10.3 Hz, 1H), 1.38–1.56 (m,7H) (Figure S5); ^13^C NMR (125 MHz, CDCl3): δ 192.4, 169.5, 152.5, 149.2, 149.0, 148.4, 138.8, 133.4, 130.0, 129.6, 125.8, 122.0, 115.2, 113.5, 113.3, 111.6, 62.1, 56.3, 56.0, 55.96, 54.8, 54.6, 49.8, 46.8, 45.7, 42.8, 41.2, 40.6, 36.9, 26.3, 24.5, 20.9 (Figure S6).

(4bR,8aS,9S)-3,7-dimethoxy-11-methyl-6-oxo-1-(piperidin-1-ylmethyl)-6,8a,9,10-tetrahydro-5H-9,4b-(epiminoethano)phenanthren-4-yl 2-(o-tolyl)acetate (**5d**)

White solid; Yield: 472.7 mg, 85%; m.p.:177–178 °C; IR (KBr, cm^−1^): 3417, 2938, 1745, 1686, 1633, 1472, 1384, 1198, 1145, 1107, 1068, 751; ^1^H NMR (500 MHz, CDCl3): δ 7.29–7.40 (m, 1H), 7.16–7.24 (m, 3H), 6.71 (s, 1H), 5.44 (d, *J* = 1.4 Hz, 1H), 3.97 (d, *J* = 15.4 Hz, 1H), 3.91 (d, *J* = 15.5 Hz, 1H), 3.56–3.66 (m, 4H), 3.45 (s, 3H), 3.22–3.39 (m, 3H), 3.20 (s, 1H), 2.91 (s, 1H), 2.56–2.71 (m, 1H), 2.43–2.55 (m, 4H), 2.42 (s, 3H), 2.23–2.36 (m, 5H), 1.92–2.07 (m, 1H), 1.66–1.80 (m, 1H), 1.46–1.55 (m, 5H), 1.36–1.45 (m, 2H) (Figure S7); ^13^C NMR (125 MHz, CDCl3): δ 192.2, 169.3, 152.4, 149.0, 138.7, 137.3, 133.3, 132.0, 130.6, 130.4, 130.0, 129.5, 127.4, 126.1, 115.1, 113.4, 62.0, 56.3, 55.8, 54.7, 54.5, 49.8, 46.7, 45.7, 42.7, 40.6, 39.2, 36.9, 26.2, 24.5, 20.9, 19.6 (Figure S8). 

(4bR,8aS,9S)-3,7-dimethoxy-11-methyl-6-oxo-1-(piperidin-1-ylmethyl)-6,8a,9,10-tetrahydro-5H-9,4b-(epiminoethano)phenanthren-4-yl 3-phenylpropanoate (**5e**)

White solid; Yield: 306.3 mg, 55%; m.p.:181–185 °C; IR (KBr, cm^−1^): 3459, 2931, 1760, 1692, 1629, 1469, 1384, 1203, 1126, 987; ^1^H NMR (500 MHz, CDCl3): δ 7.26–7.29 (m, 4H), 7.16–7.22 (m, 1H), 6.72 (s, 1H), 5.46 (d, *J* = 1.5 Hz, 1H), 3.76 (d, *J* = 16.0 Hz, 1H), 3.63 (s, 3H), 3.44 (s, 3H), 3.22–3.37 (m, 3H), 3.18–3.21 (m, 1H), 3.03–3.09 (m, 2H), 3.00 (t, *J* = 7.0 Hz, 1H), 2.87–2.94 (m, 2H), 2.63 (d, *J* = 15.2 Hz, 1H), 2.41 (s, 3H), 2.34–2.40 (m, 2H), 2.23–2.35 (m, 4H), 1.90–2.05 (m, 1H), 1.59–1.75 (m, 1H), 1.45–1.58 (m, 5H), 1.38–1.44 (m, 2H) (Figure S9); ^13^C NMR (125 MHz, CDCl_3_): δ = 192.2, 170.7, 152.4, 148.9, 140.3, 138.5, 133.2, 129.8, 129.4, 128.5, 128.3, 126.1, 115.3, 113.2, 62.0, 56.2, 55.7, 54.7, 54.4, 50.2, 46.6, 45.7, 42.6, 40.6, 36.8, 35.7, 30.5, 26.2, 24.4, 20.8 (Figure S10).

(4bR,8aS,9S)-3,7-dimethoxy-11-methyl-6-oxo-1-(piperidin-1-ylmethyl)-6,8a,9,10-tetrahydro-5H-9,4b-(epiminoethano)phenanthren-4-yl 2-(2-chlorophenyl)acetate (**5f**)

White solid; Yield: 442.8 mg, 77%; m.p.:188–190 °C; IR (KBr, cm^−1^): 3414, 2926, 1749, 1684, 1633, 1472, 1384, 1225, 1145, 1107, 1031, 762; ^1^H NMR (500 MHz, CDCl3): δ 7.46 (s, 1H), 7.40 (dd, *J* = 7.8, 1.4 Hz, 1H), 7.19–7.30 (m, 2H), 6.72 (s, 1H), 5.46 (d, *J* = 1.9 Hz, 1H), 4.12 (d, *J* = 16.5 Hz, 1H), 4.05 (d, *J* = 16.5 Hz, 1H), 3.73–3.79 (m, 1H), 3.68 (s, 3H), 3.45 (s, 3H), 3.22–3.40 (m, 3H), 3.17–3.22 (m, 1H), 2.94 (s, 1H), 2.62 (dd, *J* = 18.2, 3.9 Hz, 1H), 2.43–2.49 (m, 1H), 2.36–2.42 (m, 4H), 2.20–2.35 (m, 4H), 1.80 (t, *J* = 12.5, 1H), 1.62 (d, *J* = 12.4 Hz, 1H), 1.45–1.57 (m, 5H), 1.35–1.44 (m, 2H) (Figure S11); ^13^C NMR (125 MHz, CDCl3): δ 192.3, 168.3, 152.4, 148.9, 138.7, 134.5, 133.4, 132.1, 131.8, 129.9, 129.5, 129.4, 128.8, 127.0, 115.3, 113.4, 62.0, 56.3, 55.9, 54.7, 54.5, 50.0, 46.7, 45.7, 42.7, 40.7, 39.1, 37.0, 26.2, 24.5, 20.9 (Figure S12). 

(4bR,8aS,9S)-3,7-dimethoxy-11-methyl-6-oxo-1-(piperidin-1-ylmethyl)-6,8a,9,10-tetrahydro-5H-9,4b-(epiminoethano)phenanthren-4-yl 2-(3-chlorophenyl)acetate (**5g**)

White solid; Yield: 418.1 mg, 72%; m.p.:164–167 °C; IR (KBr, cm^−1^): 3415, 2934, 1763, 1691, 1619, 1467, 1384, 1120, 939; ^1^H NMR (500 MHz, CDCl3): δ 7.43 (s, 1H), 7.24–7.35 (m, 3H), 6.72 (s, 1H), 5.46 (d, *J* = 1.4 Hz, 1H), 3.95 (d, *J* = 15.1 Hz, 1H), 3.84 (d, *J* = 15.1 Hz, 1H), 3.65–3.71 (m, 1H), 3.63 (s, 3H), 3.45 (s, 3H), 3.23–3.41 (m, 3H), 3.17–3.23 (m, 1H), 2.93 (s, 1H), 2.63 (d, *J* = 16.1 Hz, 1H), 2.47 (dd, *J* = 12.2, 2.7 Hz, 1H), 2.42 (s, 3H), 2.25–2.40 (m, 5H), 2.00 (t, *J* = 11.2 Hz, 1H), 1.70–1.85 (m, 1H), 1.44–1.56 (m, 5H), 1.37–1.45 (m, 2H) (Figure S13); ^13^C NMR (126 MHz, CDCl_3_): *δ* 192.2, 168.8, 152.5, 148.9, 138.5, 135.2, 134.3, 133.5, 129.9, 129.8, 129.7, 129.5, 128.1, 127.4, 115.2, 113.4, 62.0, 56.3, 55.8, 54.8, 54.5, 50.1, 46.7, 45.7, 42.7, 41.0, 40.7, 37.0, 26.2, 24.5, 20.9 (Figure S14). 

(4bR,8aS,9S)-3,7-dimethoxy-11-methyl-6-oxo-1-(piperidin-1-ylmethyl)-6,8a,9,10-tetrahydro-5H-9,4b-(epiminoethano)phenanthren-4-yl 2-(4-methoxyphenyl)acetate (**5h**)

White solid; Yield: 558.3 mg, 81%; m.p.:121–122 °C; IR (KBr, cm^−1^): 3419, 2936, 1760, 1693, 1614, 1514, 1470, 1384, 1250, 1120, 1028, 865; ^1^H NMR (500 MHz, CDCl3): δ 7.35 (d, *J* = 8.5 Hz, 2H), 6.90 (d, *J* = 8.7 Hz, 2H), 6.71 (s, 1H), 5.45 (d, *J* = 1.7 Hz, 1H), 3.89 (d, *J* = 15.1 Hz, 1H), 3.78–3.86 (m, 4H), 3.64–3.72 (m, 1H), 3.62 (s, 3H), 3.46 (s, 3H), 3.22–3.40 (m, 3H), 3.16–3.22 (m, 1H), 2.90 (s, 1H), 2.57–2.72 (m, 1H), 2.42–2.48 (m, 1H), 2.37–2.48 (m, 3H), 2.23–2.36 (m, 5H), 1.90–2.06 (m, 1H), 1.59–1.76 (m, 1H), 1.44–1.54 (m, 5H), 1.38–1.44 (m, 2H) (Figure S15); ^13^C NMR (125 MHz, CDCl3): δ 192.3, 169.9, 158.9, 152.5, 149.0, 138.8, 133.3, 130.8, 129.0, 129.6, 125.4, 115.3, 114.0, 113.5, 62.0, 56.3, 55.9, 55.3, 54.8, 54.5, 50.0, 46.7, 45.8, 42.7, 40.7, 40.6, 36.9, 26.3, 24.5, 20.9 (Figure S16). 

(4bR,8aS,9S)-3,7-dimethoxy-11-methyl-6-oxo-1-(piperidin-1-ylmethyl)-6,8a,9,10-tetrahydro-5H-9,4b-(epiminoethano)phenanthren-4-yl 2-(2,4-dichlorophenyl)acetate (**5i**)

White solid; Yield: 667.5 mg, 90%; m.p.:135–137 °C; IR (KBr, cm^−1^): 3411, 2930, 1766, 1694, 1625, 1473, 1384, 1202, 1098, 869; ^1^H NMR (500 MHz, CDCl3): δ 7.50–7.35 (m, 2H), 7.28 (dd, *J* = 7.8, 2.5 Hz, 1H), 6.74 (s, 1H), 5.47 (d, *J* = 1.7 Hz, 1H), 4.09 (d, *J* = 16.6 Hz, 1H), 4.03 (d, *J* = 16.5 Hz, 1H), 3.74 (d, *J* = 16.0 Hz, 1H), 3.69 (s, 3H), 3.46 (s, 3H), 3.22–3.41 (m, 3H), 3.18–3.23 (m, 1H), 2.96 (s, 1H), 2.63 (dd, *J* = 18.4, 4.9 Hz, 1H), 2.47 (dt, *J* = 9.4, 4.7 Hz, 1H), 2.39–2.45 (m, 4H), 2.24–2.37 (s, 4H), 1.93–2.09 (m, 1H), 1.82 (td, *J* = 12.5, 4.5 Hz, 1H), 1.61 (d, *J* = 12.5 Hz, 1H), 1.48–1.46 (m, 4H), 1.39–1.45 (m, 2H) (Figure S17); ^13^C NMR (125 MHz, CDCl3): δ 192.2, 168.0, 152.5, 148.9, 138.6, 135.2, 134.0, 133.6, 133.0, 130.5, 129.9, 129.5, 129.2, 127.4, 115.3, 113.4, 62.0, 56.3, 56.0, 54.8, 54.5, 50.2, 46.7, 45.8, 42.8, 40.8, 38.6, 37.2, 26.3, 24.5, 20.9 (Figure S18). 

(4bR,8aS,9S)-3,7-dimethoxy-11-methyl-6-oxo-1-(piperidin-1-ylmethyl)-6,8a,9,10-tetrahydro-5H-9,4b-(epiminoethano)phenanthren-4-yl 2-(3,4-dichlorophenyl)acetate (**5j**)

White solid; Yield: 262.3 mg, 52%; m.p.:143–146 °C; IR (KBr, cm^−1^): 3415, 2933, 1766, 1693, 1616, 1471, 1384, 1120, 945; ^1^H NMR (500 MHz, CDCl3): δ 7.54 (d, *J* = 1.5 Hz, 1H), 7.44 (d, *J* = 8.2 Hz, 1H), 7.27–7.34 (m, 1H), 6.72 (s, 1H), 5.47 (d, *J* = 1.5 Hz, 1H), 3.94 (d, *J* = 15.4 Hz, 1H), 3.82 (d, *J* = 15.4 Hz, 1H), 3.69 (d, *J* = 16.4 Hz, 1H), 3.63 (s, 3H), 3.46 (s, 3H), 3.24–3.41 (m, 3H), 3.17–3.22 (m, 1H), 2.93 (s, 1H), 2.62 (d, *J* = 16.4 Hz, 1H), 2.44–2.49 (m, 1H), 2.39–2.44 (m, 4H), 2.22–2.38 (m, 4H), 1.99 (t, *J* = 11.0 Hz, 1H), 1.85–1.73 (m, 1H), 1.45–1.57 (m, 5H), 1.35–1.46 (m, 2H) (Figure S19); ^13^C NMR (125 MHz, CDCl_3_): δ = 192.2, 168.4, 152.4, 148.7, 138.3, 133.6, 133.5, 132.4, 131.5, 131.4, 130.4, 129.8, 129.6, 129.3, 115.3, 113.2, 62.0, 56.2, 55.8, 54.8, 54.5, 50.3, 46.5, 45.8, 42.7, 40.8, 40.4, 37.1, 26.2, 24.5, 20.8 (Figure S20).

(4bR,8aS,9S)-3,7-dimethoxy-11-methyl-6-oxo-1-(piperidin-1-ylmethyl)-6,8a,9,10-tetrahydro-5H-9,4b-(epiminoethano)phenanthren-4-yl 2-(4-fluorophenyl)acetate (**5k**)

White solid; Yield: 667.5 mg, 90%; m.p.:128–130 °C; IR (KBr, cm^−1^): 3416, 2932, 1761, 1693, 1627, 1510, 1468, 1221, 1119, 1025, 865; ^1^H NMR (500 MHz, CDCl3): δ 7.40 (dd, *J* = 7.4, 5.7 Hz, 2H), 7.04 (t, *J* = 8.7 Hz, 2H), 6.70 (s, 1H), 5.46 (s, 1H), 3.93 (d, *J* = 15.1 Hz, 1H), 3.83 (d, *J* = 15.1 Hz, 1H), 3.67 (d, *J* = 16.0 Hz, 1H), 3.60 (s, 3H), 3.45 (s, 3H), 3.23–3.39 (m, 3H), 3.19 (s, 1H), 2.91 (s, 1H), 2.57–2.69 (m, 1H), 2.43–2.47 (m, 1H), 2.41 (s, 3H), 2.23–2.38 (m, 5H), 1.99 (t, *J* = 11.3 Hz, 1H), 1.74 (dd, *J* = 12.0, 8.5 Hz, 1H), 1.55–1.45 (m, 5H), 1.37–1.44 (m, 2H) (Figure S21); ^13^C NMR (125 MHz, CDCl3): δ 192.2, 169.2, 162.1 (d, *J* = 244.3 Hz), 152.5, 148.9, 138.6, 133.4, 131.3 (d, *J* = 8.0 Hz), 129.9, 129.6, 129.0, 115.3 (d, *J* = 21.1 Hz), 115.2, 113.4, 62.0, 56.2, 55.8, 54.7, 54.5, 50.1, 46.6, 45.8, 42.7, 40.7, 40.6, 37.0, 26.2, 24.6, 20.8 (Figure S22). 

#### 3.1.2. General Procedure for the Synthesis of Compounds **6a**–**6l**

To a mixture of 1-(1-pyrrolidinylmethyl)-Sinomenine or 1-(1-piperidinylmethyl)-Sinomenine (1.0 mmol) and Et_3_N (2.5 mmol) in CH_2_Cl_2_ (5 mL) was added acyl chloride (2.5 mmol) at 0 °C. The reaction mixture was then stirred at room temperature. After completion of the reaction, sat. NaHCO_3_ was added and then extracted with CH_2_Cl_2_. The organic layers were combined and dried with anhydrous Na_2_SO_4_ and evaporated under reduced pressure. The mixture was evaporated under vacuum, and the residue was purified by flash chromatography with dichloromethane and methanol as the eluent to give the pure product.

(4bR,8aS,9S)-3,7-dimethoxy-11-methyl-6-oxo-1-(pyrrolidin-1-ylmethyl)-6,8a,9,10-tetrahydro-5H-9,4b-(epiminoethano)phenanthren-4-yl 2-(4-chlorophenyl)acetate (**6a**)

White solid; Yield: 239.5 mg, 23%; m.p.:162–165 °C; IR (KBr, cm^−1^): 3465, 1634, 1384, 1217, 1089, 771, 534; ^1^H NMR (500 MHz, CDCl3): *δ* 7.39 (d, *J* = 8.1 Hz, 2H), 7.34 (d, *J* = 8.1 Hz, 2H), 6.77 (s, 1H), 5.47 (s, 1H), 3.94 (d, *J* = 15.1 Hz, 1H), 3.84 (d, *J* = 15.1 Hz, 1H), 3.68 (d, *J* = 16 Hz, 1H), 3.62 (s, 3H), 3.52 (br, 2H), 3.46 (s, 3H), 3.21 (br, 2H), 2.93 (br, 1H), 2.67 (d, *J* = 15.9 Hz, 1H), 2.45–2.40 (m, 5H), 2.40 (s, 3H), 2.01 (dd, *J* = 11.7, 10.7 Hz, 1H), 1.83 (br, 2H), 1.74 (br, 4H), 1.49 (d, *J* = 10.7 Hz, 1H) (Figure S24); ^13^C NMR (125 MHz, CDCl_3_): δ 192.4, 169.1, 152.4, 148.9, 138.4, 134.4, 133.1, 131.8, 131.1, 129.8, 129.0, 128.6, 115.2, 112.6, 58.5, 56.1, 55.8, 54.7, 54.0, 50.1, 46.6, 45.7, 42.7, 40.8, 40.7, 36.9, 23.6, 20.8 (Figure S25). HRMS (ESI) *m*/*z* [M+H]^+^ calcd for C_32_H_38_ClN_2_O_5_ 565.24637, found 565.24548 (Figure S23).

(4bR,8aS,9S)-3,7-dimethoxy-11-methyl-6-oxo-1-(pyrrolidin-1-ylmethyl)-6,8a,9,10-tetrahydro-5H-9,4b-(epiminoethano)phenanthren-4-yl 2-(3,4-dimethoxyphenyl)acetate (**6b**)

Yellow solid; Yield: 780.3 mg, 37%; m.p.: 80–83 °C; IR (KBr, cm^−1^): 3466, 3018, 2958, 2839, 1759, 1688, 1632, 1515, 1465, 1384, 1263, 1145, 1107, 1026, 758, 666, 540; ^1^H NMR (500 MHz, CDCl3): *δ* 7.02 (s, 1H), 6.97 (dd, *J* = 8.2, 1.9 Hz, 1H), 6.87 (d, *J* = 8.2 Hz, 1H), 6.78 (s, 1H), 5.44 (d, *J* = 1.8 Hz, 1H), 3.94 (s, 3H), 3.88 (s, 3H), 3.87 (d, *J* = 14.5 Hz, 1H), 3.82 (d, *J* = 14.5 Hz, 1H), 3.65 (s, 3H), 3.57 (d, *J* = 15.8 Hz, 1H), 3.51 (br, 2H), 3.44 (s, 3H), 3.14–3.10 (m, 2H), 2.89–2.86 (m, 1H), 2.67 (dd, *J* = 18.2, 3.7 Hz, 1H), 2.45–2.40 (m, 5H), 2.38 (s, 3H), 2.23 (d, *J*=16.1, 1H), 2.00 (t, *J* = 11.2 Hz, 1H), 1.76–1.71 (m, 4H), 1.65 (td, *J* = 13.2, 4.0 Hz, 1H), 1.44–1.42(m, 1H) (Figure S27); ^13^C NMR (125 MHz, CDCl_3_): *δ* 192.5, 169.6, 152.3, 149.1, 149.0, 148.3, 138.5, 134.3, 129.9, 128.9, 125.6, 121.9, 115.1, 113.0, 112.6, 111.3, 58.5, 56.2, 55.9, 54.7, 54.1, 49.6, 46.7, 45.6, 42.7, 41.2, 40.5, 36.7, 23.6, 20.9 (Figure S28). HRMS (ESI) *m*/*z* [M+H]^+^ calcd for C_34_H_43_N_2_O_7_ 591.3064781, found 591.30579 (Figure S26). 

(4bR,8aS,9S)-3,7-dimethoxy-11-methyl-6-oxo-1-(pyrrolidin-1-ylmethyl)-6,8a,9,10-tetrahydro-5H-9,4b-(epiminoethano)phenanthren-4-yl 2-(2-chlorophenyl)acetate (**6c**)

White solid; Yield: 432.5 mg, 29%; m.p.: 79–83 °C; IR (KBr, cm^−1^): 3423, 3013, 2932, 2840, 2801, 1765, 1690, 1629, 1469, 1444, 1414, 1379, 1344, 1320, 1202, 1122, 1021, 990, 859, 754, 665, 558; ^1^H NMR (500 MHz, CDCl3): *δ* 7.48 (d, *J* = 7.6 Hz, 1H), 7.41 (dd, *J* = 7.6, 1.5 Hz, 1H), 7.28 (td, *J* = 7.6, 1.6 Hz, 1H), 7.24 (td, *J* = 7.6, 1.6 Hz, 1H), 6.78 (s, 1H), 5.46 (d, *J* = 1.9 Hz, 1H), 4.13 (d, *J* = 16.5 Hz, 1H), 4.06 (d, *J* = 16.5 Hz, 1H), 3.75 (d, *J* = 16 Hz, 1H), 3.70 (s, 3H), 3.55 (d, *J* = 12.5 Hz, 1H), 3.50 (d, *J* = 12.3 Hz, 1H), 3.45 (s, 3H), 3.25–3.20 (m, 2H), 2.98–2.95 (m, 1H), 2.69 (dd, *J* = 18.2, 4.4 Hz, 1H), 2.50 (ddd, *J* = 12.2, 4.4, 1.8 Hz, 1H), 2.46–2.37 (m, 8H), 2.03 (td, *J* = 12.2, 3.3 Hz, 1H), 1.82 (td, *J* = 12.7, 4.6 Hz, 1H), 1.76–1.69 (m, 4H), 1.64 (dt, *J* = 12.5, 4.6 Hz, 1H) (Figure S30); ^13^C NMR (125 MHz, CDCl_3_): δ 192.3, 168.3, 152.3, 149.1, 138.6, 134.5,134.2, 132.1, 131.7, 129.7, 129.4, 128.8, 128.7, 127.0, 115.0, 112.7, 58.5, 56.1, 55.9, 54.7, 54.0, 49.8, 46.6, 45.4, 42.5, 40.6, 39.1, 36.8, 23.6, 20.9 (Figure S31). HRMS (ESI) *m*/*z* [M+H]^+^ calcd for C_32_H_38_ClN_2_O_5_ 565.24637, found 565.24530 (Figure S29). 

(4bR,8aS,9S)-3,7-dimethoxy-11-methyl-6-oxo-1-(pyrrolidin-1-ylmethyl)-6,8a,9,10-tetrahydro-5H-9,4b-(epiminoethano)phenanthren-4-yl 2-(2,4-dichlorophenyl)acetate (**6d**)

White solid; Yield: 342.8 mg, 22%; m.p.: 82–86 °C; IR (KBr, cm^−1^): 3451, 2927, 1765, 1688, 1633, 1474, 1384, 1202, 1125, 1099, 990, 928, 770, 524; ^1^H NMR (500 MHz, CDCl3): δ 7.47–7.39 (m, 2H), 7.27 (dd, *J* = 8.2, 2.1 Hz, 1H), 6.78 (s, 1H), 5.46 (d, *J* = 1.7 Hz, 1H), 4.09 (d, *J* = 16.5 Hz,1H), 4.02 (d, *J* = 16.5 Hz, 1H), 3.73 (d, *J* = 16.4 Hz, 1H), 3.69 (s, 3H), 3.56 (d, *J* = 11.7 Hz, 1H), 3.50 (d, *J* = 11.7 Hz, 1H), 3.45 (s, 3H), 3.28–3.17 (m, 2H), 2.97 (br, 1H), 2.69 (d, *J* = 16.2 Hz, 1H), 2.51 (dd, *J* = 11.8, 2.3 Hz, 1H), 2.46–2.41 (m, 8H), 2.03 (t, *J* = 12.2 Hz, 1H), 1.84 (td, *J* = 12.5, 4.3 Hz, 1H), 1.76–1.71 (m, 4H), 1.61 (d, *J* = 12.3 Hz, 1H) (Figure S33); ^13^C NMR (125 MHz, CDCl_3_): δ 192.2, 168.0, 152.4, 149.0, 138.4, 135.2, 134.3, 133.9, 132.9, 130.4, 129.7, 129.2, 128.8, 127.4, 115.1, 112.7, 58.5, 56.1, 55.9, 54.7, 54.0, 50.0, 46.6, 45.5, 42.5, 40.6, 38.5, 36.9, 23.6, 20.9 (Figure S34). HRMS (ESI) *m*/*z* [M+H]^+^ calcd for C_32_H_37_Cl_2_N_2_O_5_ 599.20740, found 599.20679 (Figure S32).

(4bR,8aS,9S)-3,7-dimethoxy-11-methyl-6-oxo-1-(pyrrolidin-1-ylmethyl)-6,8a,9,10-tetrahydro-5H-9,4b-(epiminoethano)phenanthren-4-yl 2-(3-chlorophenyl)acetate (**6e**)

White solid; Yield: 765.6 mg, 51%; m.p.: 68–72 °C; IR (KBr, cm^−1^): 3419, 2959, 1760, 1635, 1470, 1384, 1216, 1106, 990, 770, 524; ^1^H NMR (500 MHz, CDCl3): *δ* 7.45–7.43 (m, 1H), 7.34–7.26 (m, 3H), 6.78 (s, 1H), 5.46 (d, *J* = 1.9 Hz, 1H), 3.95 (d, *J* = 15.1 Hz,1H), 3.85 (d, *J* = 15.1 Hz, 1H), 3.67 (d, *J* = 16.1 Hz, 1H), 3.64 (s, 3H), 3.52 (br, 2H), 3.45 (s, 3H), 3.23–3.19 (m, 2H), 2.95–2.92 (m, 1H), 2.67 (d, *J* = 15.1 Hz, 1H), 2.49–2.40 (m, 9H), 2.02 (t, *J* = 13.1 Hz, 1H), 1.78 (dd, *J* = 12.5, 4.4 Hz, 1H), 1.76–1.70 (m, 4H), 1.50 (d, *J* = 10.4 Hz, 1H) (Figure S36); ^13^C NMR (125 MHz, CDCl_3_): δ 192.3, 168.9, 152.4, 149.0, 138.4, 135.2, 134.4, 134.2, 129.8, 129.7, 129.6, 128.9, 128.1, 127.4, 115.2, 112.6, 58.5, 56.1, 55.8, 54.7, 54.0, 50.0, 46.6, 45.6, 42.6, 41.0, 40.7, 36.8, 23.6, 20.8 (Figure S37). HRMS (ESI) *m*/*z* [M+H]^+^ calcd for C_32_H_38_ClN_2_O_5_ 565.24637, found 565.24530 (Figure S35). 

(4bR,8aS,9S)-3,7-dimethoxy-11-methyl-6-oxo-1-(pyrrolidin-1-ylmethyl)-6,8a,9,10-tetrahydro-5H-9,4b-(epiminoethano)phenanthren-4-yl 2-(o-tolyl)acetate (**6f**)

White solid; Yield: 600.5 mg, 42%; m.p.: 72–75 °C; IR (KBr, cm^−1^): 3417, 2958, 1755, 1635, 1463, 1384, 1217, 1091, 990, 771, 530; ^1^H NMR (500 MHz, CDCl3): *δ* 7.38–7.32 (m, 1H), 7.23–7.18 (m, 3H), 6.76 (s, 1H), 5.44 (d, *J* = 1.9 Hz, 1H), 3.96 (d, *J* = 15.4 Hz, 1H), 3.91 (d, *J* = 15.4 Hz, 1H), 3.63 (s, 3H), 3.58 (d, *J* = 16.1 Hz, 1H), 3.51 (br, 2H), 3.44 (s, 3H), 3.26–3.10 (m, 2H), 2.92–2.88 (m, 1H), 2.68 (d, *J* = 16.0 Hz, 1H), 2.45–2.39 (m, 11H), 2.28 (d, *J* = 16.1 Hz, 1H), 2.01 (t, *J* = 11.6 Hz, 1H), 1.75–1.70 (m, 5H), 1.50 (d, *J* = 12.1 Hz, 1H) (Figure S39); ^13^C NMR (125 MHz, CDCl_3_): *δ* 192.3, 169.3, 152.3, 149.1, 138.5, 137.3, 134.2, 131.9, 130.6, 130.4, 129.8, 128.9, 127.4, 126.0, 115.1, 112.6, 58.5, 56.2, 55.8, 54.7, 54.0, 49.7, 46.7, 45.6, 42.7, 40.5, 39.2, 36.8, 23.6, 20.8. 19.6 (Figure S40). HRMS (ESI) *m*/*z* [M+H]^+^ calcd for C_33_H_41_N_2_O_5_ 545.30099, found 545.30072 (Figure S38).

(4bR,8aS,9S)-3,7-dimethoxy-11-methyl-6-oxo-1-(pyrrolidin-1-ylmethyl)-6,8a,9,10-tetrahydro-5H-9,4b-(epiminoethano)phenanthren-4-yl 2-(3,4-dichlorophenyl)acetate (**6g**)

White solid; Yield: 917.1 mg, 57%; m.p.: 160–164 °C; IR (KBr, cm^−1^): 3416, 2926, 1761, 1689, 1636, 1618, 1470, 1384, 1201, 1106, 1032, 753, 618, 482; ^1^H NMR (500 MHz, CDCl3): *δ* 7.56–7.53 (m, 1H), 7.44 (d, *J* = 8.1 Hz, 1H), 7.30 (d, *J* = 8.1 Hz, 1H), 6.78 (s, 1H), 5.47 (d, *J* = 1.7 Hz, 1H), 3.94 (d, *J* = 15.3 Hz, 1H), 3.82 (d, *J* = 15.3 Hz, 1H), 3.68 (d, *J* = 16.0 Hz, 1H), 3.64 (s, 3H), 3.52 (br, 2H), 3.45 (s, 3H), 3.21 (t, *J* = 4.3 Hz, 2H), 2.95–2.92 (m, 1H), 2.66 (d, *J* = 16.6 Hz, 1H), 2.48–2.40 (m, 9H), 2.04–2.00 (m, 1H), 1.79 (td, *J* = 12.5, 4.4 Hz, 1H), 1.75–1.70 (m, 4H), 1.51 (d, *J* = 12.1 Hz, 1H) (Figure S42); ^13^C NMR (125 MHz, CDCl_3_): *δ* 192.2, 168.4, 152.4, 148.8, 138.2, 134.5, 133.5, 132.3, 131.5, 131.3, 130.4, 129.7, 129.3, 129.0, 115.3, 112.5, 58.5, 56.1, 55.8, 54.7, 54.0, 50.2, 46.6, 45.7, 42.7, 40.7, 40.4, 37.0, 23.6, 20.8 (Figure S43). HRMS (ESI) *m*/*z* [M+H]^+^ calcd for C_32_H_37_Cl_2_N_2_O_5_ 599.20740, found 599.20667 (Figure S41). 

(4bR,8aS,9S)-3,7-dimethoxy-11-methyl-6-oxo-1-(pyrrolidin-1-ylmethyl)-6,8a,9,10-tetrahydro-5H-9,4b-(epiminoethano)phenanthren-4-yl 2-phenylacetate (**6h**)

White solid; Yield: 156.0 mg, 27%; m.p.: 165–168 °C; IR (KBr, cm^−1^): 3417, 2958, 1760, 1687, 1636, 1616, 1464, 1413, 1384, 1215, 1148, 1107, 940, 770, 621, 481; ^1^H NMR (500 MHz, CDCl3): *δ* 7.47–7.42 (m, 2H), 7.38–7.35 (m, 2H), 7.31–7.28 (m, 1H), 6.77 (s, 1H), 5.44 (d, *J* = 1.8 Hz, 1H), 3.96 (d, *J* = 14.9 Hz, 1H), 3.88 (d, *J* = 14.9 Hz, 1H), 3.66 (d, *J* = 16.2 Hz, 1H), 3.62 (s, 3H), 3.53 (br, 2H), 3.45 (s, 3H), 3.27–3.19 (m, 2H), 2.96–2.92 (m, 1H), 2.71 (dd, *J* = 17.9, 4.0 Hz, 1H), 2.51–2.41 (m, 8H), 2.31 (d, *J* = 15.9 Hz, 1H), 2.07–2.03 (m, 1H), 1.78–1.70 (m, 5H), 1.48 (d, *J* = 11.1 Hz, 1H) (Figure S45); ^13^C NMR (125 MHz, CDCl_3_): *δ* 192.3, 169.7, 152.4, 149.2, 138.6, 134.2, 133.2, 129.8, 128.7, 128.6, 127.2, 114.9, 112.8, 58.5, 56.2, 55.9, 54.8, 54.0, 49.7, 46.7, 45.3, 42.5, 41.5, 40.4, 36.6, 23.6, 20.9 (Figure S46). HRMS (ESI) *m*/*z* [M+H]^+^ calcd for C_32_H_39_N_2_O_5_ 531.28534, found 531.28503 (Figure S44). 

(4bR,8aS,9S)-3,7-dimethoxy-11-methyl-6-oxo-1-(pyrrolidin-1-ylmethyl)-6,8a,9,10-tetrahydro-5H-9,4b-(epiminoethano)phenanthren-4-yl 2-(4-methoxyphenyl)acetate (**6i**)

White solid; Yield: 282.1 mg, 36%; m.p.: 76–79 °C; IR (KBr, cm^−1^): 3435, 2959, 2838, 1760, 1685, 1630, 1514, 1466, 1382, 1322, 1302, 1250, 1202, 1180, 1148, 1105, 1026, 989, 537; ^1^H NMR (500 MHz, CDCl3): *δ* 7.33 (d, *J* = 8.4 Hz, 2H), 6.87 (d, *J* = 8.4 Hz, 2H), 6.74 (s, 1H), 5.43 (d, *J* =1.0 Hz, 1H), 3.87 (d, *J* = 15.1 Hz, 1H), 3.79 (d, *J* = 15.1 Hz, 1H), 3.76 (s, 3H), 3.65 (d, *J* = 16.1 Hz, 1H), 3.60 (s, 3H), 3.49 (br, 2H), 3.41 (s, 3H), 3.21–3.10 (m, 2H), 2.87 (br, 1H), 2.65 (d, *J* = 15.5 Hz, 1H), 2.45–2.37 (m, 5H), 2.36 (s, 3H), 2.28 (d, *J* = 16.1 Hz, 1H), 1.98 (t, *J* = 11.3 Hz, 1H), 1.74–1.63 (m, 5H), 1.49 (d, *J* = 10.7 Hz, 1H) (Figure S48); ^13^C NMR (125 MHz, CDCl_3_): *δ* 192.3, 169.6, 158.7, 152.2, 148.9, 138.4, 134.1, 130.6, 129.7, 128.8, 125.2, 115.1, 113.8, 112.5, 58.4, 56.0, 55.7, 55.1, 54.6, 53.9, 49.7, 46.6, 45.5, 42.5, 40.5, 40.4, 36.6, 23.5, 20.7 (Figure S49). HRMS (ESI) *m*/*z* [M+H]^+^ calcd for C_33_H_41_N_2_O_6_ 561.29591, found 561.29486 (Figure S47). 

(4bR,8aS,9S)-3,7-dimethoxy-11-methyl-6-oxo-1-(pyrrolidin-1-ylmethyl)-6,8a,9,10-tetrahydro-5H-9,4b-(epiminoethano)phenanthren-4-yl 2-(4-nitrophenyl)acetate (**6j**)

Yellow solid; Yield: 71.6 mg, 7% yield; m.p.: 82–85 °C; IR (KBr, cm^−1^): 3450, 2962, 1762, 1685, 1630, 1520, 1466, 1383, 1347, 1203, 1182, 1122, 1018, 989, 857, 560; ^1^H NMR (500 MHz, CDCl3): *δ* 8.24 (d, *J* = 8.7 Hz, 2H), 7.65 (d, *J* = 8.7 Hz, 2H), 6.78 (s, 1H), 5.48 (d, *J* =1.4 Hz, 1H), 4.10 (d, *J* = 15.4 Hz, 1H), 3.98 (d, *J* = 15.4 Hz, 1H), 3.67 (d, *J* = 15.9 Hz, 1H), 3.61 (s, 3H), 3.52 (br, 2H), 3.46 (s, 3H), 3.28–3.16 (m, 2H), 2.98–2.93 (m, 1H), 2.67 (d, 1H, *J* =17.0 Hz) 2.48–2.39 (m, 9H), 2.02 (t, *J* = 11.1 Hz, 1H), 1.81 (td, *J* =12.4Hz, 1H) 1.77–1.71 (m, 4H), 1.52 (d, *J* = 8.7 Hz, 1H) (Figure S51); ^13^C NMR (125 MHz, CDCl_3_): *δ* 192.1, 169.1, 152.4, 148.8, 147.3, 140.7, 138.2, 134.7, 130.8, 129.7, 129.1, 123.7, 115.3, 112.6, 58.6, 56.1, 55.8, 54.8, 54.1, 50.3, 46.6, 45.8, 42.7, 41.2, 40.8, 37.1, 23.6, 20.8 (Figure S52). HRMS (ESI) *m*/*z* [M+H]^+^ calcd for C_32_H_38_N_3_O_7_ 576.27042, found 576.26965 (Figure S50). 

(4bR,8aS,9S)-3,7-dimethoxy-11-methyl-6-oxo-1-(pyrrolidin-1-ylmethyl)-6,8a,9,10-tetrahydro-5H-9,4b-(epiminoethano)phenanthren-4-yl 2-(2-methoxyphenyl)acetate (**6k**)

White solid; Yield: 239.5 mg, 19%; m.p.: 180–182 °C; IR (KBr, cm^−1^): 3443, 3023, 3007, 2965, 2926, 2849, 2790, 1766, 1700, 1622, 1606, 1498, 1467, 1340, 1291, 1249, 1201, 1128, 1112, 1091, 1023, 991, 929, 766, 745, 534, 492; ^1^H NMR (500 MHz, CDCl3): *δ* 7.33 (d, *J* = 7.1 Hz, 1H), 7.28 (td, J = 7.1, 1.5 Hz, 1H), 6.96 (t, *J* = 7.1 Hz, 1H), 6.91 (d, *J* = 7.1 Hz, 1H), 6.78 (s, 1H), 5.45 (d, *J* =1.7 Hz, 1H), 4.06 (d, *J* = 15.9 Hz, 1H), 3.87 (s, 3H), 3.84 (d, *J* = 15.9 Hz, 1H), 3.77 (d, *J* = 16.1 Hz, 1H), 3.71 (s, 3H), 3.57–3.48 (m, 2H), 3.45 (s, 3H), 3.25–3.13 (m, 2H), 2.95–2.89 (m, 1H), 2.70 (dd, *J* = 18.4, 5.2 Hz, 1H), 2.49–2.37 (m, 8H), 2.31 (d, *J* = 16.1 Hz, 1H), 2.03 (td, *J* = 11.6, 1.7 Hz, 1H), 1.79–1.70 (m, 5H), 1.63 (dt, *J* = 12.4, 2.5 Hz, 1H) (Figure S54); ^13^C NMR (125 MHz, CDCl_3_): *δ* 192.5, 169.7, 157.5, 152.3, 149.2, 138.7, 134.1, 131.3, 129.9, 128.9, 128.6, 122.2, 120.4, 115.1, 112.6, 110.4, 58.5, 56.2, 56.0, 55.3, 54.7, 54.0, 49.4, 46.8, 45.7, 42.7, 40.5, 36.6, 35.8, 23.6, 20.8 (Figure S55). HRMS (ESI) *m*/*z* [M+H]^+^ calcd for C_33_H_41_N_2_O_6_ 561.29591, found 561.29504 (Figure S53). 

(4bR,8aS,9S)-3,7-dimethoxy-11-methyl-6-oxo-1-(pyrrolidin-1-ylmethyl)-6,8a,9,10-tetrahydro-5H-9,4b-(epiminoethano)phenanthren-4-yl 2-(2-fluorophenyl)acetate (**6l**)

White solid; Yield: 210.3 mg, 21%; m.p.: 178–180 °C; IR (KBr, cm^−1^): 3445, 2965, 2924, 2892, 2800, 1752, 1687, 1633, 1604, 1495, 1471, 1459, 1414, 1373, 1320, 1305, 1295, 1237, 1200, 1146, 1107, 1033, 926, 899, 876, 851, 767, 665, 613, 565; ^1^H NMR (500 MHz, CDCl3): *δ* 7.43 (t, *J* = 7.5 Hz, 1H), 7.31–7.24 (m, 1H), 7.15 (td, *J* = 7.5, 1.0 Hz, 1H), 7.09 (dd, *J* = 7.5, 1.0 Hz, 1H), 6.78 (s, 1H), 5.46 (d, *J* =1.9 Hz, 1H), 4.02 (d, *J* = 16.4 Hz, 1H), 3.96 (d, *J* = 16.4 Hz, 1H), 3.75 (d, *J* = 16.0 Hz, 1H), 3.69 (s, 3H), 3.52 (br, 2H), 3.45 (s, 3H), 3.27–3.14 (m, 2H), 2.95–2.91 (m, 1H), 2.67 (dd, *J* = 18.0, 3.9 Hz, 1H), 2.49–2.38 (m, 9H), 2.01 (t, *J* = 11.7, 1.7 Hz, 1H), 1.80 (td, *J* = 12.5, 4.6 Hz, 1H), 1.76–1.70 (m, 4H), 1.59 (d, *J* = 12.5 Hz, 1H) (Figure S57); ^13^C NMR (125 MHz, CDCl_3_): *δ* 192.4, 168.7, 161.1 (d, *J* = 245.3 Hz), 152.3, 149.0, 138.5, 134.3, 132.0 (d, *J* = 3.8 Hz), 129.8, 129.2 (d, *J* = 8.1 Hz), 129.0, 124.2 (d, *J* = 3.7 Hz), 120.8 (d, *J* = 15.7 Hz), 115.4, 115.2, 112.6, 58.6, 56.2, 56.0, 54.7, 54.0, 49.9, 46.7, 45.7, 42.7, 40.7, 36.9, 34.6, 23.6, 20.8 (Figure S58). HRMS (ESI) *m*/*z* [M+H]^+^ calcd for C_32_H_38_FN_2_O_5_ 549.27592, found 549.27588 (Figure S56).

### 3.2. Biological Activity

#### 3.2.1. Drugs and Drug Treatments

Cisplatin was purchased from Biyuntian (Shanghai, China), CAS: 15663-27-1, purity >99%; dimethyl sulfoxide (DMSO) was purchased from Bailingwei (Beijing, China); synthetic and purchased positive control compounds under investigation were dissolved in DMSO to produce a stock solution.

#### 3.2.2. Cell Lines

The MCF-7 (human breast cancer cell lines), Hela (human cervical cancer cells lines), and HepG2 (human hepatocellular carcinoma cell lines) cells were used to measure the cytotoxicity of the **5a**–**5k** series, which were provided by the Academy of Military Medical Sciences (Beijing, China). The cell lines were maintained on RPMI 1640 nutrient medium supplemented with 10% heat-inactivated fetal bovine serum, 1% L-glutamine, 100 mg/mL streptomycin and 100 units/mL penicillin. The cells were grown at 37 °C in a humidified atmosphere with 5% CO_2_ and were subcultured two to three times a week.

The MCF-7 (human breast cancer cell lines), Hela (human cervical cancer cell lines), SW480 (human colon adenocarcinoma cell lines), A549 (human lung adenocarcinoma cell lines) and nontoxic normal cells Hek293 (human embryonic kidney cell lines) cells were used to measure the cytotoxicity of the **6a**–**6l**. They were obtained from the Beijing Normal University (Beijing, China). The test cell lines were cultured in DMEM, IMDM, F12 and DMEM nutrient medium containing 10% heat-inactivated fetal bovine serum, 100 mg/mL streptomycin and 100 units/mL penicillin in a humified, 5% (*v*/*v*) CO_2_ atmosphere at 37 °C. 

DMEM culture medium (Dulbecco’s modified essential medium), PBS (Phosphate Buffer Solution), penicillin and streptomycin were purchased from Gibco (Beijing, China). FBS (fetal bovine serum), RPMI 1640, and F12 culture medium were purchased from HyClone (Beijing, China). IMEM culture medium and 0.25% Trypsin-EDTA were purchased from Macgene (Beijing, China). MTT was purchased from Sigma (Beijing, China). CCK8 (Cell Counting Kit-8) was purchased from Dojindo (Shanghai, China).

#### 3.2.3. Anticancer Evaluation 

##### MTT Assay

The MTT (3-(4, 5-dimethyl thiazol-2-yl)-2, 5-diphenyl tetrazolium bromide) assay with a slight modification was used to determine the inhibition effects of substrates **5a**–**5k**. MCF-7, Hela and HepG2 were used for testing. Briefly, cells (5.0 × 10^3^ cells per well) were seeded in 100 μL of the RPMI-1640 medium in 96-well plates for 24 h, treated with drugs and complete medium in wells up to 200 μL for 24 h. RPMI-1640 samples were employed as negative controls, and cisplatin as a positive control. Four quadruplicates of each concentration for all tested compounds were evaluated in three independent assays. For this, after removing the drug containing media 100 μL, 10 μL of MTT solution was added to wells and incubated for 4 h under similar conditions. After that, the supernatant from each well was removed and the formazan crystals formed by viable cells were dissolved with DMSO (100 μL/well). At the end of incubation, optical densities at 570 nm were measured using a plate reader (BIO-TEK, USA). Cell viability was calculated based on the measured absorbance relative to the absorbance of the cells exposed to the negative control, which represented 100% cell viability.

##### CCK8 Assay

Cell viability was estimated using the CCK8 assay. Tumor cells (100 mL) viz. MCF-7, Hela, SW480, A549 and nontoxic normal cells Hek293 were seeded in medium containing Corning^®^ 96-well tissue culture plates at confluences of 20, 15, 20, 15, 40 and 13%, respectively, in Incucyte ZOOM. Fresh medium (100 μL) containing different concentrations of the test sample was added after 12 h of seeding. The microtiter plates were incubated at 37 °C in a humidified incubator with 5% CO_2_ for 60 h. Triplicates from each concentration were used. Negative control cells were incubated without sample and cisplatin as a positive control. At the end of treatment, the numbers of viable cells were determined by the CCK8 test. In brief, the media was removed from the 96 well plates and replaced with 100 μL of fresh culture medium with 10% CCK8 solution added to each well including the untreated controls. The 96 well plates were then incubated at 37 °C and 5% CO2 for 1–4 h. Then, the absorbance was measured at 450 nm using a BMG LABTECH^®^-POLAR star Omega microplate reader (Ortenberg, Germany) to determine the number of viable cells, and the percentage of viability was calculated as ((ODt- ODb/ODc- ODb)) × 100%, where ODt is the mean optical density of wells treated with the tested sample, ODc is the mean optical density of wells treated with DMSO and ODb is the mean optical density of PBS wells without cells. The relation between surviving cells and drug concentrations were plotted to get the survival curve of each tumor cell line after treatment with the specified compound. The 50% inhibitory concentration (IC_50_), the concentration required to cause toxic effects in 50% of intact cells, was estimated from graphic plots of the dose response curve for each concentration [21,22].

### 3.3. In Silico Study

#### 3.3.1. Collection of Related Genes

Multiple disease-related gene databases viz. Online Mendelian Inheritance in Man (OMIM) [23], Human Gene Function and Network Analysis (CooLGeN) [24], The Comparative Toxicogenomics Database (CTD) [25] and GeneCards [26], were used to collect the cancer-associated genes. Either advanced search or custom filtering criteria were used for the gene retrieval. For Cool GeN, genes collected with all human genes were retrieved. For CTD, genes annotated with direct evidence and labeled as “M” (marker/mechanism) and/or “T” (therapeutic) were retrieved. For GeneCards, genes associated with “Protein Coding” were retrieved. 

#### 3.3.2. Pharmacology Network Analysis

STRING [27] was used to construct the protein-protein interaction (PPI) networks of the targets related to the five cancer diseases. Analysis and modularization were performed using Cytoscape. The MCODE algorithm [28] was used to determine highly interconnected regions in the PPI network. The degree cut off, node density cutoff, and node score cutoff were kept to 2, 0.1 and 0.2, respectively.

#### 3.3.3. Enrichment Analysis of Five Cancer Diseases Targets

All targets of five cancer diseases-related cell lines were mapped into Gene Ontology (GO) and Kyoto Encyclopedia of Genes and Genomes (KEGGs) [29]. The GO functional annotations were carried for the biological process (BP), molecular function (MF) and cellular components (CC) terms [30].

#### 3.3.4. Molecular Docking

Molecular docking was performed using iGEMDOCK (BioXGEM, Taiwan) [31]. iGEMDOCK makes use of the genetic algorithm that selects the best solution from the population by calculating their fitness. Genetic algorithm is a general-purpose optimization and search technique which is based on the evolutionary process of natural selection. Genetic algorithm permits the individuals of a population to evolve under certain specific selection rules to a condition that maximizes the fitness function [32]. The PyMOL Molecular Graphics (Version 1.8.4.0, Schrödinger, LLC) was used for the visualizations and graphics generations [33]. To determine the interactions of all the docking complexes, the protein-ligand interaction profiler (PLIP) was used to analyze the crystal structure of the available structural complexes [34].

## 4. Conclusions

In the current work, a series of sinomenine derivatives were synthesized with good yields. All the derivatives were tested for their cytotoxic activity against a variety of cancer cells in vitro and applied to a molecular docking study to investigate the potential molecular targets. The results show that compounds containing chlorine had significant anticancer activity. Furthermore, we obtained 16 core targets and some key signal pathways related to cancer diseases through comprehensive bioinformatics analysis. The molecular docking results showed that AKT1, EGFR, HARS and KARS could be considered as the most potential anticancer targets of sinomenine derivatives with high binding affinity. We found that these targets are important key genes in the PI3K/AKT and MAPK signaling pathways combined with the results of KEGG enrichment. Therefore, we boldly speculate that chlorine-containing sinomenine derivatives may cause the pathological death of cancer cells by regulating multiple genes in these pathways.

As the current amount of the compound is insufficient for more experimental verification, we intend to synthesize chlorine-substituted sinomenine derivatives with different substitutions to conduct a detailed structure-activity relationship (SAR) analysis. We will further explore the relationships between structure and activity combined with activity experiments in vitro. 

## Figures and Tables

**Table 1:** Chemical structures of synthesized sinomenine derivatives (**5a**–**5k**, **6a**–**6l**).

NO.	Compounds	R_1_	R_2_	R_3_	Product	Yield (%)
1	**5a**	H	H	Cl	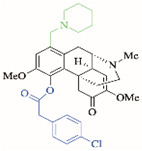	90
2	**5b**	H	H	H	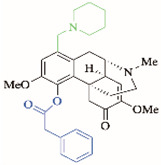	64
3	**5c**	H	OMe	OMe	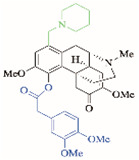	90
4	**5d**	Me	H	H	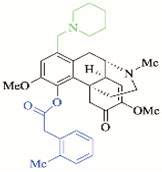	85
5	**5e**	H	H	H	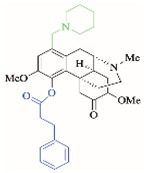	55
6	**5f**	Cl	H	H	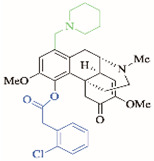	77
7	**5g**	H	Cl	H	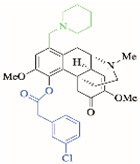	72
8	**5h**	H	H	OMe	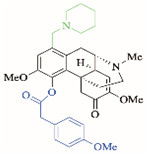	81
9	**5i**	Cl	H	Cl	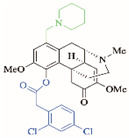	90
10	**5j**	H	Cl	Cl	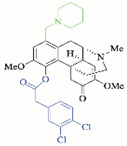	52
11	**5k**	H	H	F	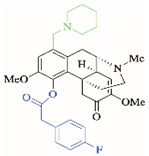	90
12	**6a**	H	H	Cl	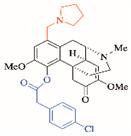	23
13	**6b**	H	OMe	OMe	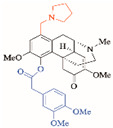	37
14	**6c**	Cl	H	H	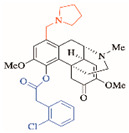	29
15	**6d**	Cl	H	Cl	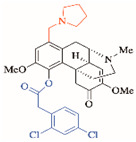	22
16	**6e**	H	Cl	H	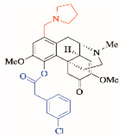	51
17	**6f**	Me	H	H	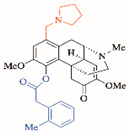	42
18	**6g**	H	Cl	Cl	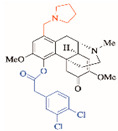	57
19	**6h**	H	H	H	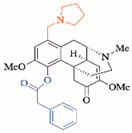	27
20	**6i**	H	H	OMe	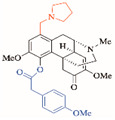	36
21	**6j**	H	H	NO_2_	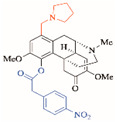	7
22	**6k**	OMe	H	H	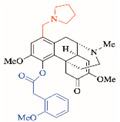	19
23	**6l**	F	H	H	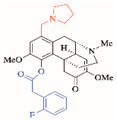	21

**Table 2:** The anticancer activities of the synthesized compounds and standard anticancer agent expressed in terms of IC_50_ (μM) ^a^.

Compounds	MCF-7	Hela	SW480	A549	Hek293
**6a**	14.34 ± 0.83	11.52 ± 1.04	14.94 ± 0.06	>25	12.98 ± 0.62
**6c**	>25	>25	>25	18.72 ± 0.70	6.52 ± 0.19
**6d**	5.73 ± 0.36	8.20 ± 0.52	6.08 ± 0.28	11.57 ± 1.61	3.46 ± 0.02
**6e**	14.86 ± 0.15	13.28 ± 0.95	>25	17.91 ± 0.74	16.57 ± 0.64
**6f**	>25	>25	>25	25.05 ± 1.72	10.11 ± 0.42
**6g**	>25	11.88 ± 0.60	>25	>25	4.71 ± 0.48
Cisplatin	3.45 ± 0.82	4.57 ± 0.43	1.98 ± 0.15	14.35 ± 0.70	3.19 ± 0.60

^a^ The cytotoxic activity of compounds on the cancer cell lines determined using the CCK8 assay. The data are expressed as the mean ± SD of three independent experiments. IC_50_ values are the concentrations that cause 50% inhibition of cancer cell growth (μM) after 60 h.

**Table 3:** The key targets related to five cancer diseases.

No.	Genes	Uniprot ID	Name
1	AKT1	Q96B36	Proline-rich AKT1 substrate 1
2	CCND1	Q64HP0	G1/S-specific cyclin-D1
3	CDH1	P12830	Cadherin-1
4	EGF	P01133	Proepidermal growth factor
5	EGFR	P00533	Epidermal growth factor receptor
6	ERBB2	P04626	Receptor tyrosine-protein kinase erbB-2
7	GAPDH	P04406	Glyceraldehyde-3-phosphate dehydrogenase
8	HRAS	P01112	GTPase HRas
9	IL6	P05231	Interleukin-6
10	INS	P01308	Insulin
11	KRAS	P01116	GTPase KRas
12	MYC	P01106	Mycproto-oncogene protein
13	PTEN	P60484	Phosphatidylinositol 3,4,5-trisphosphate 3-phosphatase and dual-specificity protein phosphatase PTEN
14	STAT3	P40763	Signal transducer and activator of transcription 3
15	TP53	P04637	Cellular tumor antigen p53
16	VEGFA	P15692	Vascular endothelial growth factor A

## Data Availability

No applicable.

## Supplementary Materials

The list of supplementary materials is as follows. Figure S1: ^1^H NMR spectrum of compound **5a**. Figure S2: ^13^C NMR spectrum of compound **5a**. Figure S3: ^1^H NMR spectrum of compound **5b**. Figure S4: ^13^C NMR spectrum of compound **5b**. Figure S5: ^1^H NMR spectrum of compound **5c**. Figure S6: ^13^C NMR spectrum of compound **5c**. Figure S7: ^1^H NMR spectrum of compound **5d**. Figure S8: ^13^C NMR spectrum of compound **5d**. Figure S9: ^1^H NMR spectrum of compound **5e**. Figure S10: ^13^C NMR spectrum of compound **5e**. Figure S11: ^1^H NMR spectrum of compound **5f**. Figure S12: ^13^C NMR spectrum of compound **5f**. Figure S13: ^1^H NMR spectrum of compound **5g**. Figure S1:. ^13^C NMR spectrum of compound **5g**. Figure S15: ^1^H NMR spectrum of compound **5h**. Figure S16: ^13^C NMR spectrum of compound **5h**. Figure S17: ^1^H NMR spectrum of compound **5i**. Figure S18: ^13^C NMR spectrum of compound **5i**. Figure S19: ^1^H NMR spectrum of compound **5j**. Figure S20: ^13^C NMR spectrum of compound **5j**. Figure S21: ^1^H NMR spectrum of compound **5k**. Figure S22: ^13^C NMR spectrum of compound **5k**. Figure S23: HRMS spectrum of compound **6a**. Figure S24: ^1^H NMR spectrum of compound **6a**. Figure S25: ^13^C NMR spectrum of compound **6a**. Figure S26: HRMS spectrum of compound **6b**. Figure S27: ^1^H NMR spectrum of compound **6b**. Figure S28: ^13^C NMR spectrum of compound **6b**. Figure S29: HRMS spectrum of compound **6c**. Figure S30: ^1^H NMR spectrum of compound **6c**. Figure S31: ^13^C NMR spectrum of compound **6c**. Figure S32: HRMS spectrum of compound **6d**. Figure S33: ^1^H NMR spectrum of compound **6d**. Figure S34: ^13^C NMR spectrum of compound **6d**. Figure S35: HRMS spectrum of compound **6e**. Figure S36: ^1^H NMR spectrum of compound **6e**. Figure S37: ^13^C NMR spectrum of compound **6e**. Figure S38: HRMS spectrum of compound **6f**. Figure S39: ^1^H NMR spectrum of compound **6f**. Figure S40: ^13^C NMR spectrum of compound **6f**. Figure S41: HRMS spectrum of compound **6g**. Figure S42: ^1^H NMR spectrum of compound **6g**. Figure S43: ^13^C NMR spectrum of compound **6g**. Figure S44: HRMS spectrum of compound **6h**. Figure S45: ^1^H NMR spectrum of compound **6h**. Figure S46: ^13^C NMR spectrum of compound **6h**. Figure S47: HRMS spectrum of compound **6i**. Figure S48: ^1^H NMR spectrum of compound **6i**. Figure S49: ^13^C NMR spectrum of compound **6i**. Figure S50: HRMS spectrum of compound **6j**. Figure S51: ^1^H NMR spectrum of compound **6j**. Figure S52: ^13^C NMR spectrum of compound **6j**. Figure S53: HRMS spectrum of compound **6k**. Figure S54: ^1^H NMR spectrum of compound **6k**. Figure S55: ^13^C NMR spectrum of compound **6k**. Figure S56: HRMS spectrum of compound **6l**. Figure S57: ^1^H NMR spectrum of compound **6l**. Figure S58: ^13^C NMR spectrum of compound **6l**. Figure S59.:The total energy of molecular docking between compounds and each target. Table S1: Mean ± SD values of growth inhibition ratios at 2.5 μM drug concentration. Table S2: Mean ± SD values of growth inhibition ratios at 25 μM drug concentration. Table S3: The total energy for the tested compounds **5a**–**5k** against docked proteins binding pockets. Table S4: The total energy for the tested compounds **6a**–**6l** against docked proteins binding pockets. Table S5: The various interactions for the most promising test compounds into the AKT1 binding pockets. Table S6: The various interactions for the most promising test compounds into the EGFR binding pockets. Table S7: The various interactions for the most promising test compounds into the HRAS binding pockets. Table S8: The various interactions for the most promising test compounds into the KRAS binding pockets.

## Sample Availability

No applicible.
