# Supplementary material for: Design, Synthesis, Biological Evaluation and Silico Prediction of Novel Sinomenine Derivatives
Source: Molecules. 2021 Jun 7;26(11):3466. doi: 10.3390/molecules26113466 (PMC8200971; doi:10.3390/molecules26113466)
Supplement: Supplementary file 1 [file molecules-26-03466-s001.zip › molecules-1234591-supplementary.pdf]

Design, Synthesis, Biological Evaluation and Silico Prediction of Novel Sinomenine Derivatives

Shoujie Li[1], Mingjie Gao[2], Xin Nian[3], Liyu Zhang[4], Jinjie Li[5], Dongmei Cui[3,*], Chen Zhang[4,*], Changqi Zhao[1,*]

[1] Gene Engineering and Biotechnology Beijing Key Laboratory, College of Life Science, Beijing Normal University, 19 XinjiekouWai Avenue, Beijing 100875, China; 201831200027@mail.bnu.edu.cn

[2] Research Group Pharmaceutical Bioinformatics, Institute of Pharmaceutical Sciences, Albert-Ludwigs-Universität Freiburg, Freiburg im Breisgau, 79106, Germany; mingjie.gao@pharmazie.uni-freiburg.de

[3] College of Pharmaceutical Science, Zhejiang University of Technology, 18 Chaowang Road, Hangzhou 310014, China; zjdqnx@126.com

[4] College of Pharmaceutical Sciences, Zhejiang University, 866 Yuhangtang Road, Hangzhou 310058, China; liyuzhang@zju.edu.cn

[5] Beijing Key Laboratory of Bioactive Substances and Functional Foods, Beijing Union University, 197 Beitucheng West Road, Beijing 100191, China; lijinjie.7785004@163.com

* Correspondence: cuidongmei@zjut.edu.cn (D.C.); chenzhang@zju.edu.cn (C.Z.); 04020@bnu.edu.cn (C.Z.); Tel: +86-010-58805046 (Changqi Zhao)

This Word file includes:

Figures S1 to S59

Tables S1 to S8.

Table of Contents

Figure S1. ^{1}H NMR spectrum of compound **5a**.
Figure S2. ^{13}C NMR spectrum of compound **5a**.
Figure S3. ^{1}H NMR spectrum of compound **5b**.
Figure S4. ^{13}C NMR spectrum of compound **5b**.
Figure S5. ^{1}H NMR spectrum of compound **5c**.
Figure S6. ^{13}C NMR spectrum of compound **5c**.
Figure S7. ^{1}H NMR spectrum of compound **5d**.
Figure S8. ^{13}C NMR spectrum of compound **5d**.
Figure S9. ^{1}H NMR spectrum of compound **5e**.
Figure S10. ^{13}C NMR spectrum of compound **5e**.
Figure S11. ^{1}H NMR spectrum of compound **5f**.
Figure S12. ^{13}C NMR spectrum of compound **5f**.
Figure S13. ^{1}H NMR spectrum of compound **5g**.
Figure S14. ^{13}C NMR spectrum of compound **5g**.
Figure S15. ^{1}H NMR spectrum of compound **5h**.
Figure S16. ^{13}C NMR spectrum of compound **5h**.
Figure S17. ^{1}H NMR spectrum of compound **5i**.
Figure S18. ^{13}C NMR spectrum of compound **5i**.
Figure S19. ^{1}H NMR spectrum of compound **5j**.
Figure S20. ^{13}C NMR spectrum of compound **5j**.
Figure S21. ^{1}H NMR spectrum of compound **5k**.
Figure S22. ^{13}C NMR spectrum of compound **5k**.
Figure S23. HRMS spectrum of compound **6a**.
Figure S24. ^{1}H NMR spectrum of compound **6a**.
Figure S25. ^{13}C NMR spectrum of compound **6a**.
Figure S26. HRMS spectrum of compound **6b**.
Figure S27. ^{1}H NMR spectrum of compound **6b**.
Figure S28. ^{13}C NMR spectrum of compound **6b**.
Figure S29. HRMS spectrum of compound **6c**.
Figure S30. ^{1}H NMR spectrum of compound **6c**.
Figure S31. ^{13}C NMR spectrum of compound **6c**.
Figure S32. HRMS spectrum of compound **6d**.
Figure S33. ^{1}H NMR spectrum of compound **6d**.
Figure S34. ^{13}C NMR spectrum of compound **6d**.
Figure S35. HRMS spectrum of compound **6e**.
Figure S36. ^{1}H NMR spectrum of compound **6e**.
Figure S37. ^{13}C NMR spectrum of compound **6e**.
Figure S38. HRMS spectrum of compound **6f**.
Figure S39. ^{1}H NMR spectrum of compound **6f**.
Figure S40. ^{13}C NMR spectrum of compound **6f**.
Figure S41. HRMS spectrum of compound **6g**.
Figure S42. ^{1}H NMR spectrum of compound **6g**.
Figure S43. ^{13}C NMR spectrum of compound **6g**.
Figure S44. HRMS spectrum of compound **6h**.
Figure S45. ^{1}H NMR spectrum of compound **6h**.
Figure S46. ^{13}C NMR spectrum of compound **6h**.
Figure S47. HRMS spectrum of compound **6i**.
Figure S48. ^{1}H NMR spectrum of compound **6i**.
Figure S49. ^{13}C NMR spectrum of compound **6i**.
Figure S50. HRMS spectrum of compound **6j**.
Figure S51. ^{1}H NMR spectrum of compound **6j**.
Figure S52. ^{13}C NMR spectrum of compound **6j**.
Figure S53. HRMS spectrum of compound **6k**.
Figure S54. ^{1}H NMR spectrum of compound **6k**.
Figure S55. ^{13}C NMR spectrum of compound **6k**.
Figure S56. HRMS spectrum of compound **6l**.
Figure S57. ^{1}H NMR spectrum of compound **6l**.
Figure S58. ^{13}C NMR spectrum of compound **6l**.
Figure S59. The total energy of molecular docking between compounds and each target.
Table S1. Mean ± SD values of growth inhibition ratios at 2.5 μm drug concentration.
Table S2. Mean ± SD values of growth inhibition ratios at 25 μm drug concentration.
Table S3. The total energy for the tested compounds **5a-5k** against docked proteins binding pockets.
Table S4. The total energy for the tested compounds **6a-6l** against docked proteins binding pockets.
Table S5. The various interactions for the most promising test compounds into the AKT1 binding pockets.
Table S6. The various interactions for the most promising test compounds into the EGFR binding pockets.
Table S7. The various interactions for the most promising test compounds into the HRAS binding pockets.
Table S8. The various interactions for the most promising test compounds into the KRAS binding pockets.

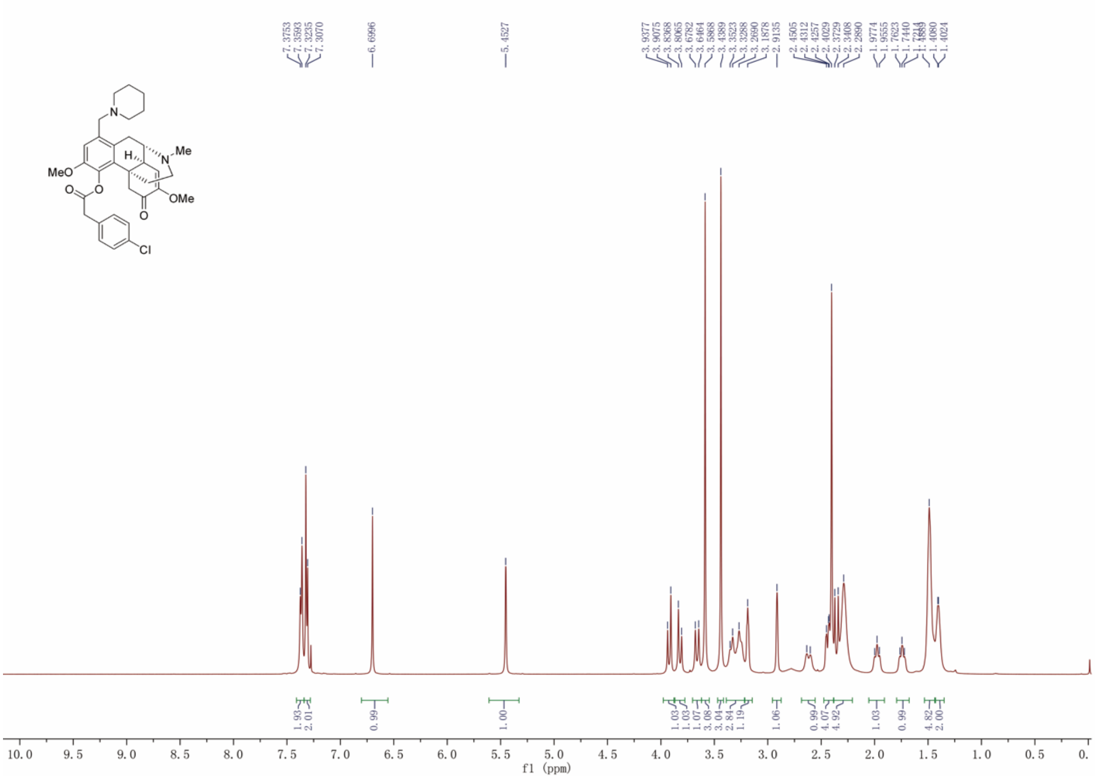

Figure S1. ^{1}H NMR spectrum of compound **5a**.

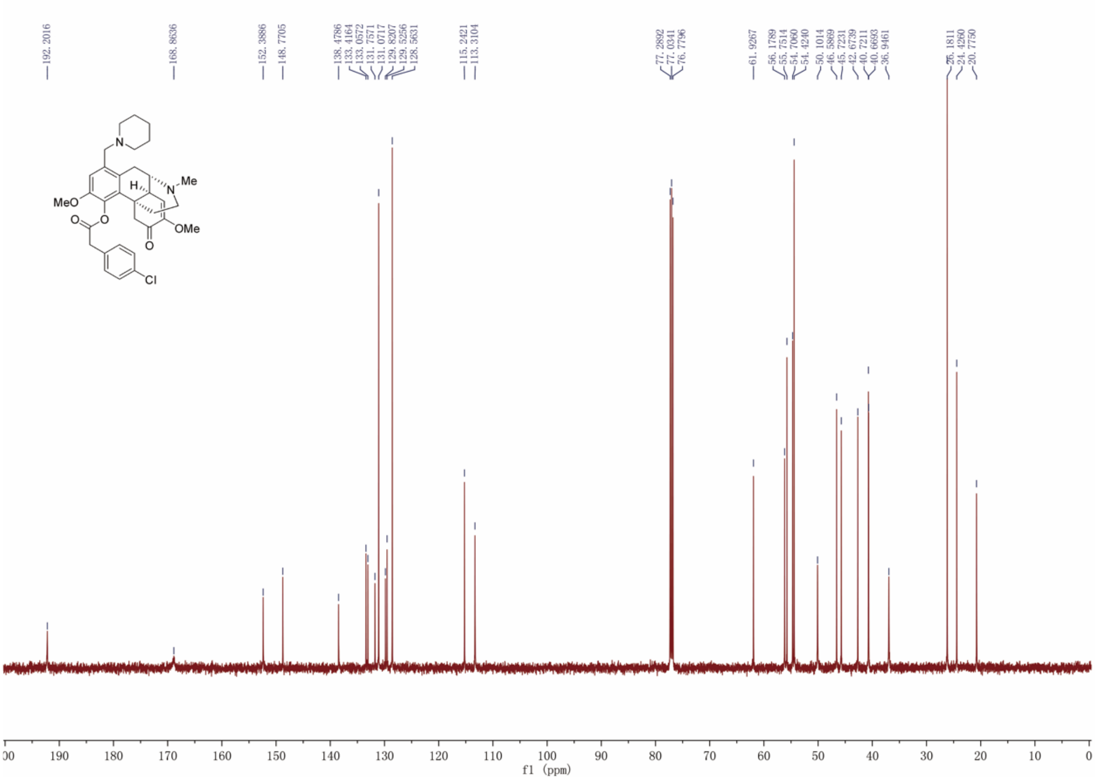

Figure S2. ^{13}C NMR spectrum of compound **5a**.

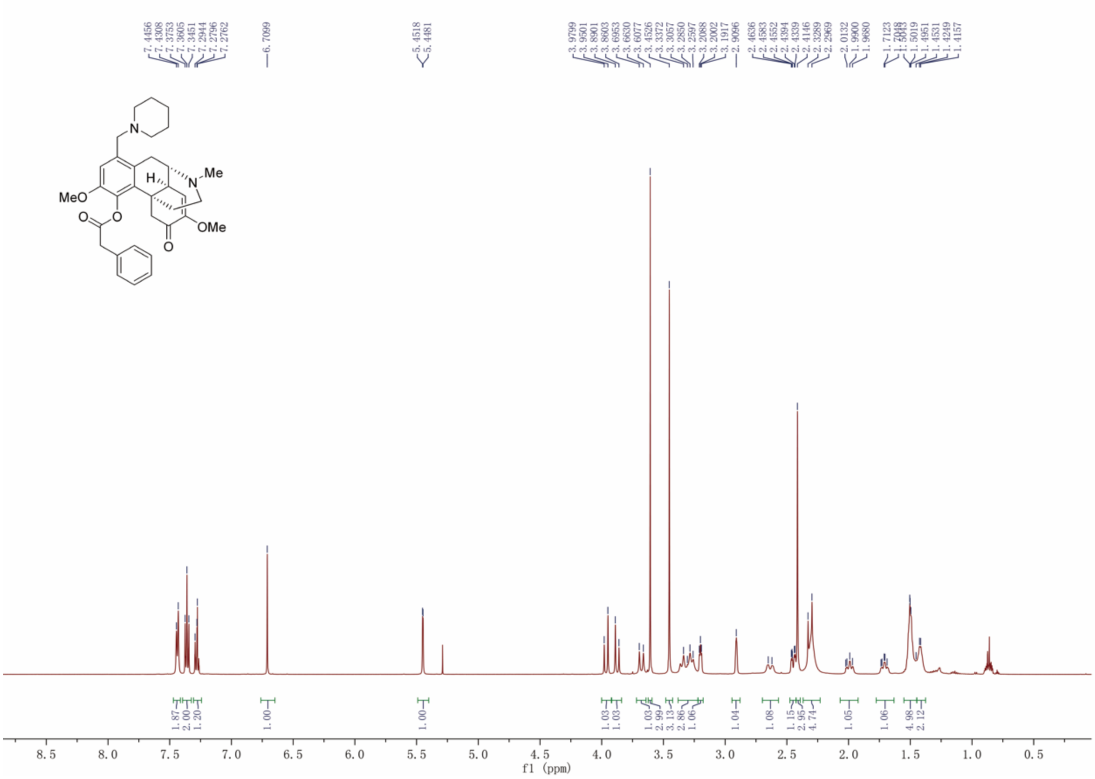

Figure S3. ^{1}H NMR spectrum of compound **5b**.

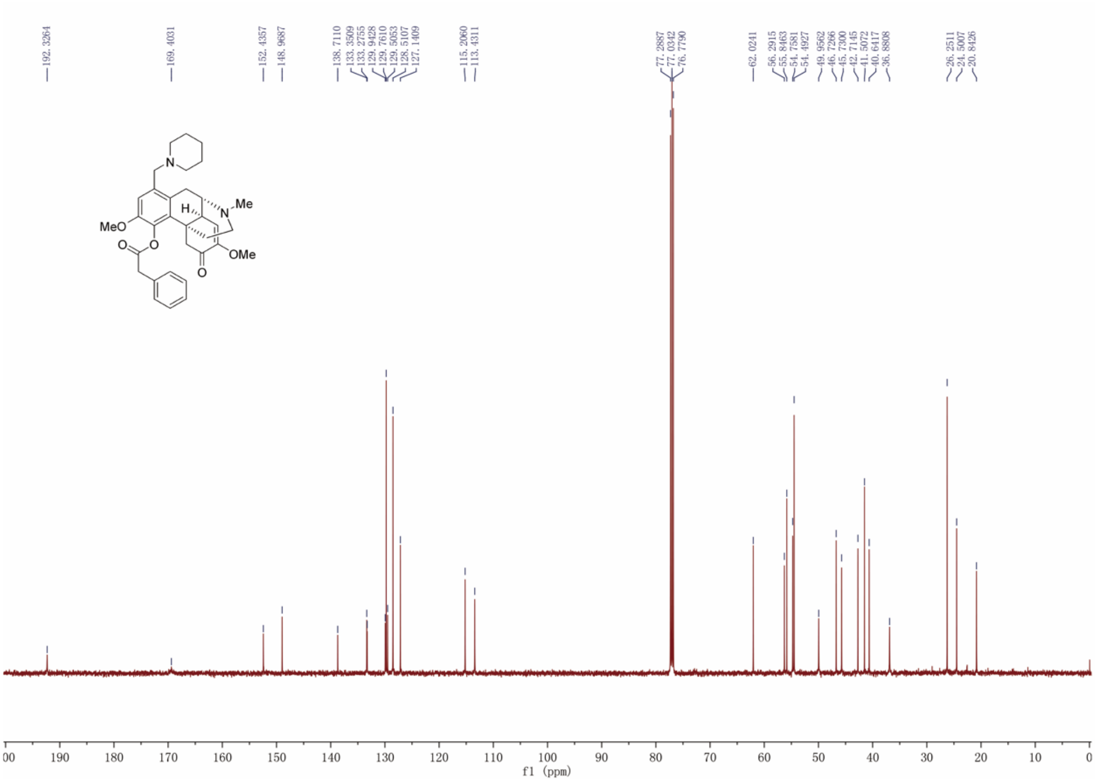

Figure S4. ^{13}C NMR spectrum of compound **5b**.

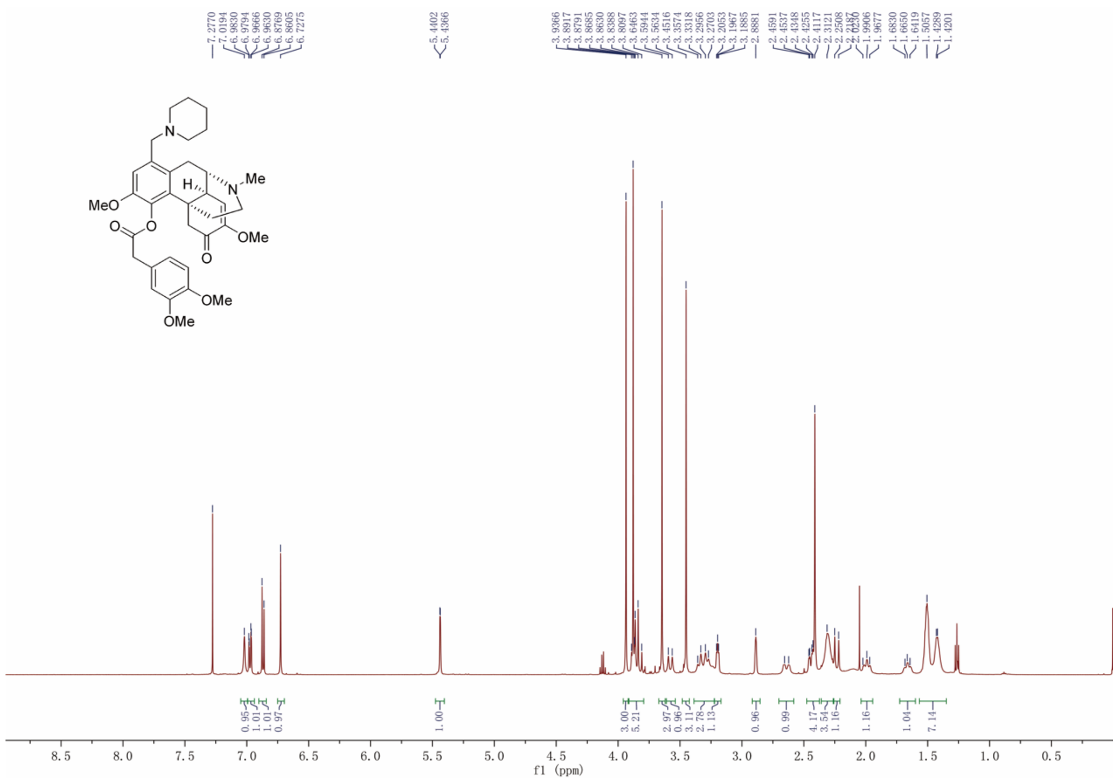

Figure S5. ^{1}H NMR spectrum of compound **5c**.

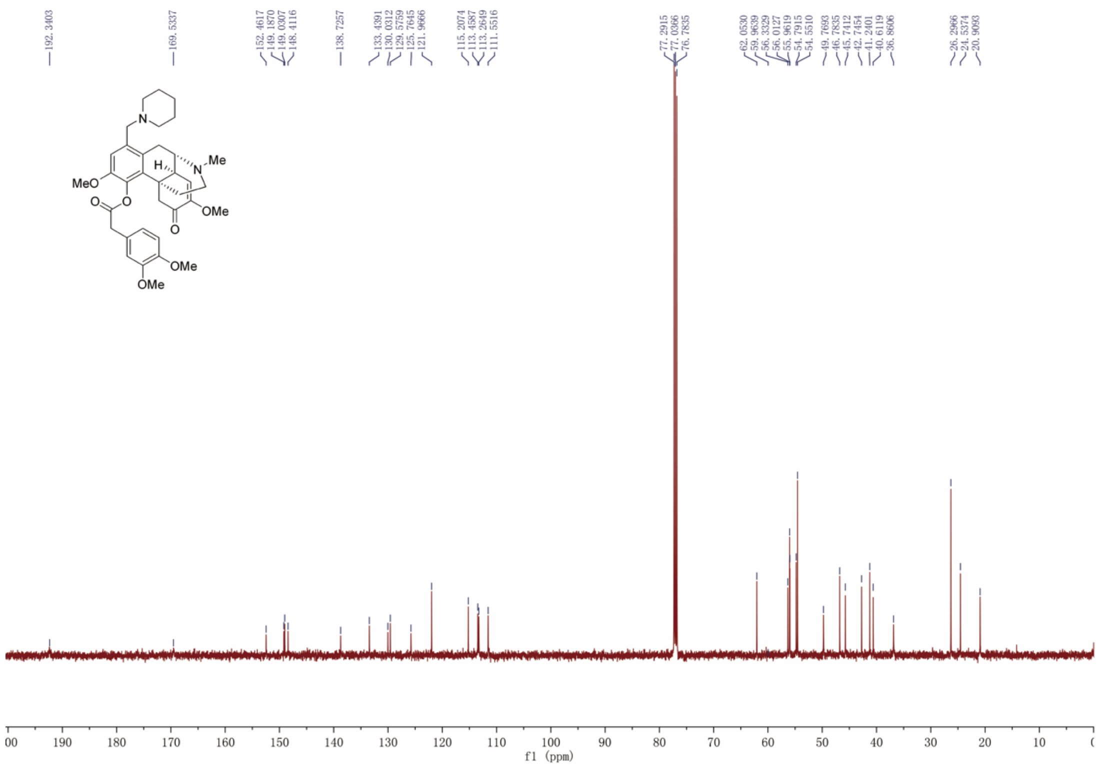

Figure S6. ^{13}C NMR spectrum of compound **5c**.

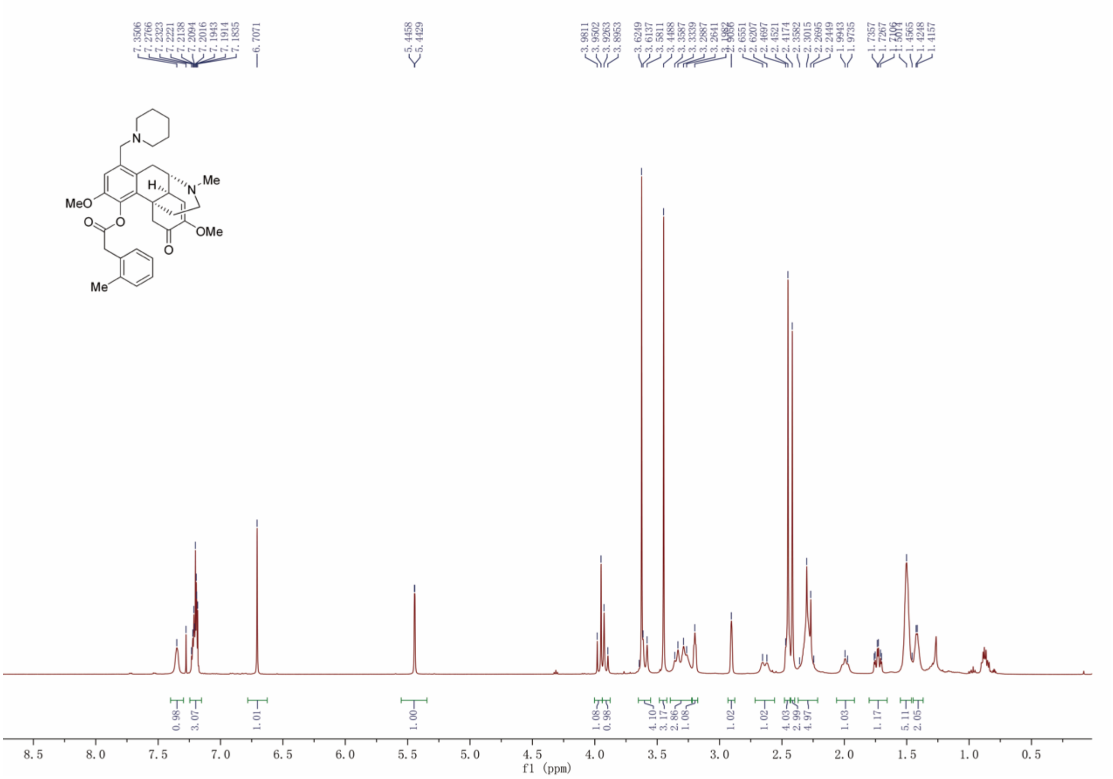

Figure S7. ^{1}H NMR spectrum of compound **5d**.

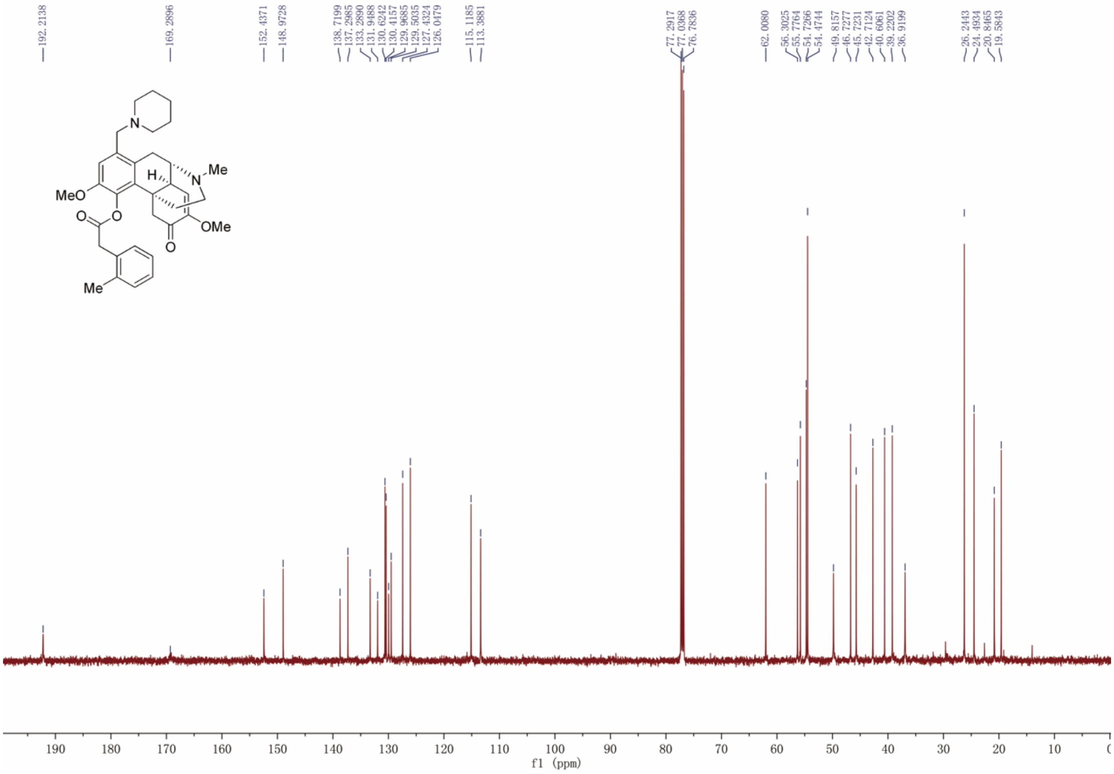

Figure S8. ^{13}C NMR spectrum of compound **5d**.

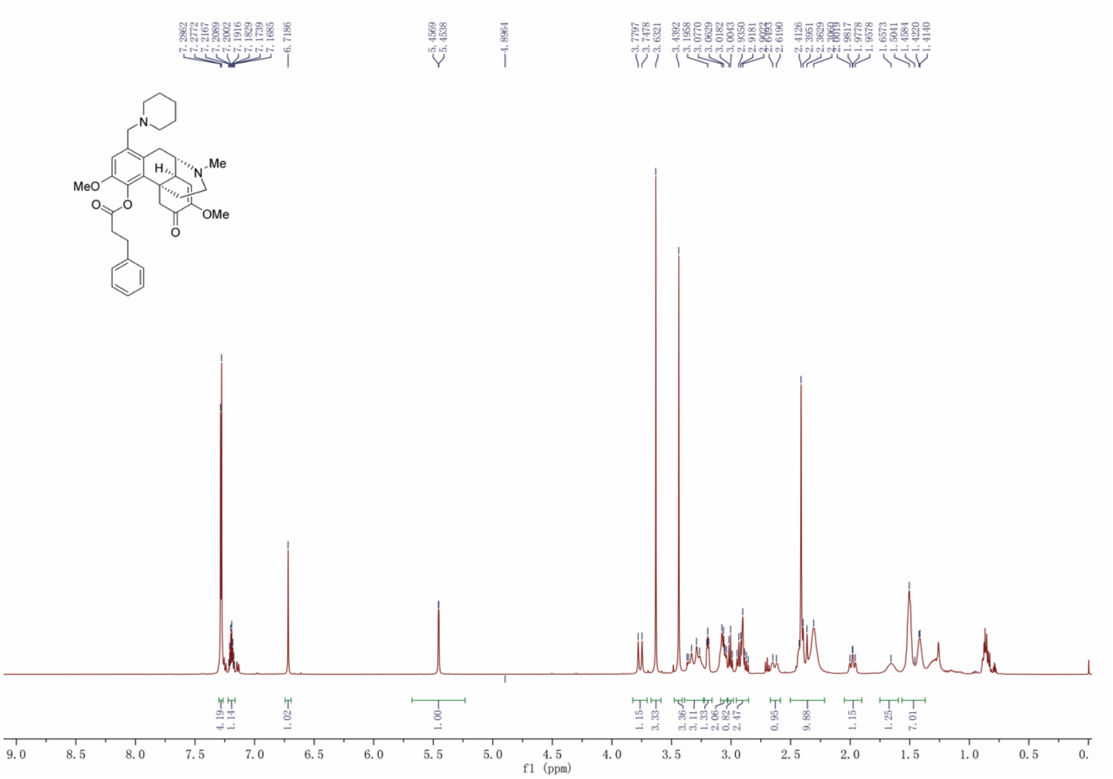

Figure S9. ¹H NMR spectrum of compound **5e**.

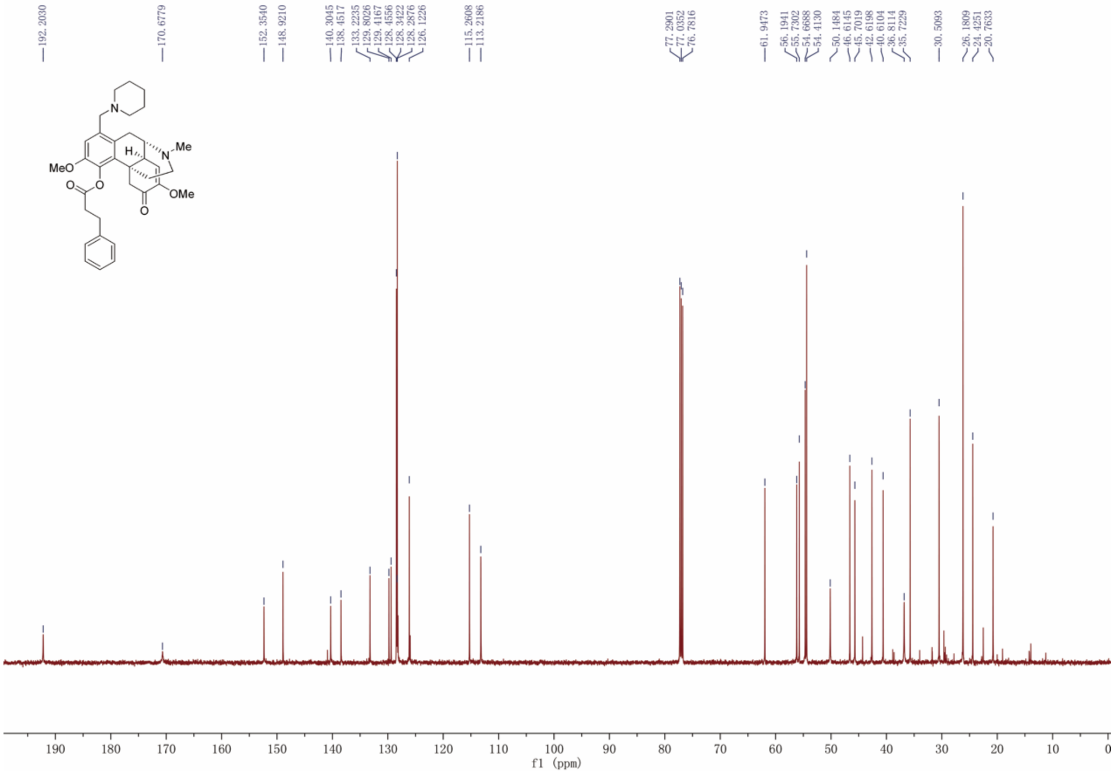

Figure S10. ¹³C NMR spectrum of compound **5e**.

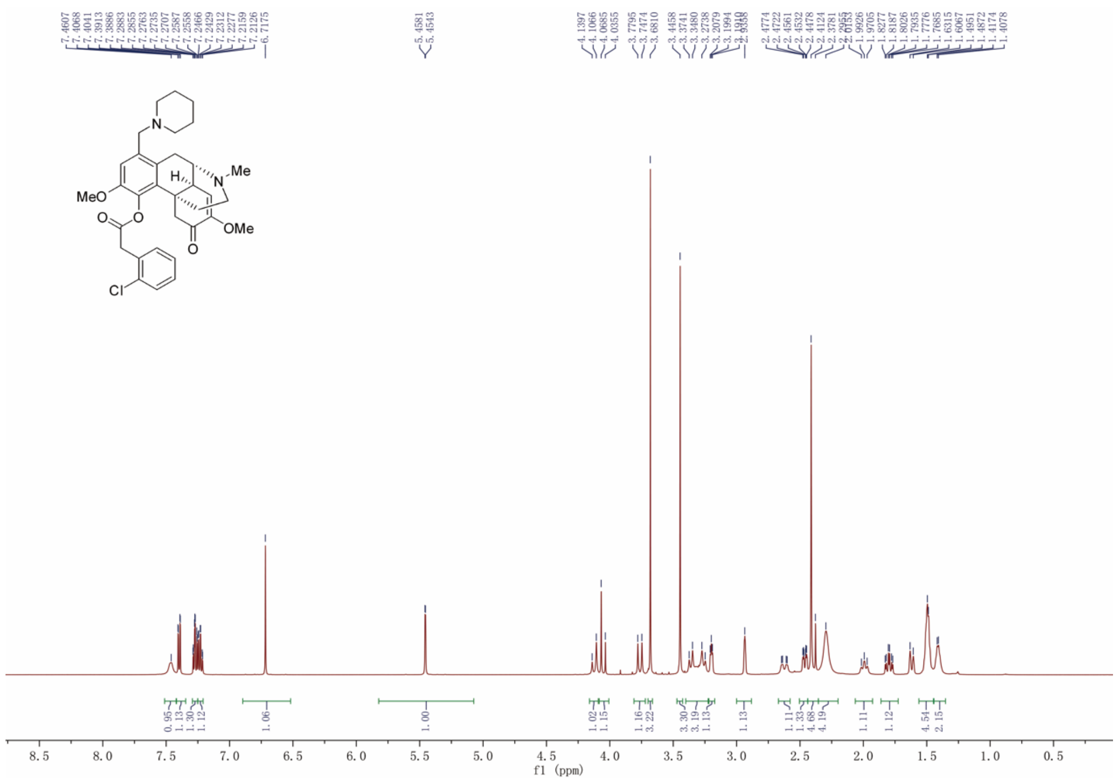

Figure S11. ^{1}H NMR spectrum of compound **5f**.

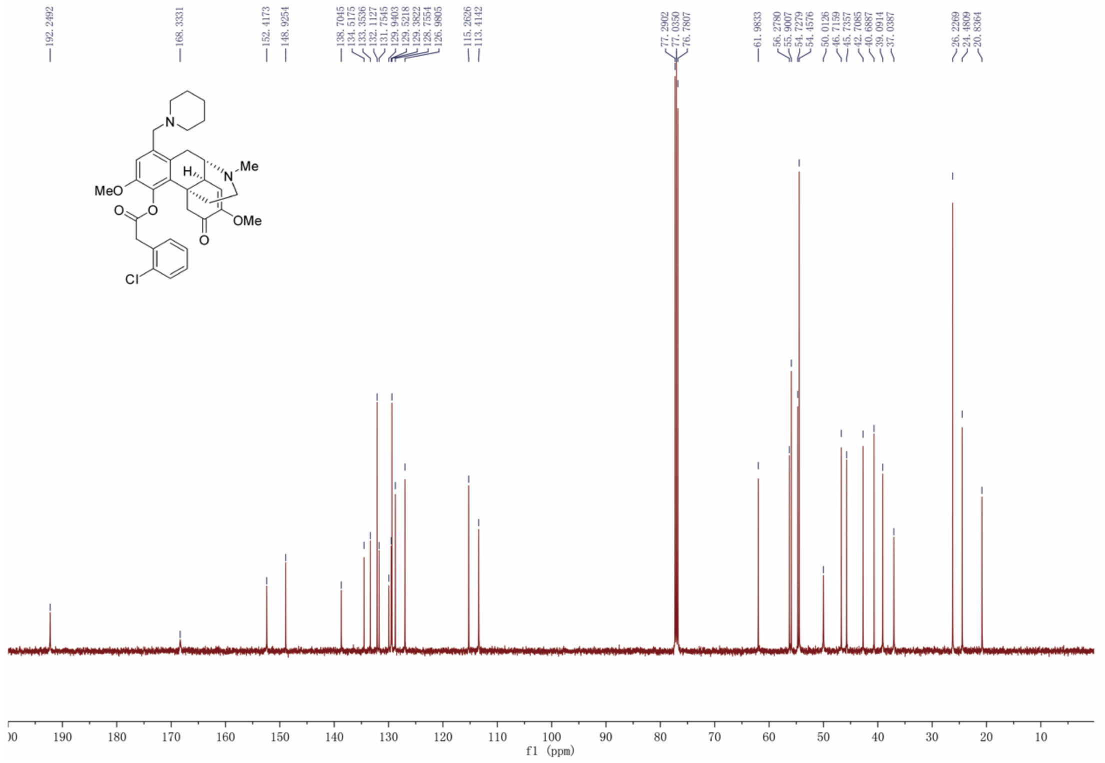

Figure S12. ^{13}C NMR spectrum of compound **5f**.

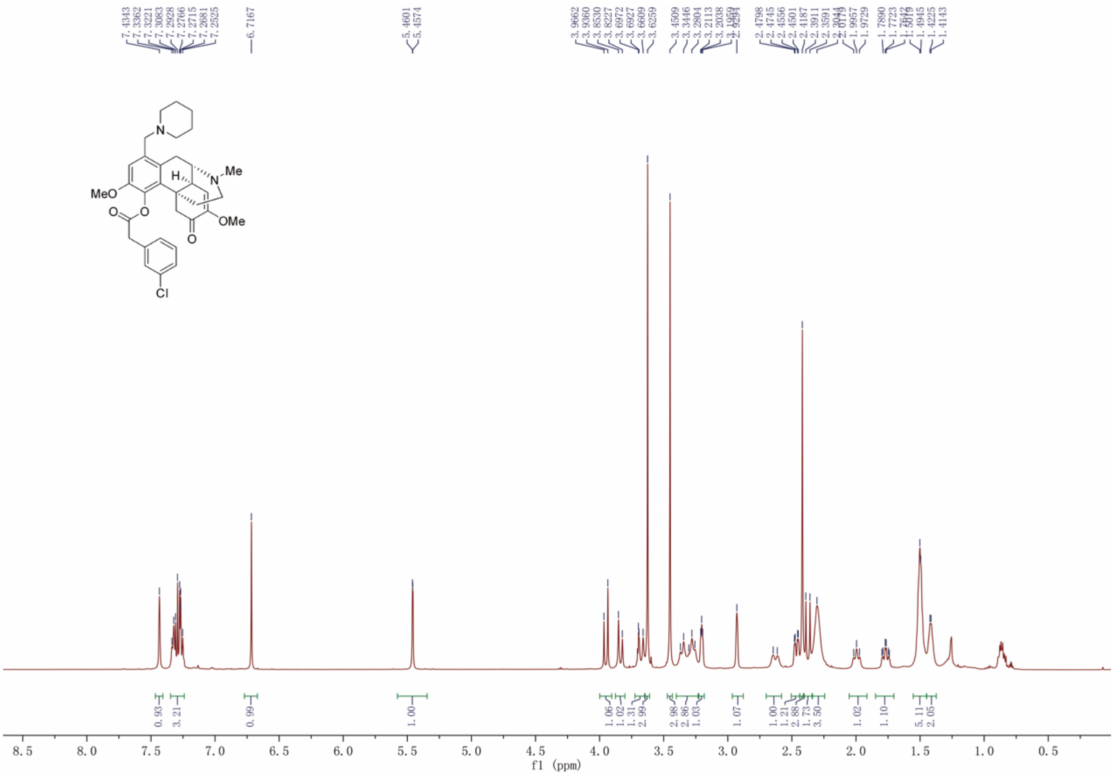

Figure S13. ^{1}H NMR spectrum of compound **5g**.

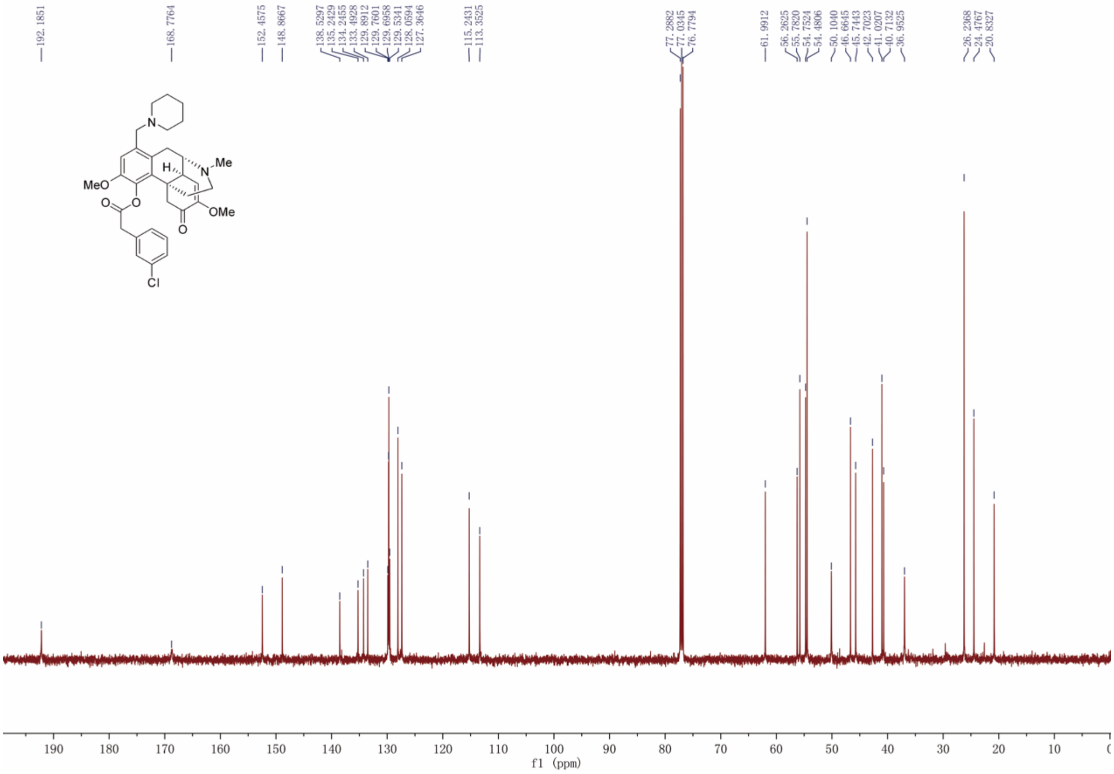

Figure S14. ^{13}C NMR spectrum of compound **5g**.

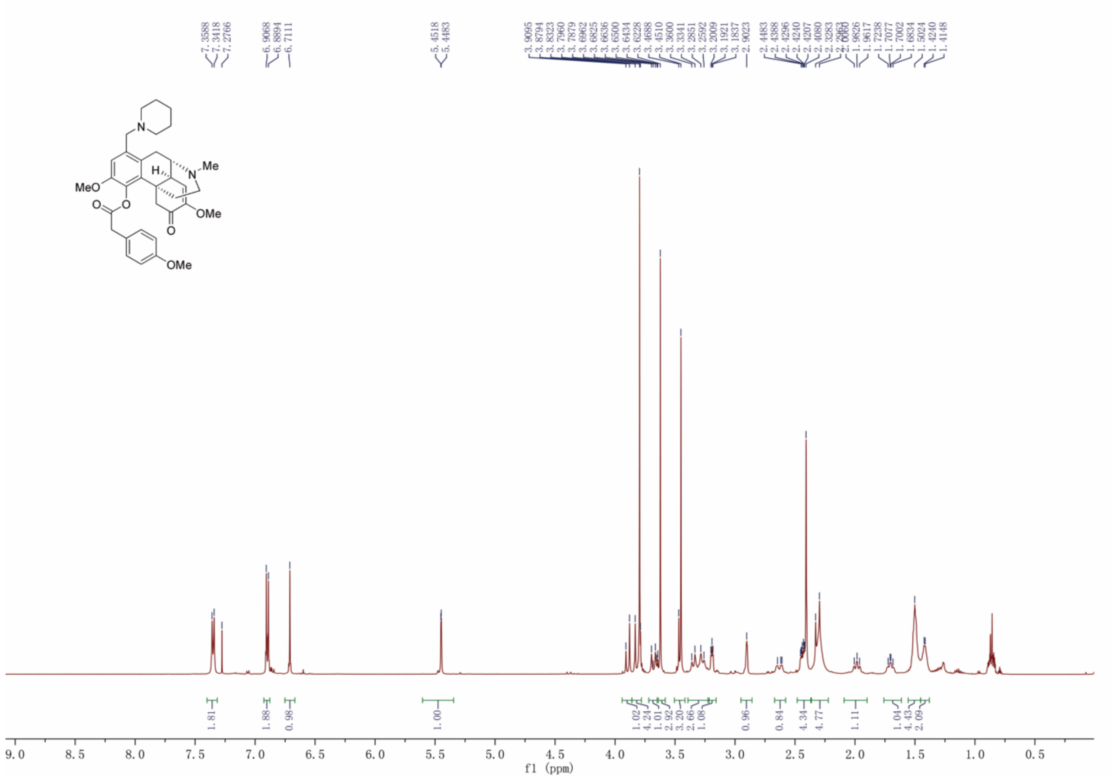

Figure S15. ^{1}H NMR spectrum of compound **5h**.

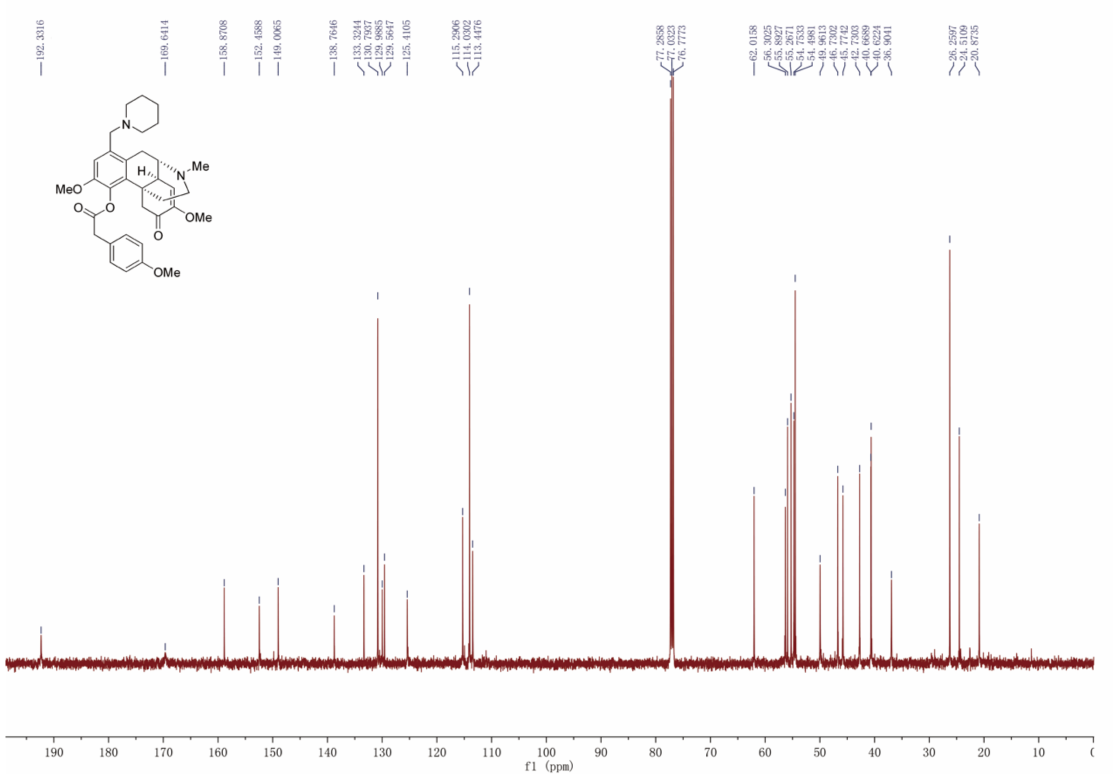

Figure S16. ^{13}C NMR spectrum of compound **5h**.

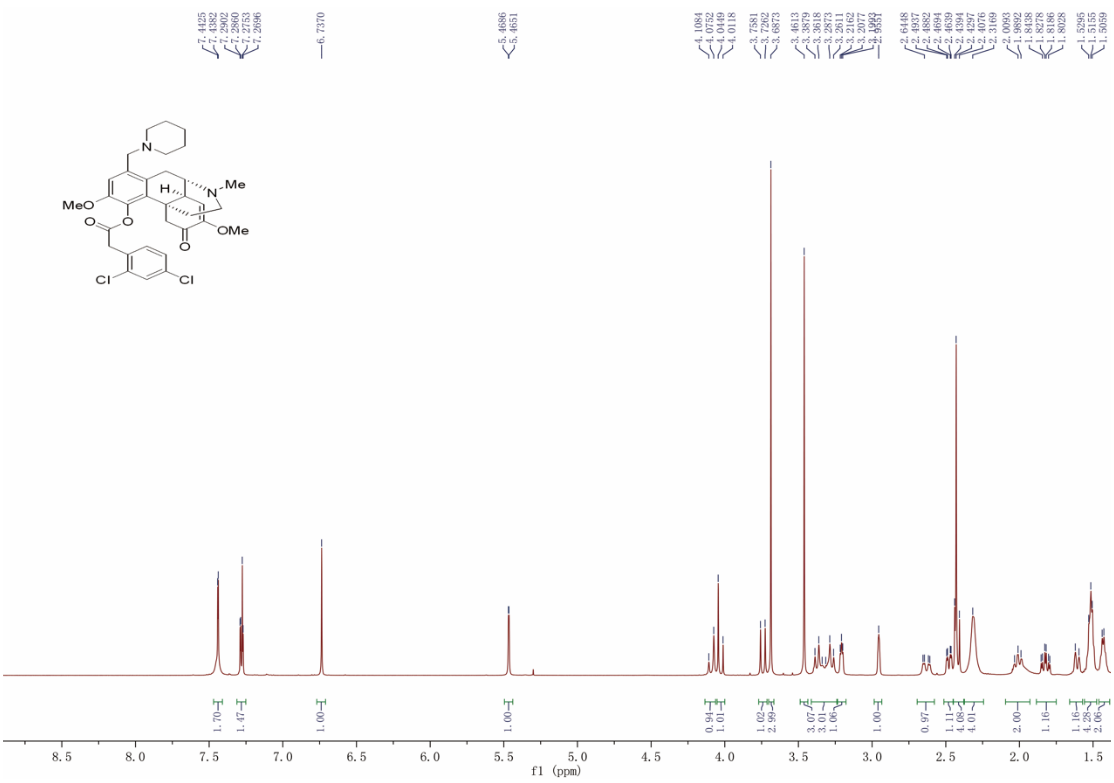

Figure S17. ^{1}H NMR spectrum of compound **5i**.

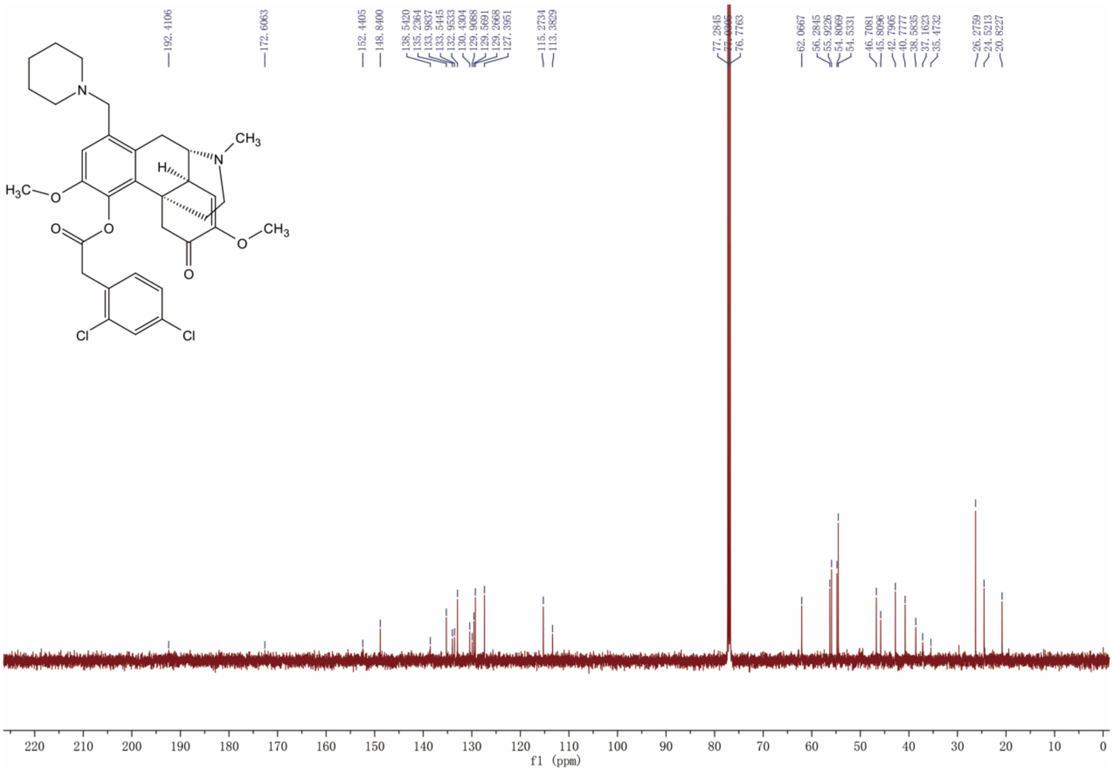

Figure S18. ^{13}C NMR spectrum of compound **5i**.

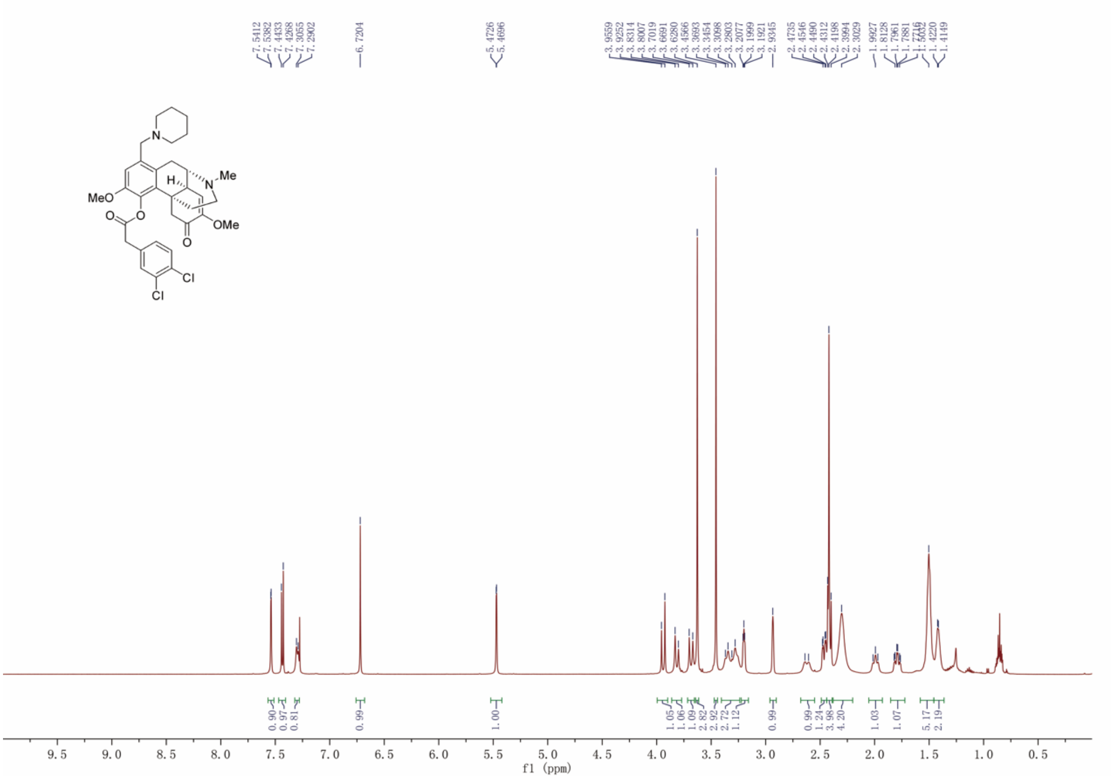

Figure S19. ^{1}H NMR spectrum of compound **5j**.

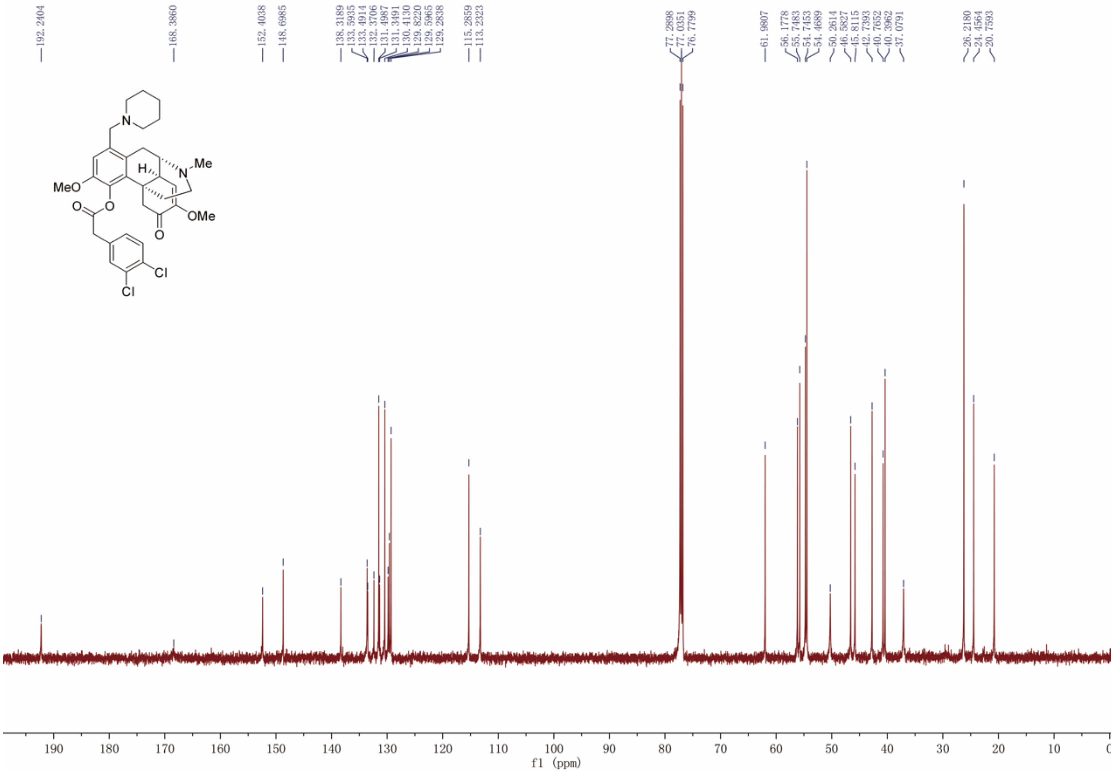

Figure S20. ^{13}C NMR spectrum of compound **5j**.

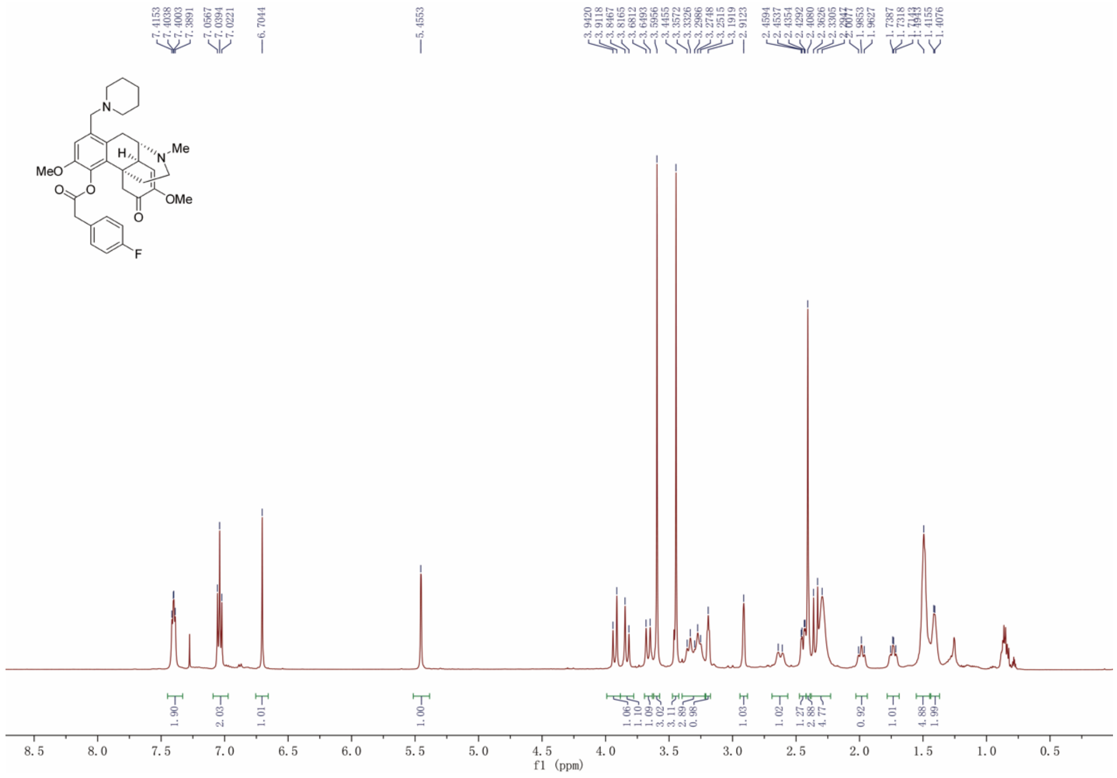

Figure S21. ^{1}H NMR spectrum of compound **5k**.

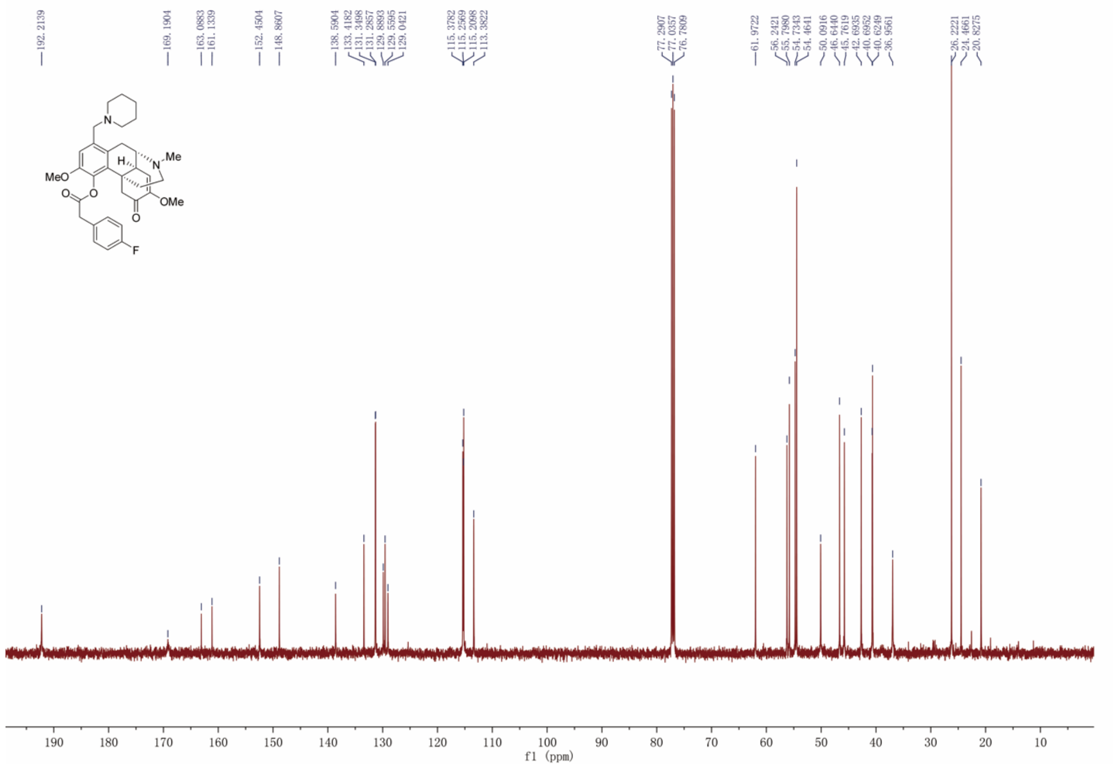

Figure S22. ^{13}C NMR spectrum of compound **5k**.

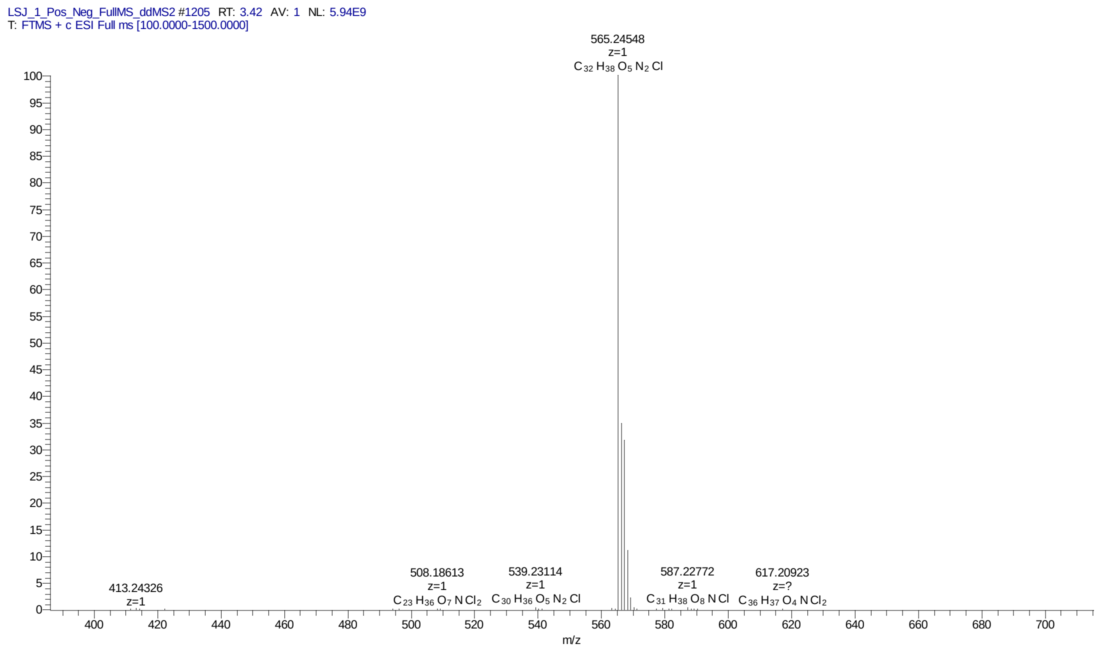

Figure S23. HRMS spectrum of compound **6a**.

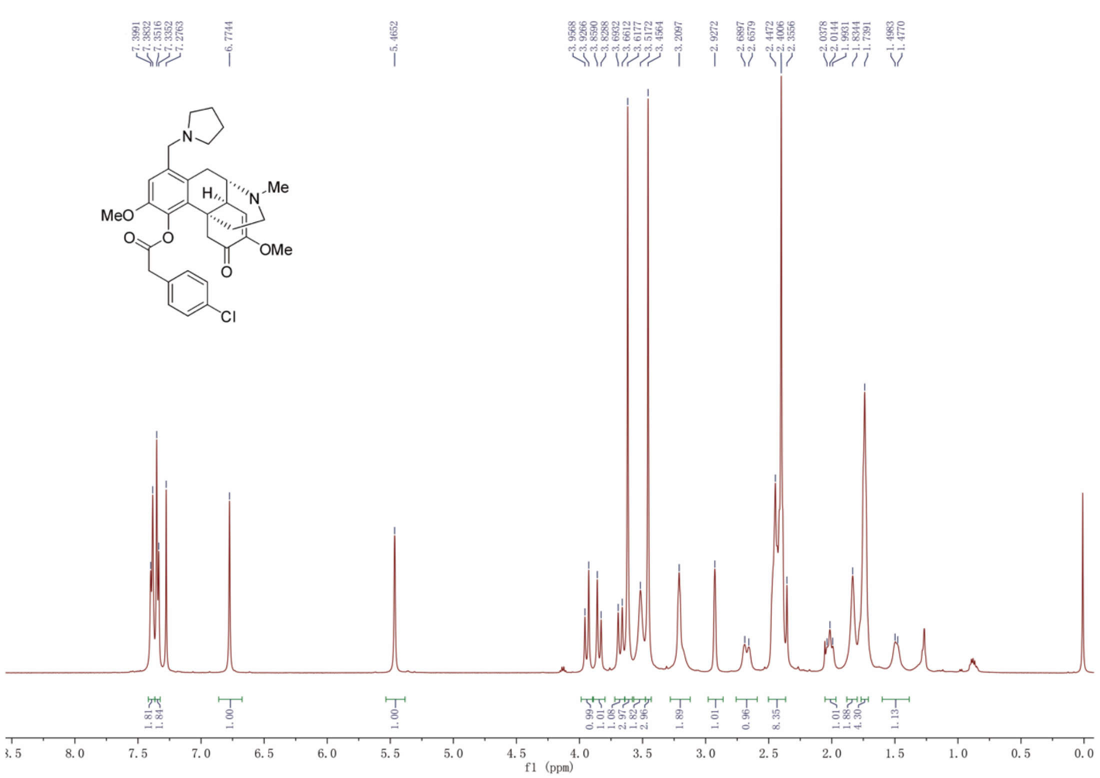

Figure S24. ^{1}H NMR spectrum of compound **6a**.

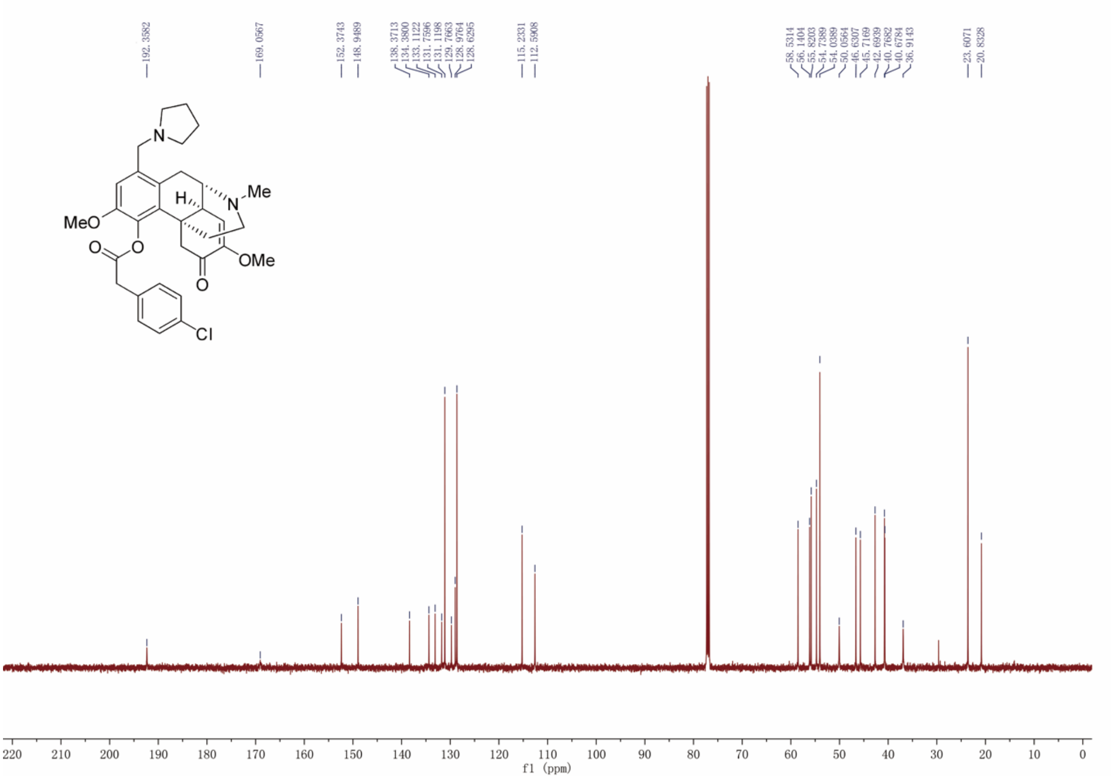

Figure S25. ^{13}C NMR spectrum of compound **6a**.

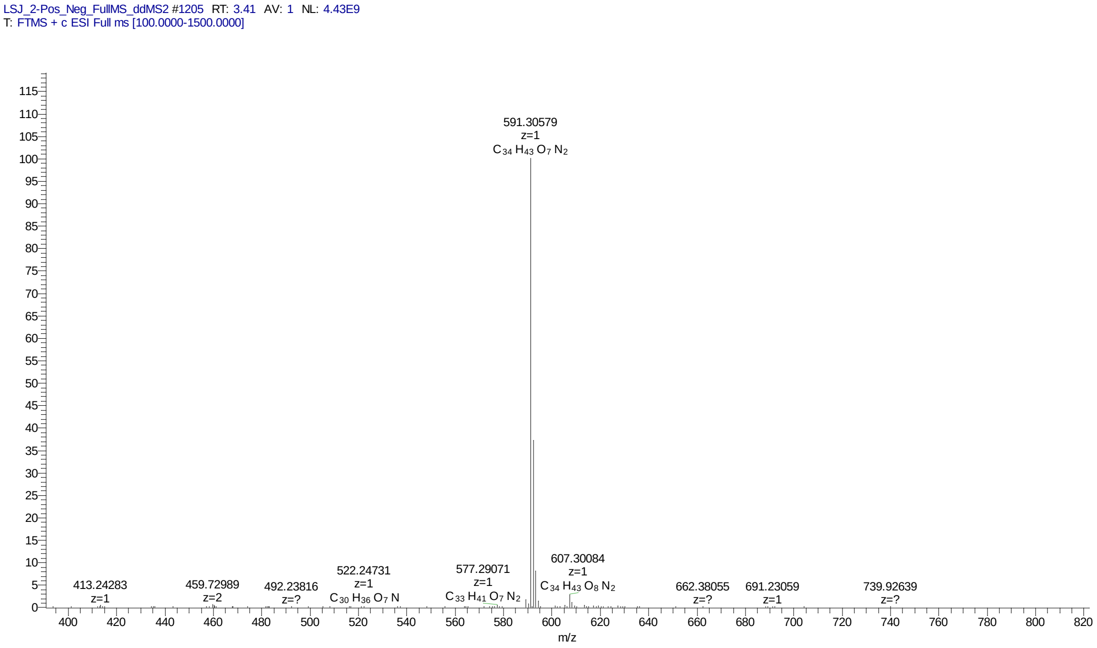

Figure S26. HRMS spectrum of compound **6b**.

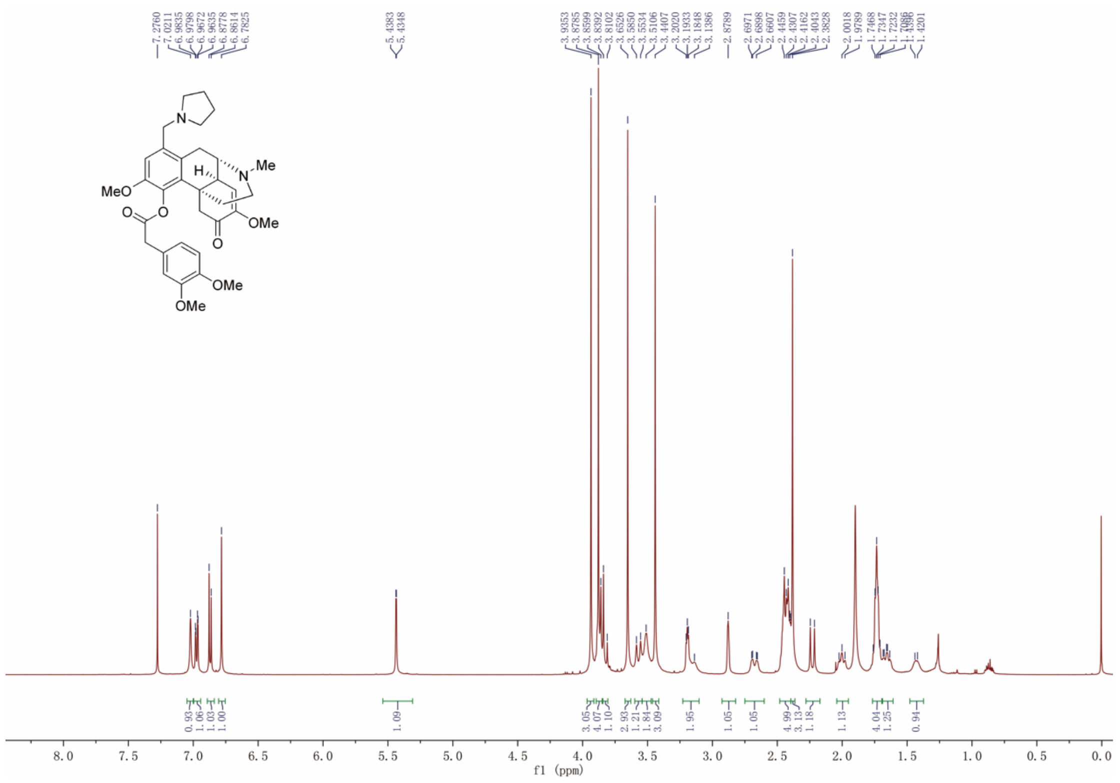

Figure S27. ^{1}H NMR spectrum of compound **6b**.

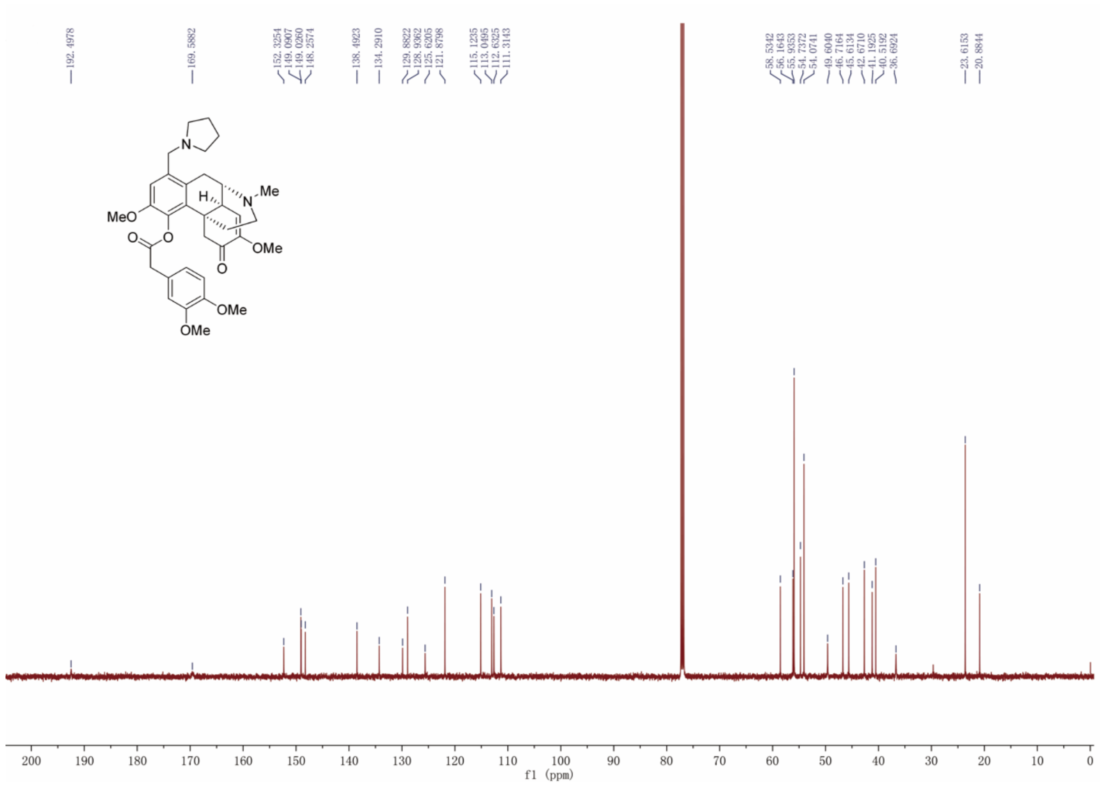

Figure S28. ^{13}C NMR spectrum of compound **6b**.

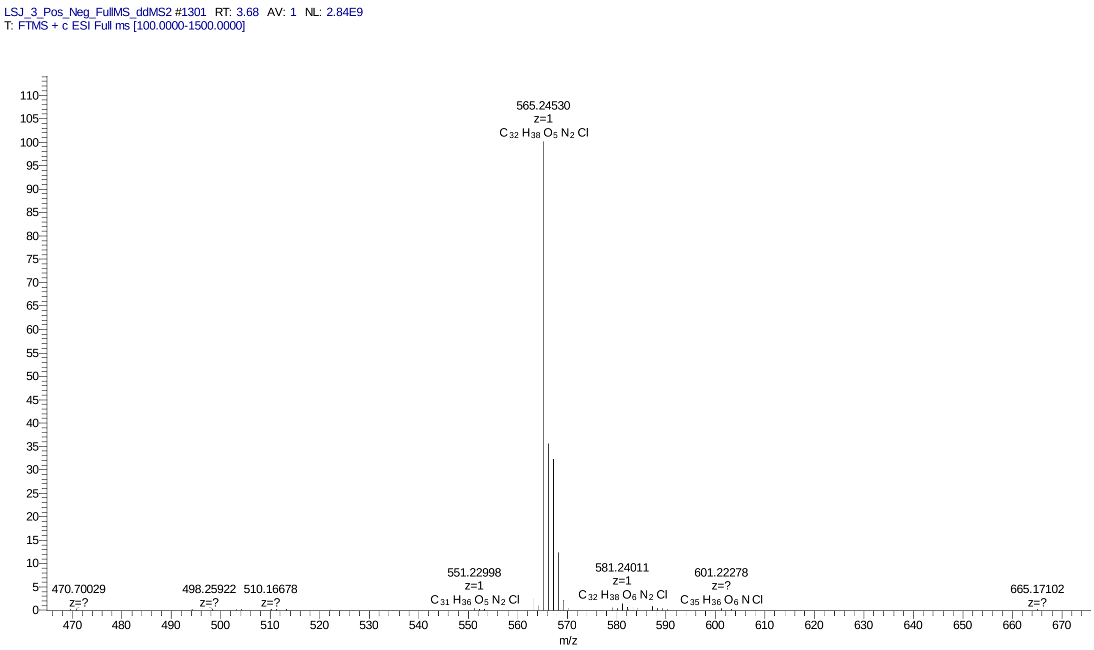

Figure S29. HRMS spectrum of compound **6c**.

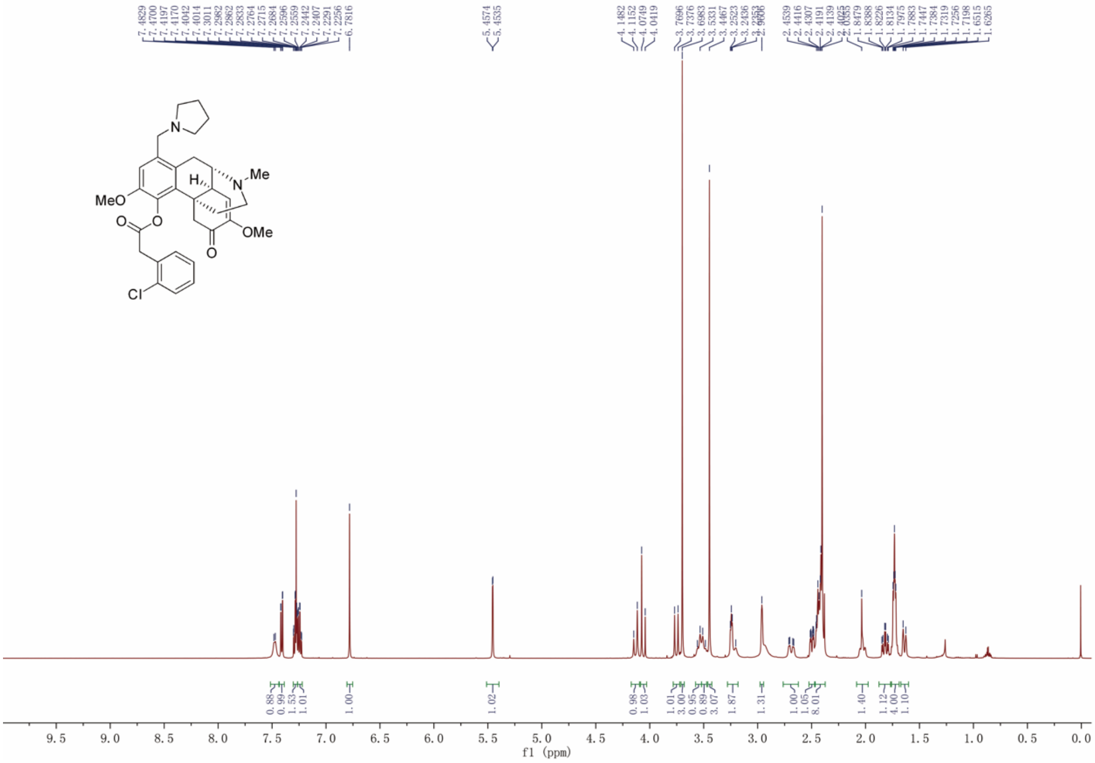

Figure S30. ^{1}H NMR spectrum of compound **6c**.

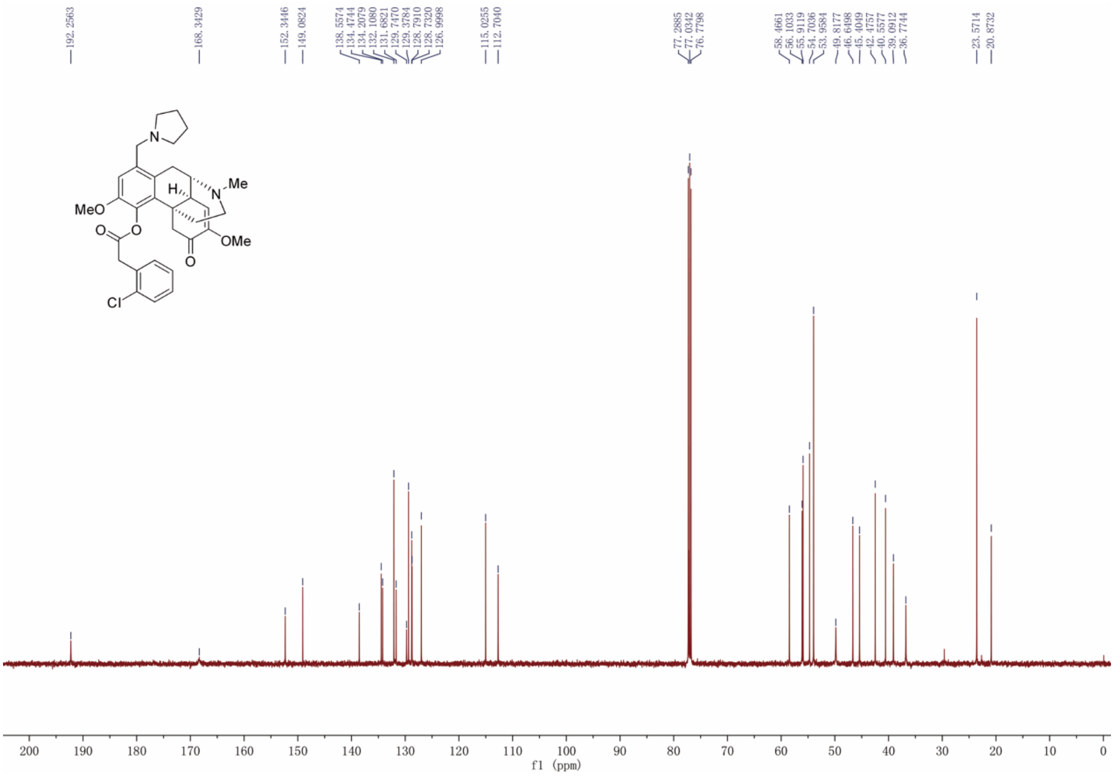

Figure S31. ^{13}C NMR spectrum of compound **6c**.

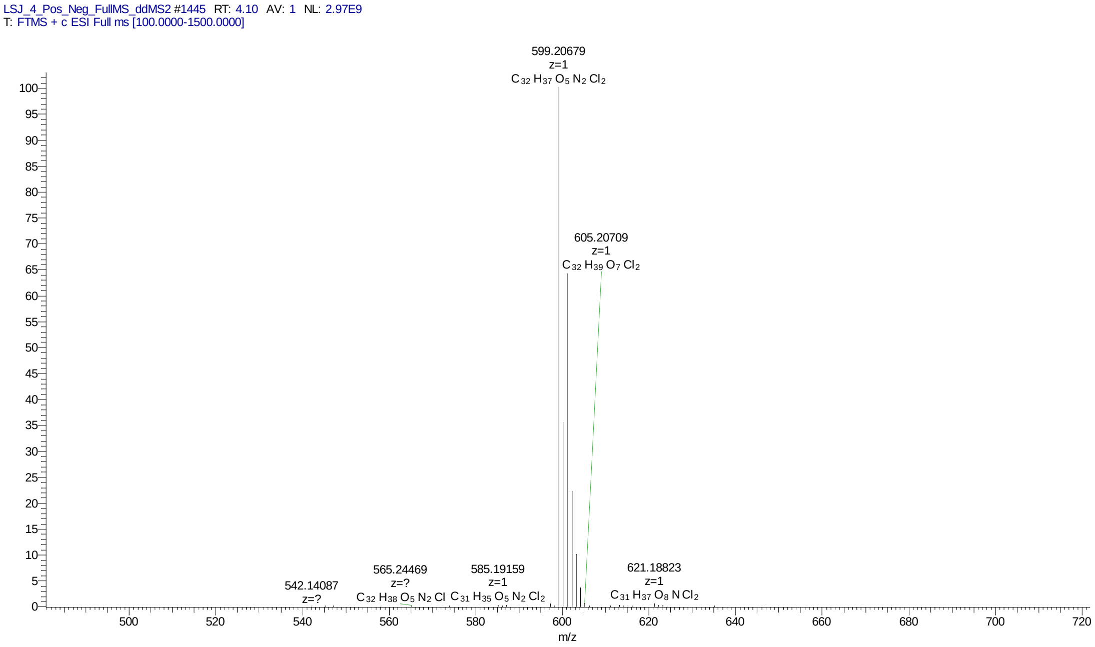

Figure S32. HRMS spectrum of compound **6d**.

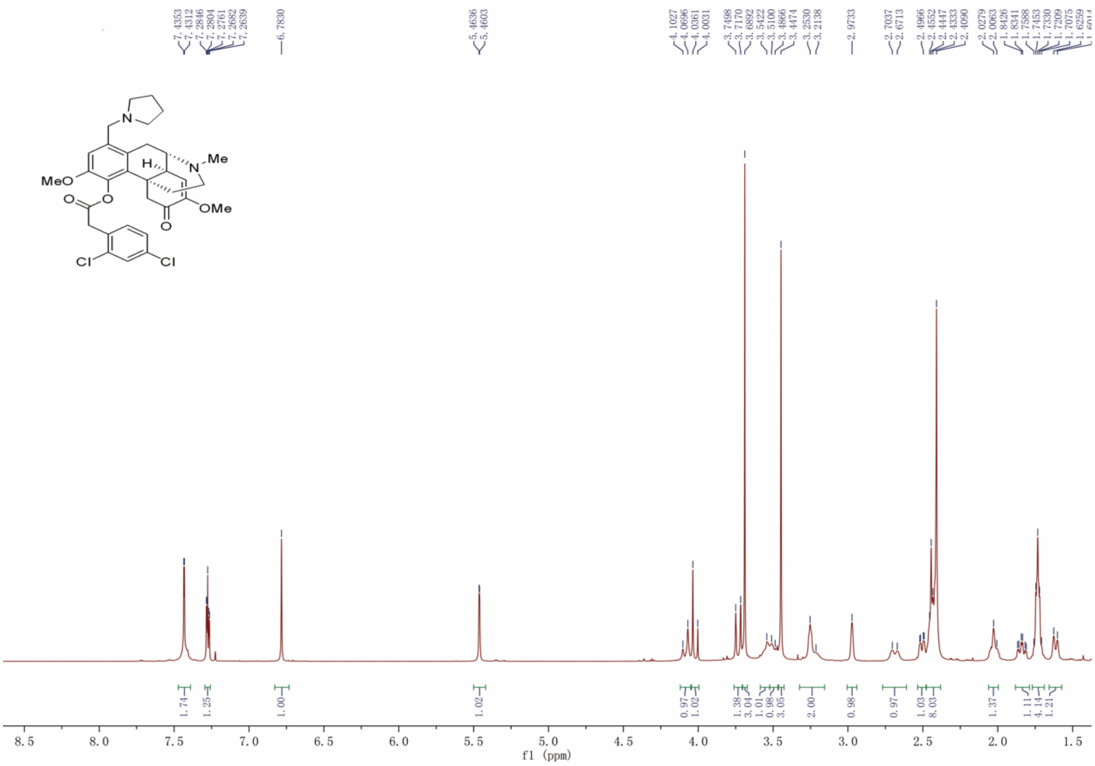

Figure S33. ^{1}H NMR spectrum of compound **6d**.

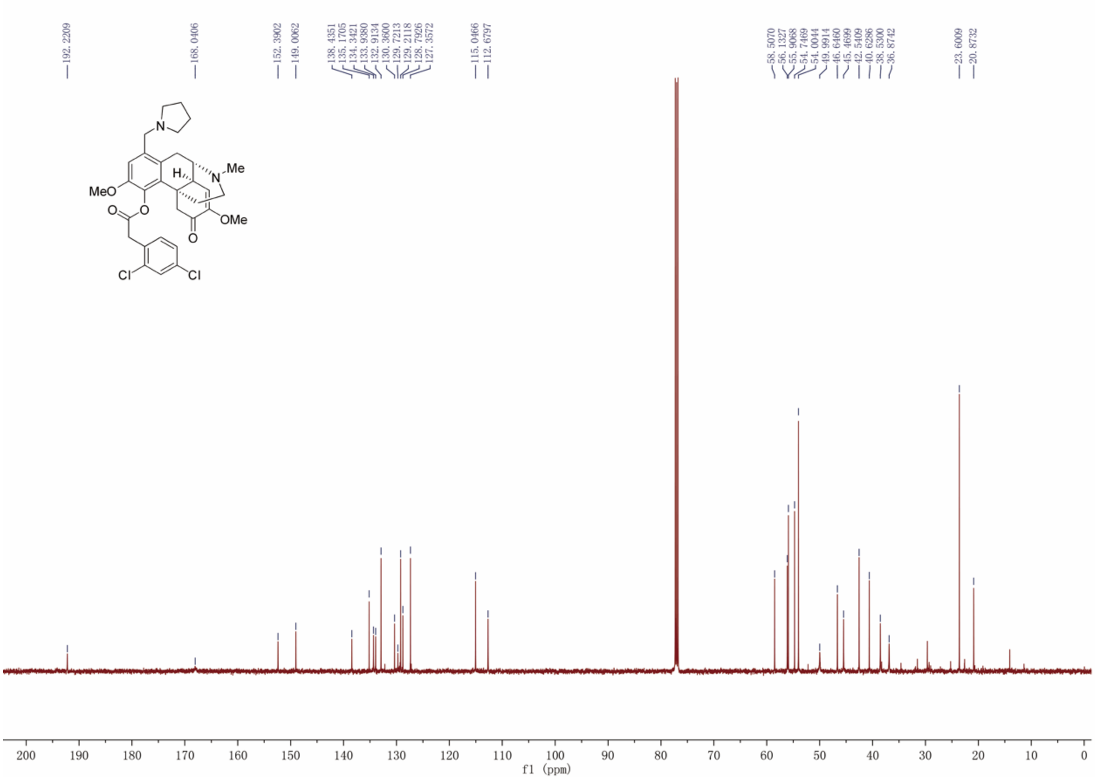

Figure S34. ^{13}C NMR spectrum of compound **6d**.

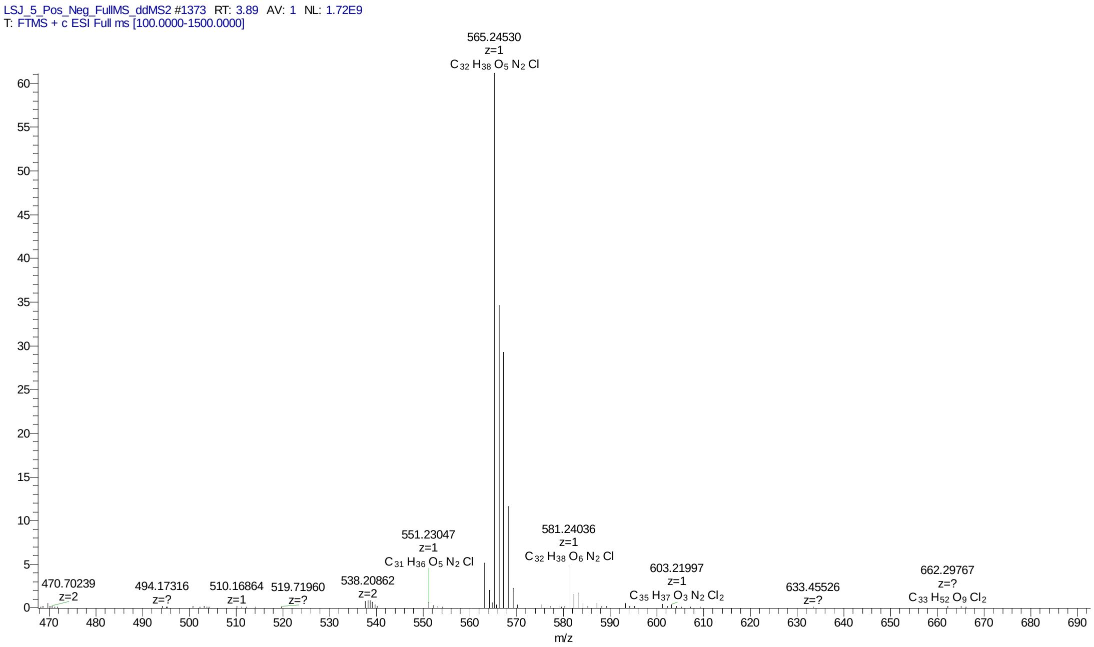

Figure S35. HRMS spectrum of compound **6e**.

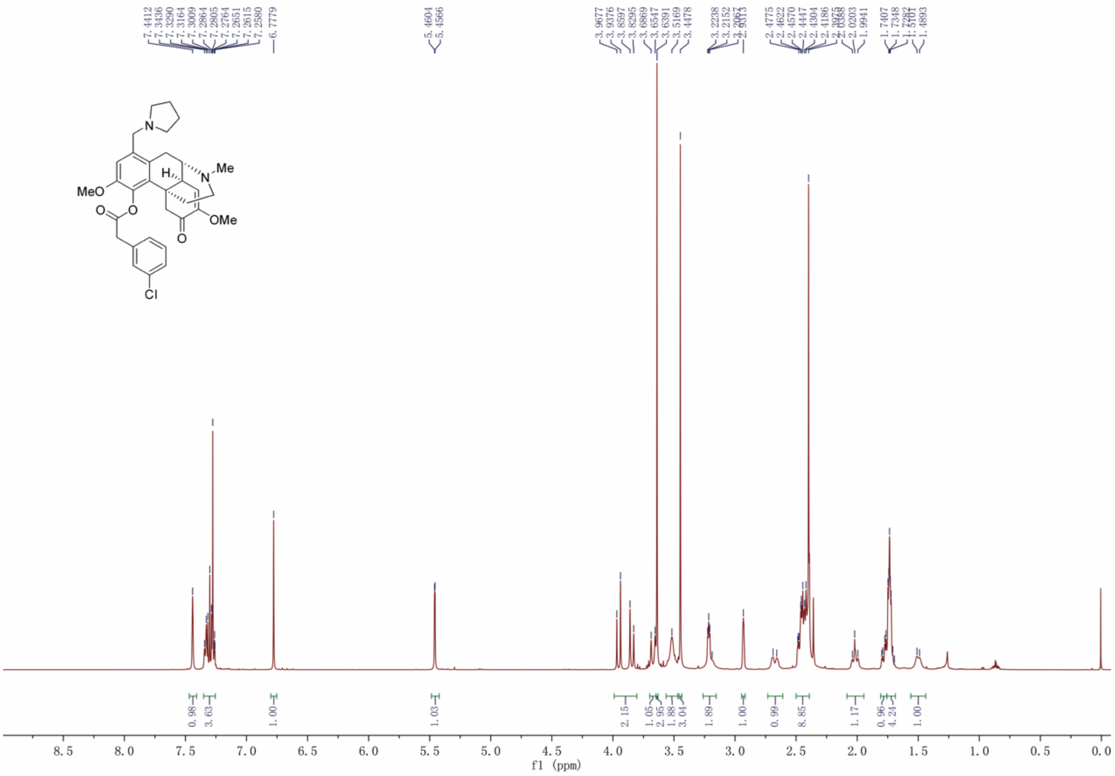

Figure S36. ¹H NMR spectrum of compound **6e**.

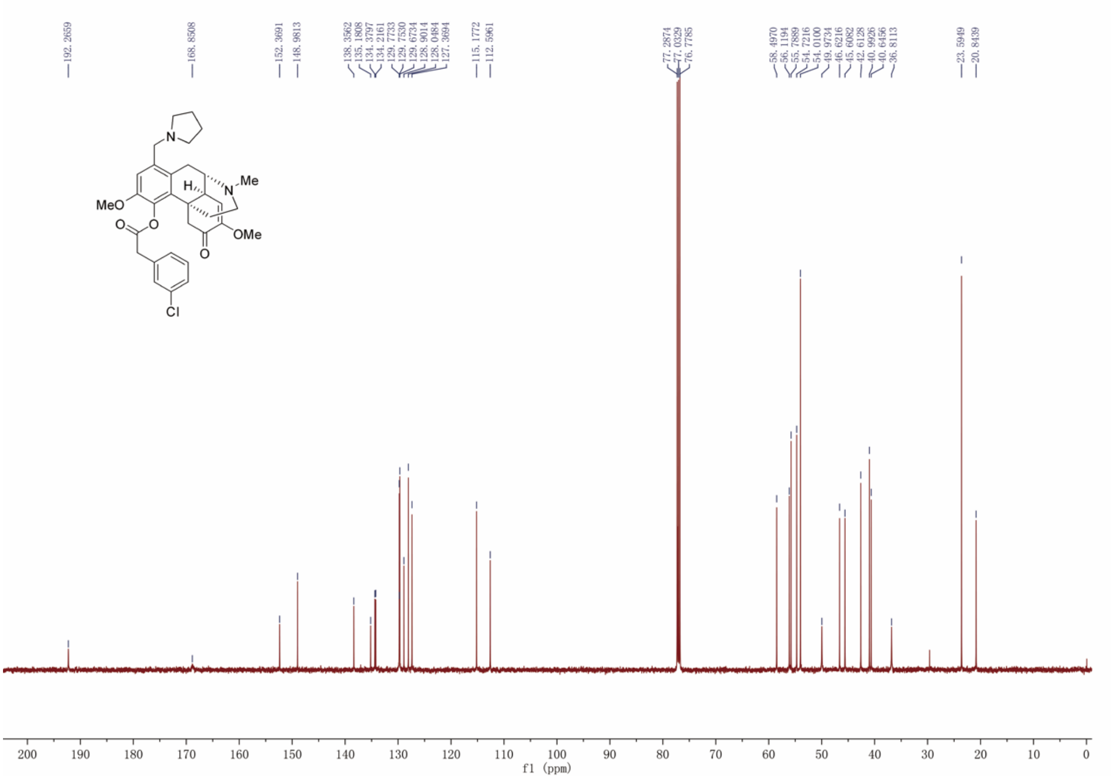

Figure S37. ^{13}C NMR spectrum of compound **6e**.

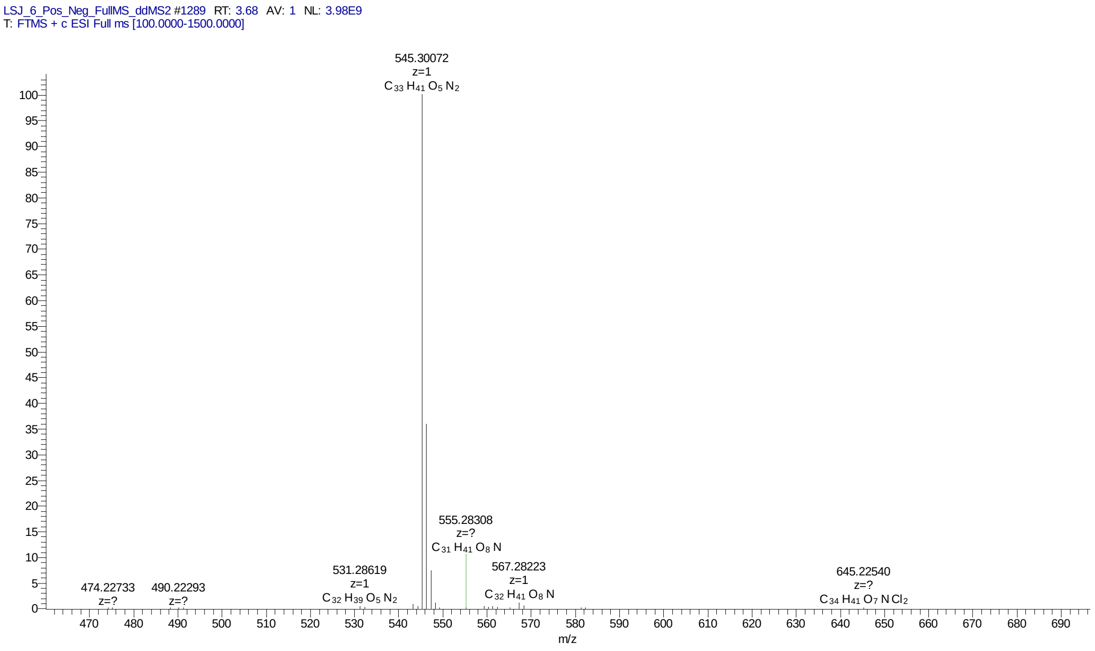

Figure S38. HRMS spectrum of compound **6f**.

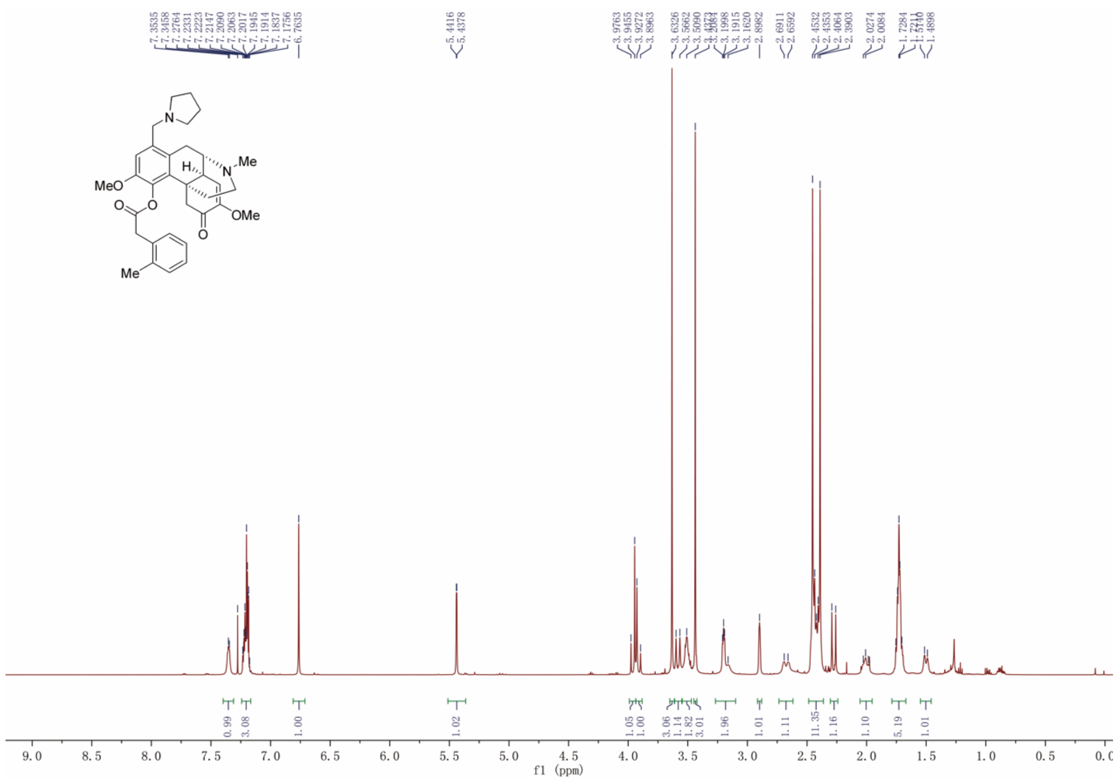

Figure S39. ^{1}H NMR spectrum of compound **6f**.

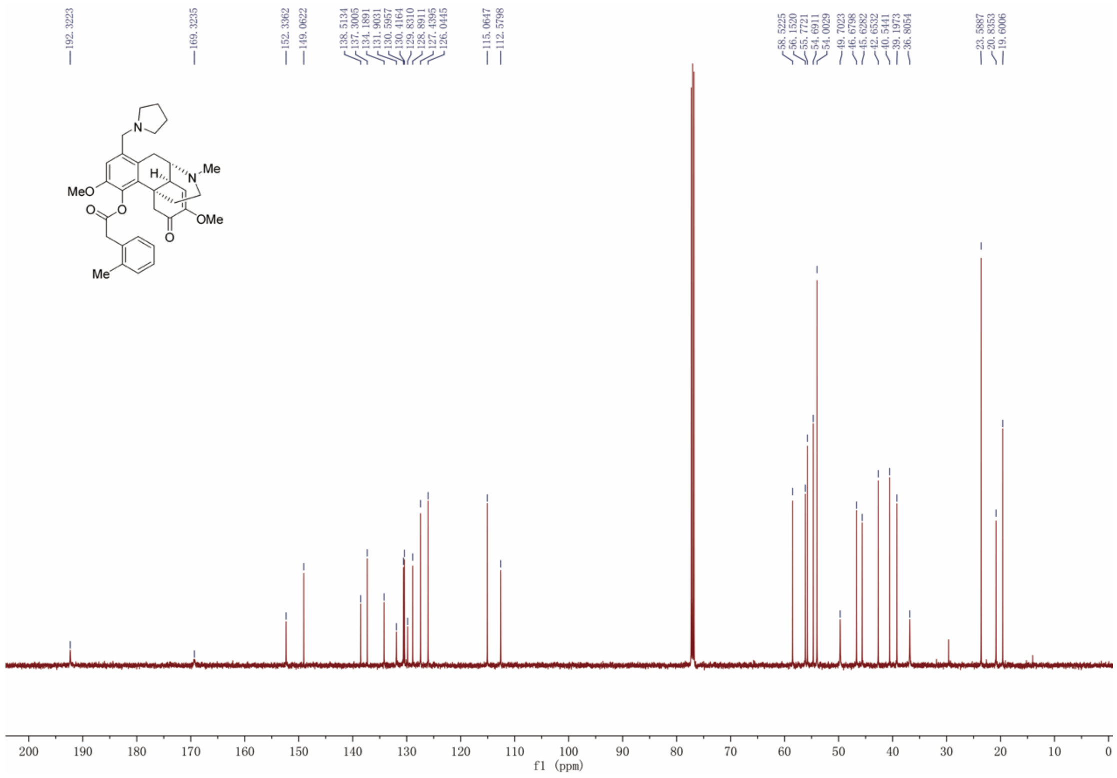

Figure S40. ^{13}C NMR spectrum of compound **6f**.

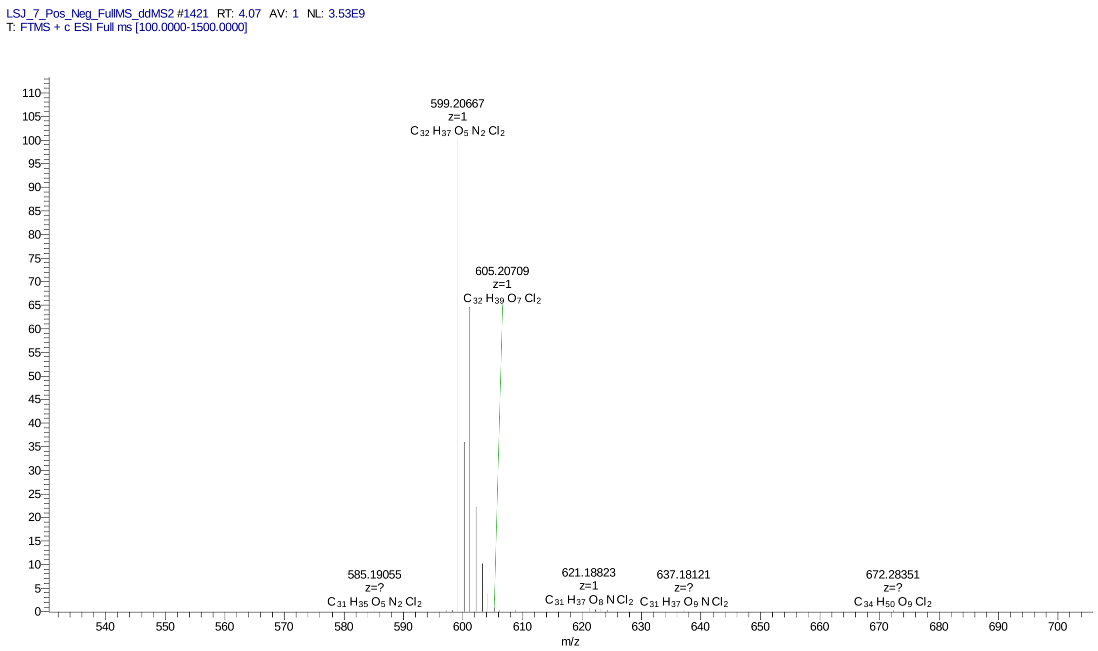

Figure S41. HRMS spectrum of compound **6g**.

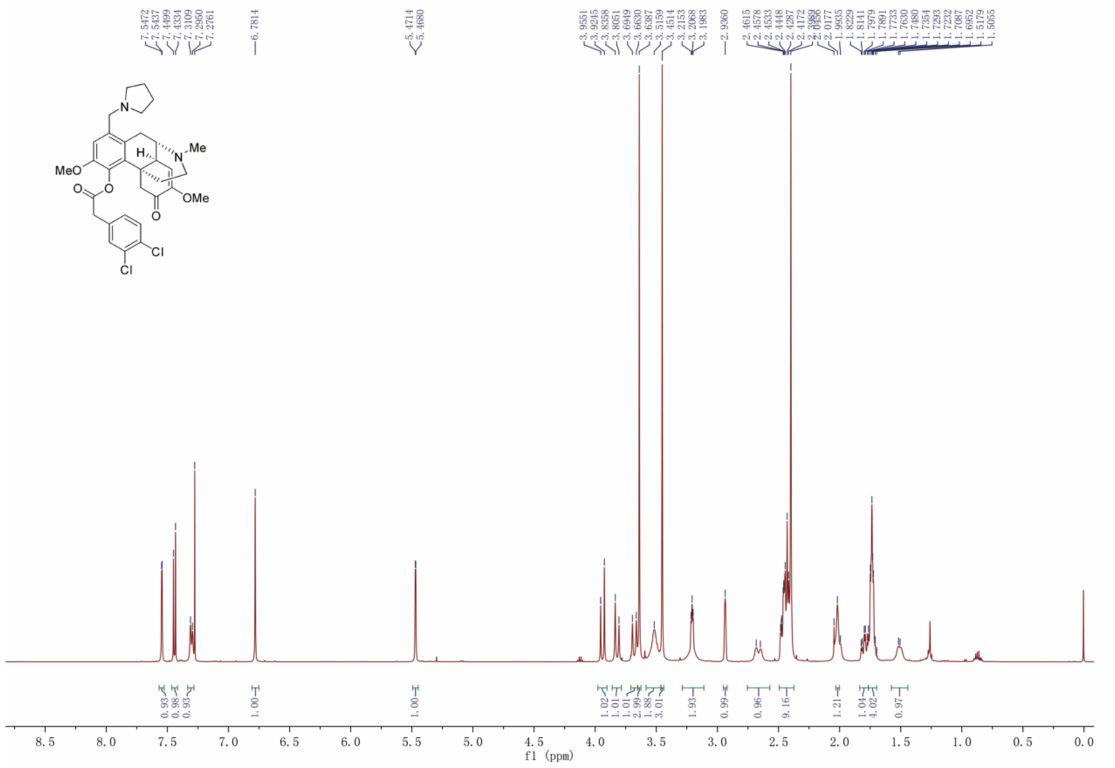

Figure S42. ^{1}H NMR spectrum of compound **6g**.

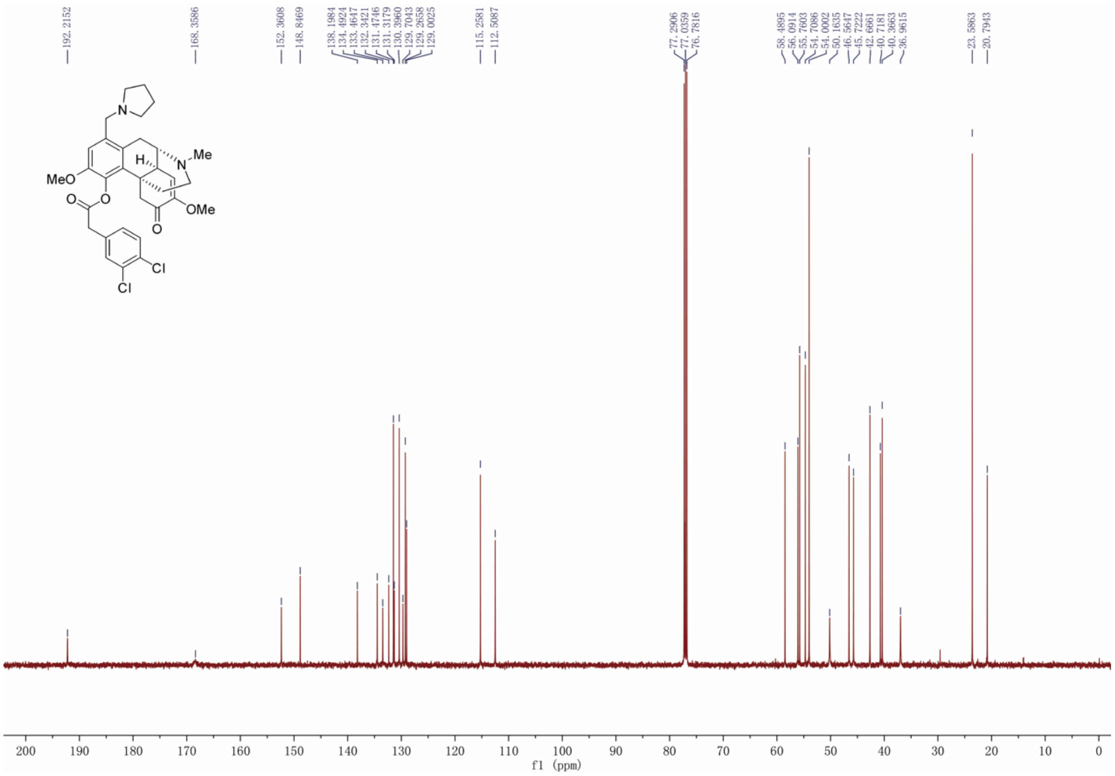

Figure S43. ^{13}C NMR spectrum of compound **6g**.

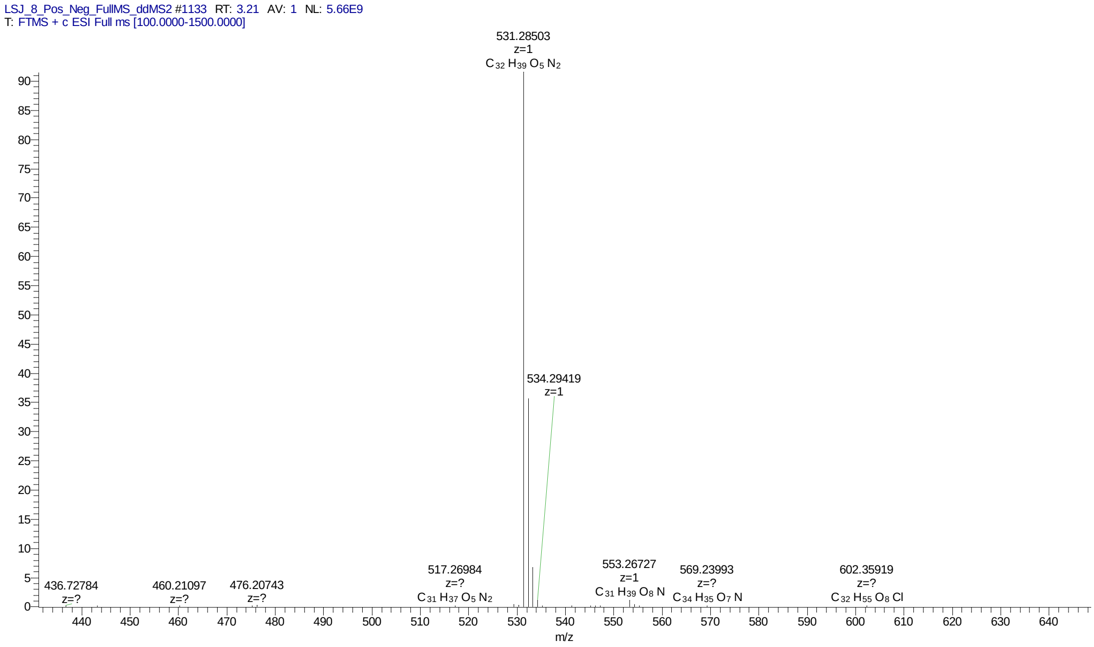

Figure S44. HRMS spectrum of compound **6h**.

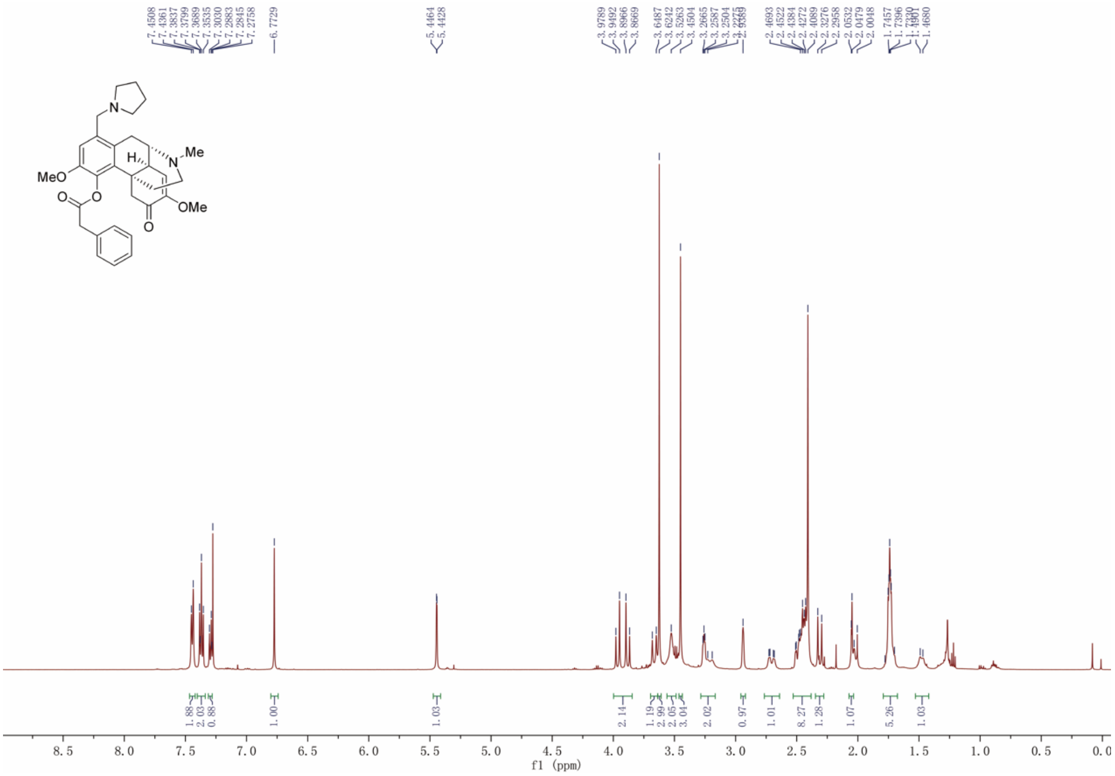

Figure S45. ^{1}H NMR spectrum of compound **6h**.

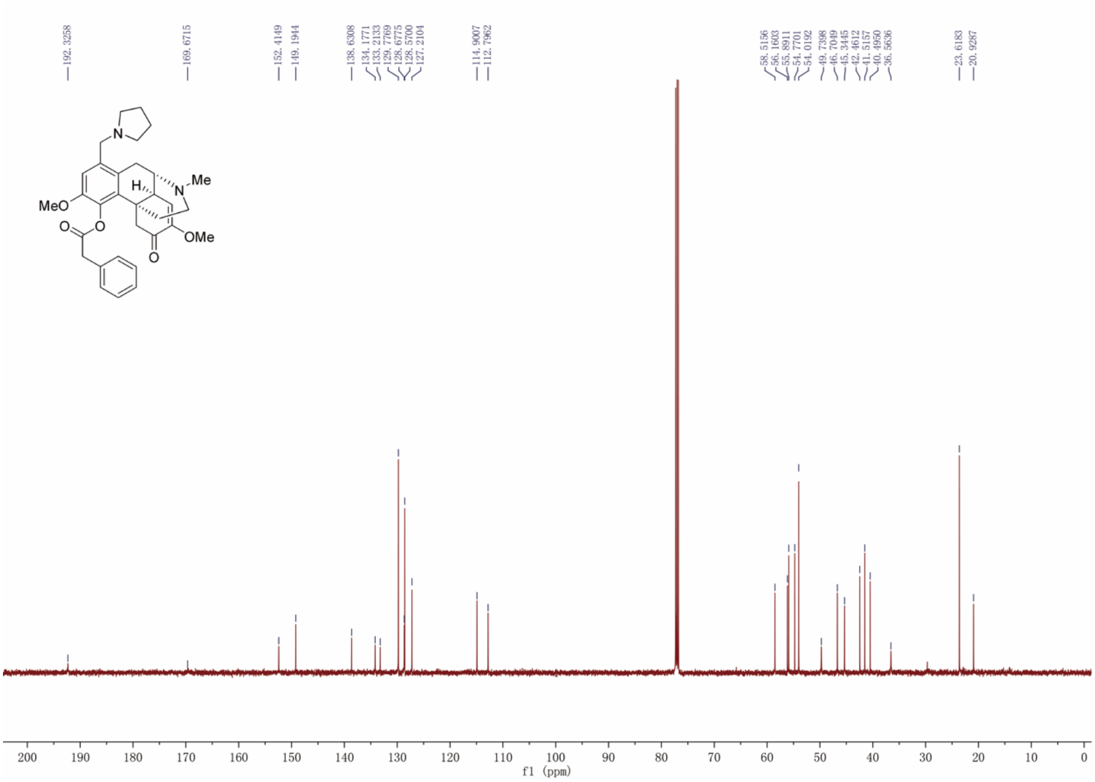

Figure S46. ^{13}C NMR spectrum of compound **6h**.

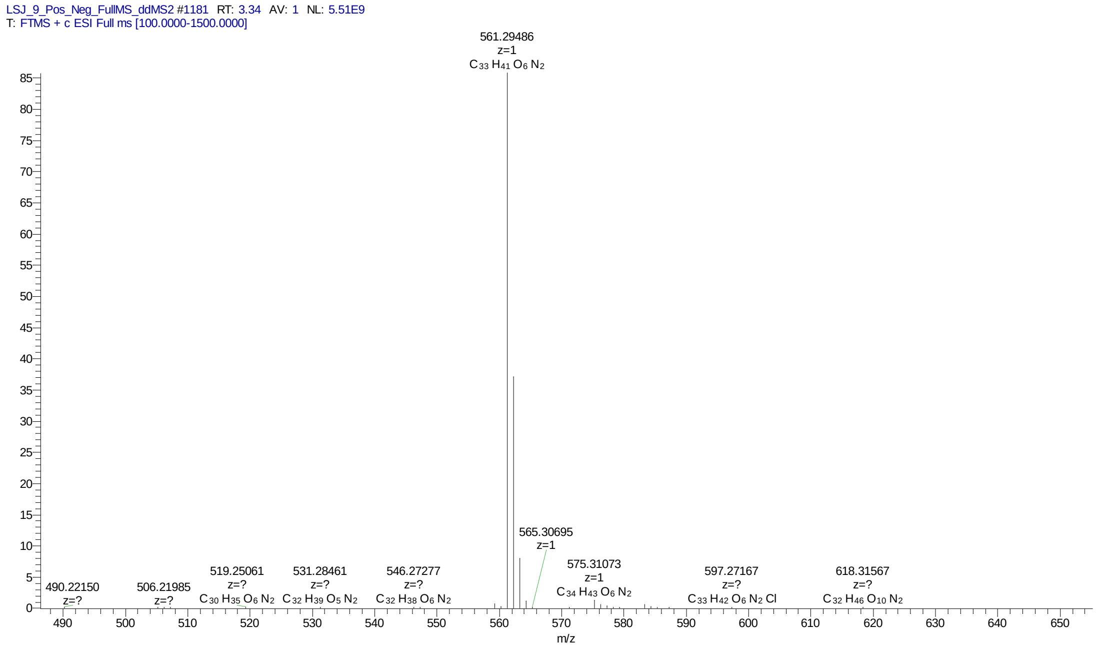

Figure S47. HRMS spectrum of compound **6i**.

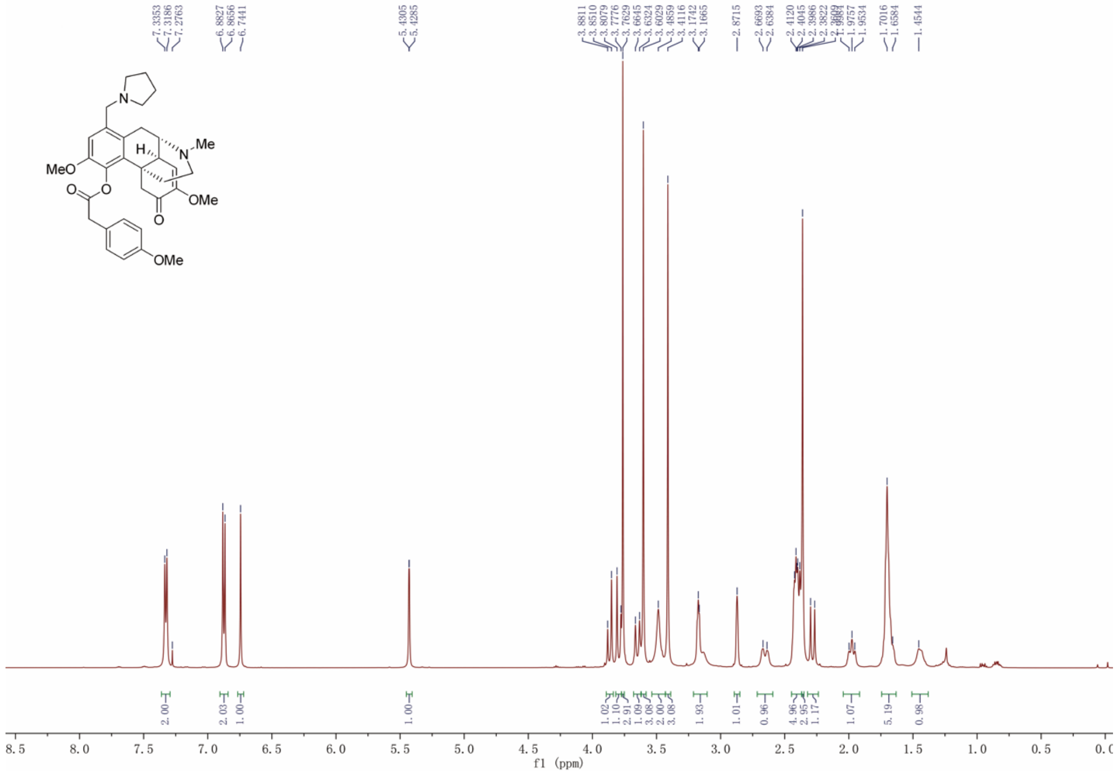

Figure S48. ^{1}H NMR spectrum of compound **6i**.

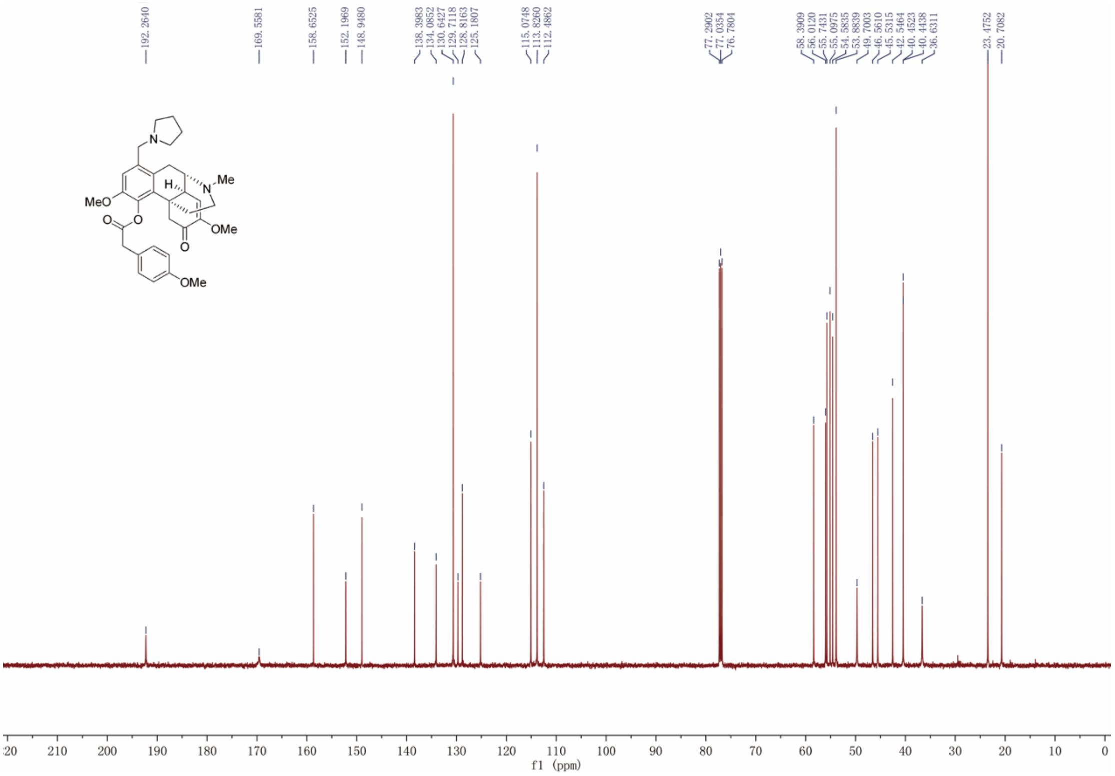

Figure S49. ^{13}C NMR spectrum of compound **6i**.

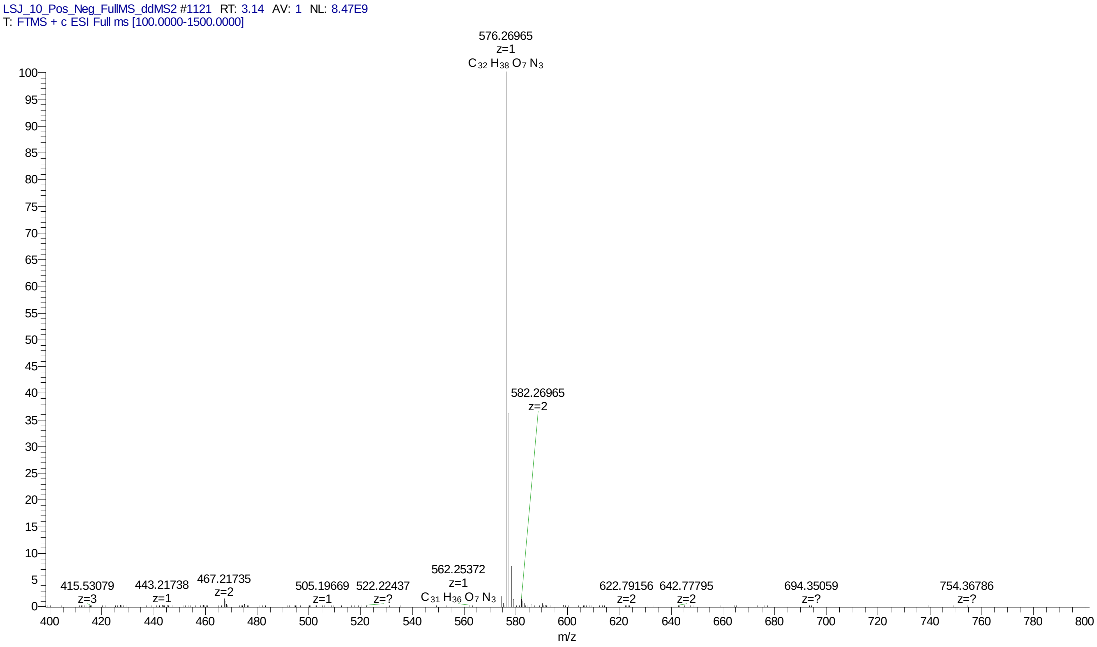

Figure S50. HRMS spectrum of compound **6j**.

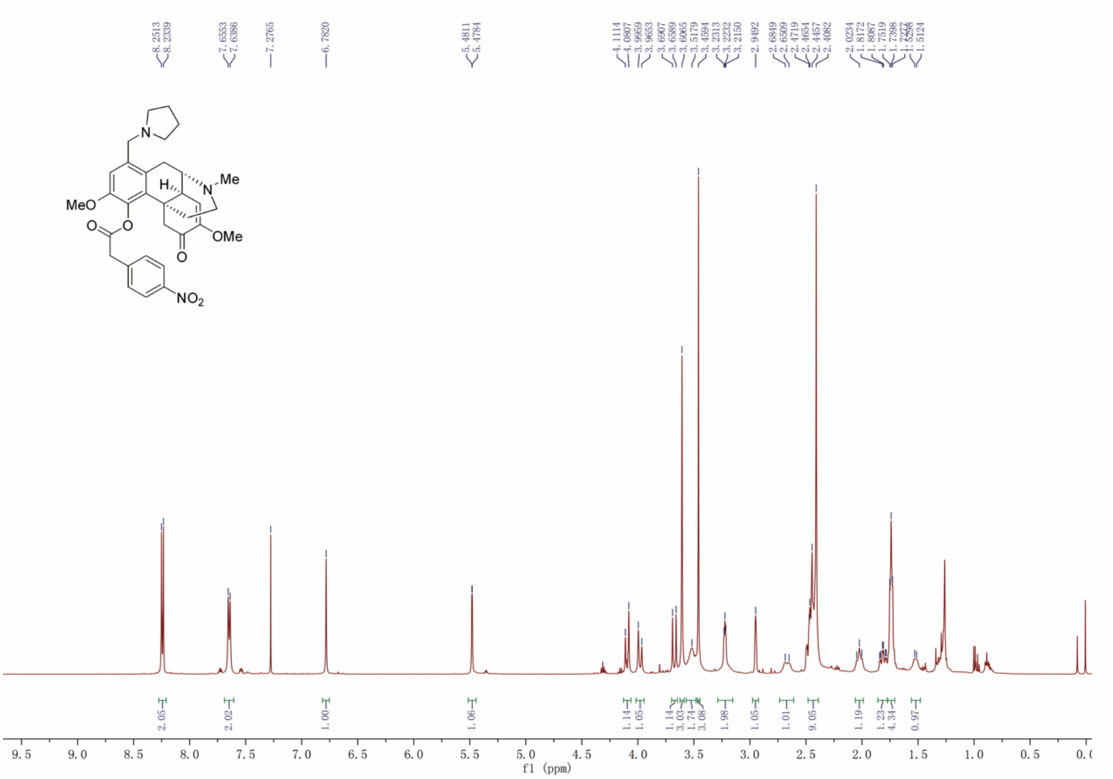

Figure S51. ^{1}H NMR spectrum of compound **6j**.

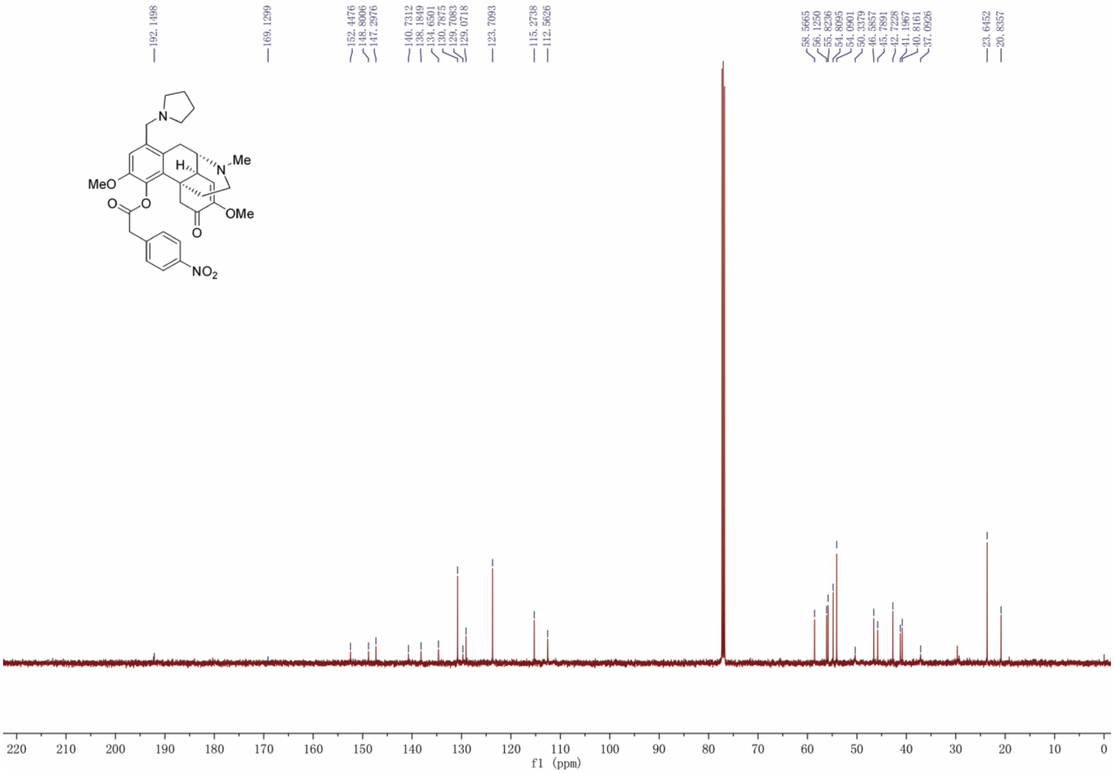

Figure S52. ^{13}C NMR spectrum of compound **6j**.

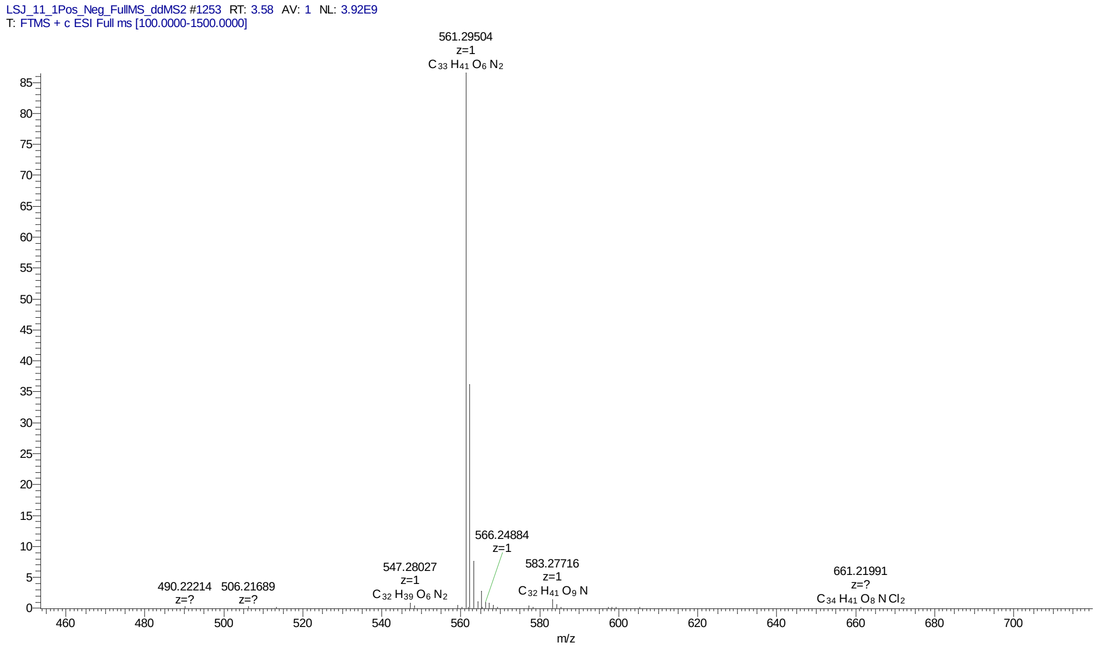

Figure S53. HRMS spectrum of compound **6k**.

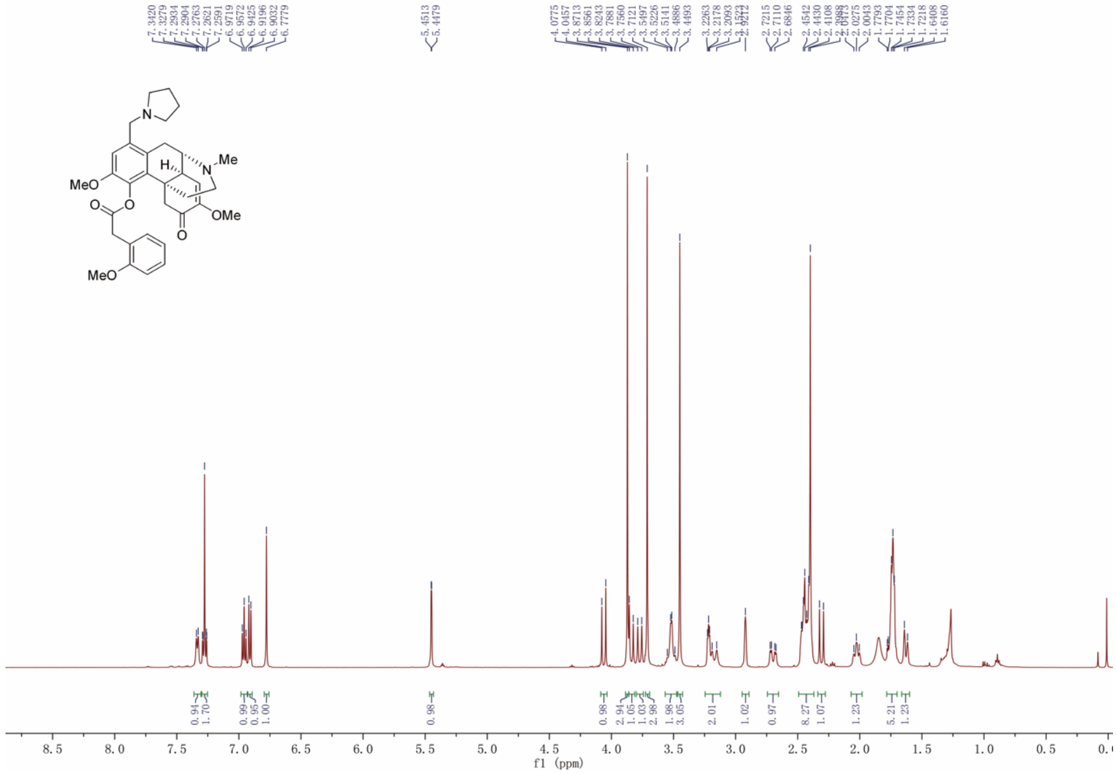

Figure S54. ^{1}H NMR spectrum of compound **6k**.

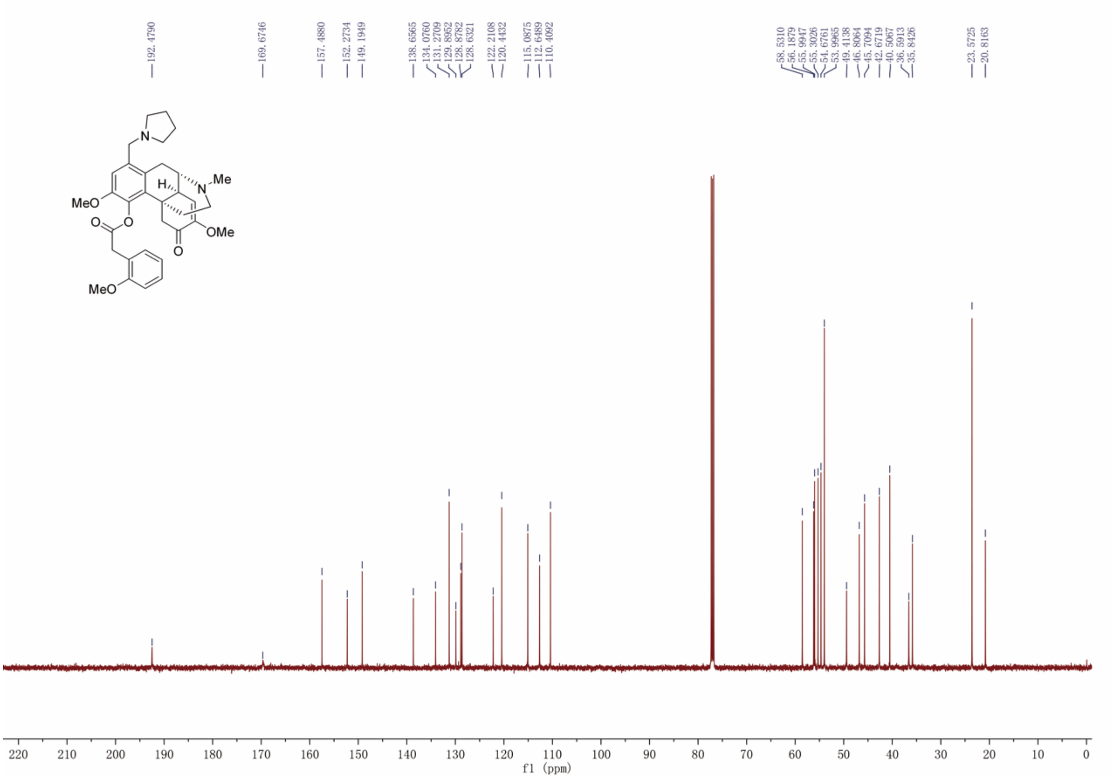

Figure S55. ^{13}C NMR spectrum of compound **6k**.

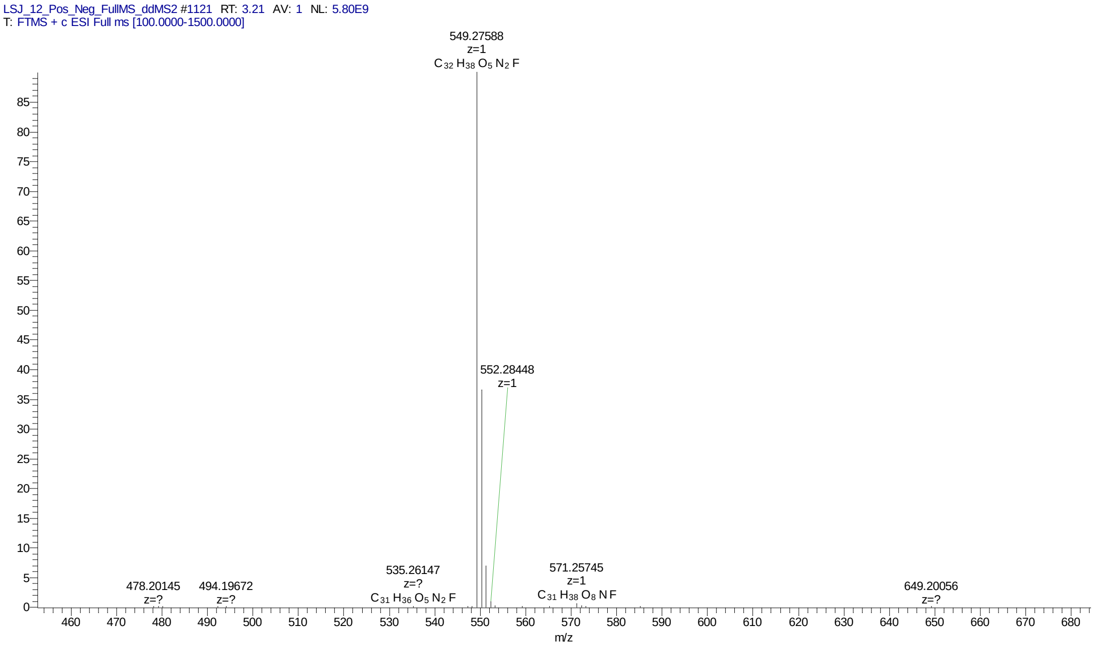

Figure S56. HRMS spectrum of compound **6l**.

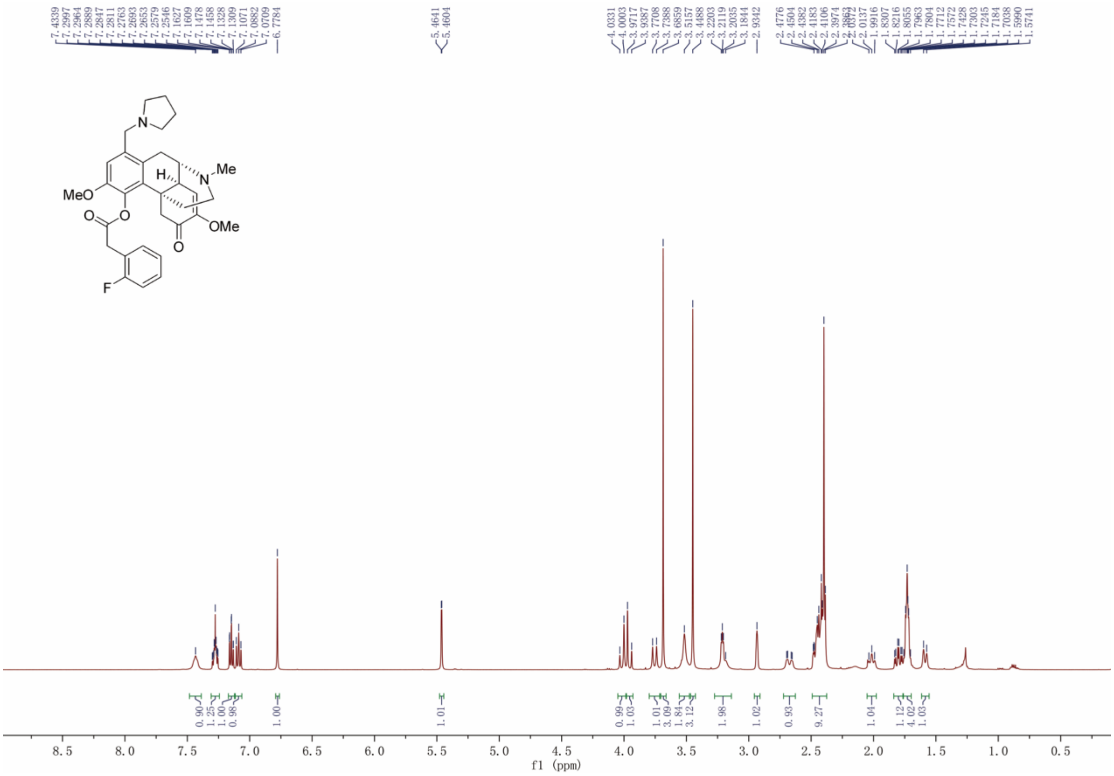

Figure S57. ^{1}H NMR spectrum of compound **6l**.

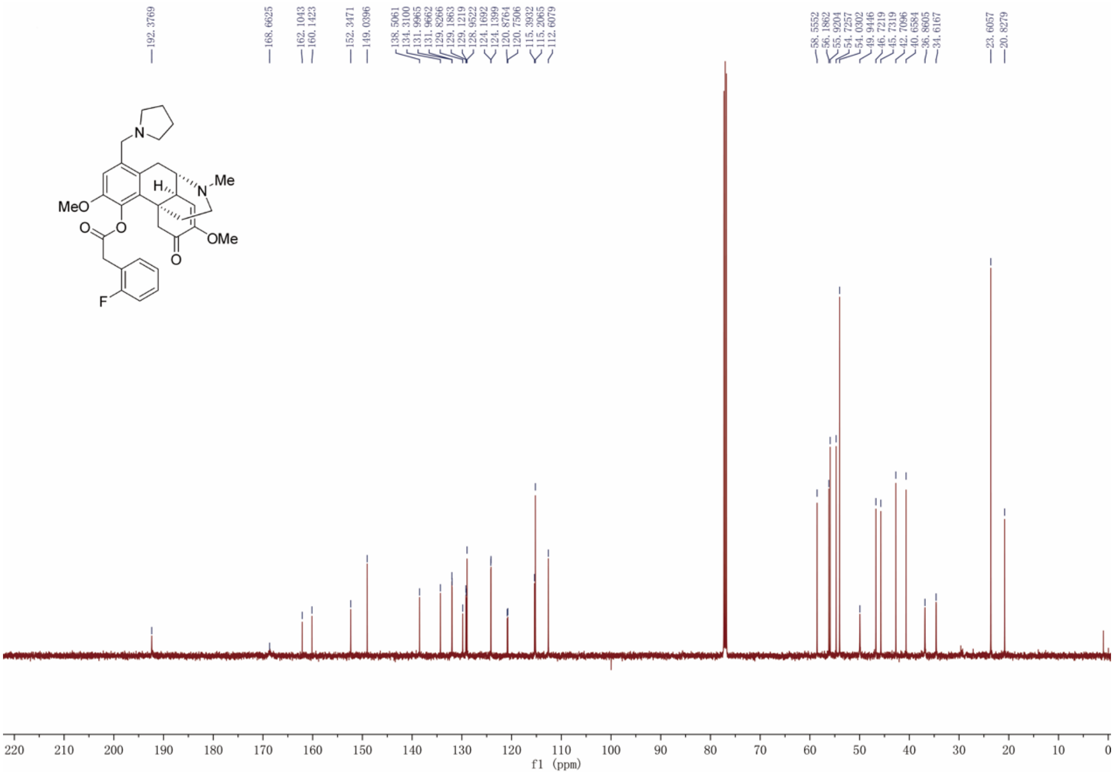

Figure S58. ^{13}C NMR spectrum of compound **6l**.

Table S1. Mean ± SD values of growth inhibition ratios at 2.5 μm drug concentration.

Comp.	Hela	MCF-7	SW480	A549	Hek293
**6a**	7.81 ± 5.42	0.00	12.92 ± 6.52	3.41 ±0.82	17.92 ± 1.86
**6b**	2.03 ± 2.29	3.63 ± 3.16	22.90 ± 6.49	0.00	0.66 ± 1.14
**6c**	3.05 ± 3.91	0.00	28.29 ± 6.91	16.29 ±0.63	13.81 ± 2.27
**6d**	10.26 ± 4.44	0.00	29.09 ± 6.92	29.59 ±0.16	42.56 ± 0.84
**6e**	3.21 ± 2.37	6.92 ± 2.62	29.45 ± 7.71	25.64 ±1.00	14.40 ± 2.54
**6f**	7.82 ± 4.10	0.00	20.78 ± 5.63	24.96 ±1.05	16.76 ± 2.39
**6g**	35.30 ± 4.37	6.59 ± 4.98	11.81 ± 7.50	20.29 ±0.78	9.72 ± 1.96
**6h**	11.21 ± 10.01	5.79 ± 10.03	28.24 ± 10.35	20.64 ±0.77	17.38 ± 1.49
**6i**	7.79 ± 6.01	11.68 ± 5.99	28.56 ± 9.02	23.54 ±0.76	13.27 ± 0.21
**6j**	0.00	3.64 ± 6.30	0.00	15.42 ±5.60	20.51 ± 6.76
**6k**	0.00	0.75 ± 1.29	3.72 ± 1.80	16.57 ±2.30	13.94 ± 4.06
**6l**	0.61 ± 0.60	4.61 ± 4.79	3.91 ± 0.85	13.17 ±4.88	12.69 ± 2.89

Table S2. Mean ± SD values of growth inhibition ratios at 25 μm drug concentration.

Comp.	Hela	MCF-7	SW480	A549	Hek293
**6a**	88.49 ± 0.67	85.28 ± 1.62	82.31 ± 4.36	37.39 ± 0.39	61.06 ± 4.71
**6b**	11.48 ± 4.88	2.70 ± 4.67	29.49 ± 11.23	30.87 ± 0.32	45.90 ± 1.35
**6c**	53.46 ± 2.45	41.99 ± 3.02	58.38 ± 7.58	49.42 ± 0.59	54.61 ± 2.29
**6d**	93.10 ± 0.17	88.61 ± 2.73	94.16 ± 3.21	93.02 ± 0.20	84.57 ± 4.34
**6e**	80.93 ± 2.05	77.85 ± 0.45	61.02 ± 7.14	61.06 ± 0.13	63.28 ± 11.39
**6f**	58.74 ± 2.64	31.39 ± 3.72	55.73 ± 6.94	57.09 ± 0.06	64.34 ± 14.59
**6g**	93.85 ± 0.20	34.57 ± 2.06	55.95 ± 5.88	43.88 ± 1.11	59.91 ± 16.92
**6h**	21.18 ± 4.40	0.00	37.79 ± 9.83	52.29 ± 0.76	45.96 ± 22.40
**6i**	29.24 ± 5.29	0.00	38.93 ± 9.65	51.77 ± 0.50	46.91 ± 16.67
**6j**	3.75 ± 0.91	1.97 ± 2.75	0.92 ± 1.34	38.67 ± 8.59	56.07 ± 7.28
**6k**	44.12 ± 0.92	24.38 ± 10.99	36.88 ± 0.52	51.92 ± 6.36	63.46 ± 10.25
**6l**	28.96 ± 1.88	9.52 ± 8.26	16.49 ± 0.53	47.07 ± 5.40	68.77 ± 13.28

Table S3. The total energy for the tested compounds **5a-5k** against docked proteins binding pockets.

Comp. No	AKT1	CCND1	EGFR	GADPH	HRAS
	PDB ID: 4EJN	PDB ID: 2W96	PDB ID: 1M17	PDB ID: 1QXS	PDB ID: 121P
	Total energy (kcal/mol)				
5a	-110.4517	-92.014943	-113.49261	-96.9778	-121.525
5b	-109.487	-92.801225	-100.97898	-102.746	-112.007
5c	-152.56195	-92.328519	-115.86657	-118.758	-147.565
5d	-109.98437	-100.28895	-105.85058	-106.27	-139.565
5e	-136.21449	-90.093949	-104.47254	-97.2435	-109.701
5f	-134.21769	-85.643314	-108.24462	-96.5164	-124.008
5g	-117.78228	-92.676583	-106.77327	-105.627	-109.648
5h	-114.13335	-96.377126	-110.5033	-103.961	-132.392
5i	-112.55216	-85.210883	-103.1928	-107.965	-114.3
5j	-120.06915	-97.683944	-117.15296	-97.7188	-118.017
5k	-131.10572	-86.667097	-99.041469	-102.411	-125.194
sinomenine	-96.90419	-66.984998	-74.70841	-74.5722	-82.9715

Comp. No	IL6	MYC	PTEN	STAT3	TP53	VEGFA
	PDB ID: 1N26	PDB ID: 5I50	PDB ID: 1D5R	PDB ID: 6NJS	PDB ID: 2H59	PDB ID: 1FLT
	Total energy (kcal/mol)					
5a	-75.779181	-8.974544	-94.945824	-100.46753	-96.439231	-71.452019
5b	-82.592442	-12.3089	-92.312345	-92.584822	-96.639242	-54.26271
5c	-76.288248	-55.318323	-97.488565	-104.86825	-110.11159	-90.02963
5d	-75.881368	-34.276427	-102.03249	-96.074973	-95.823975	-75.392292

5e	-68.383119	-10.428299	-74.778382	-104.45959	-91.707905	-70.406066
5f	-71.536604	-40.056095	-88.752568	-88.94488	-105.39038	-71.933654
5g	-71.232017	-40.452157	-113.77743	-103.26589	-111.89281	-79.008219
5h	-80.671686	-44.509336	-93.003952	-86.437455	-115.43665	-72.333438
5i	-62.890056	-13.745778	-92.634638	-93.805265	-95.467208	-75.814431
5j	-75.633935	-34.652115	-93.927298	-86.887086	-96.537606	-70.061337
5k	-67.495732	-41.877304	-107.55105	-98.08101	-124.89364	-72.480579
sinomenine	-53.674125	-41.524897	-64.40118	-66.789697	-79.710613	-51.953652

Table S4. The total energy for the tested compounds **6a-6l** against docked proteins binding pockets.

Comp. No	AKT1	CCND1	EGFR	ERBB2	GADPH	HRAS
	PDB ID: 4EJN	PDB ID: 2W96	PDB ID: 1M17	PDB ID: 3PP0	PDB ID: 1QXS	PDB ID: 121P
	Total energy (kcal/mol)					
6a	-155.152542	-85.819282	-102.71573	-97.914798	-89.3104	-126.074
6b	-143.51096	-93.702009	-115.57974	-111.85183	-95.4181	-112.662
6c	-117.814818	-94.091713	-105.07849	-96.038604	-96.8829	-120.792
6d	-134.025843	-82.064413	-103.10902	-106.17696	-96.7202	-133.267
6e	-129.107106	-92.365618	-101.99209	-108.70016	-94.2277	-105.098
6f	-121.550631	-92.204911	-113.66322	-109.7887	-103.834	-117.736
6g	-104.509119	-87.905554	-106.56283	-96.849524	-106.202	-117.264
6h	-110.348304	-97.900196	-105.29039	-84.360193	-107.937	-125.747
6i	-116.615593	-92.693974	-100.71573	-93.429995	-110.16	-144.341
6j	-139.997316	-96.545364	-112.1828	-112.68309	-101.915	-134.795
6k	-124.013157	-91.581717	-116.20325	-94.416143	-99.9461	-116.475

6l	-136.820447	-93.027433	-109.59803	-105.45488	-105.253	-127.087
sinomenine	-96.102089	-71.538623	-75.796964	-70.356615	-74.5722	-82.9715

Comp. No	IL6	KARS	MYC	PTEN	STAT3	TP53	VEGFR
	PDB ID: 1N26	PDB ID: 4LYH	PDB ID: 5I50	PDB ID: 1D5R	PDB ID: 6NJS	PDB ID: 2H59	PDB ID: 1FLT
	Total energy (kcal/mol)						
6a	-71.647855	-132.867067	-40.080337	-91.606693	-99.476774	-102.313	-81.769509
6b	-75.468949	-117.201122	-21.638076	-108.52824	-100.66394	-109.071	-68.587673
6c	-68.965233	-110.057307	-29.018839	-92.061217	-88.464252	-105.239	-64.756449
6d	-78.230692	-113.109424	-52.036912	-112.64237	-97.491569	-105.46	-58.840218
6e	-71.342559	-124.720135	-37.966443	-94.311978	-90.146547	-100.326	-78.221192
6f	-77.711822	-123.487573	-41.312645	-109.66785	-96.277285	-103.482	-72.609809
6g	-60.853169	-117.865833	-4.275516	-96.63994	-85.11816	-97.4213	-74.759565
6h	-83.204263	-127.253945	-38.630007	-104.81761	-93.077551	-94.8547	-65.555756
6i	-77.290155	-127.243938	-34.587211	-121.89391	-91.794092	-97.0393	-68.257331
6j	-71.596769	-128.724795	-40.534216	-119.81884	-117.91884	-104.012	-72.987394
6k	-90.086292	-113.991245	-50.336598	-103.78144	-93.282852	-105.069	-74.469279
6l	-73.523545	-125.62224	-30.564061	-86.536363	-96.197188	-91.2632	-66.015682
sinomenine	-51.477432	-81.898493	-39.729746	-61.187369	-65.431933	-96.7829	-48.755511

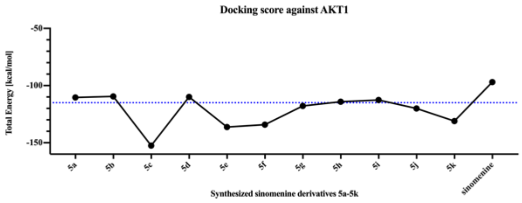
Docking score against AKT1
Total Energy [kcal/mol]
-50
-100
-150
5a 5b 5c 5d 5e 5f 5g 5h 5i 5j 5k sinomenine
Synthesized sinomenine derivatives 5a-5k

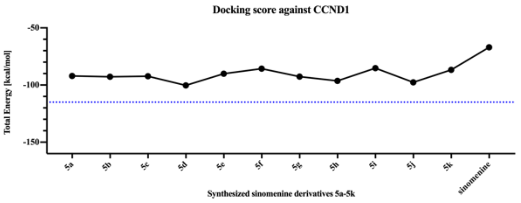
Docking score against CCND1
Total Energy [kcal/mol]
-50
-100
-150
5a 5b 5c 5d 5e 5f 5g 5h 5i 5j 5k sinomenine
Synthesized sinomenine derivatives 5a-5k

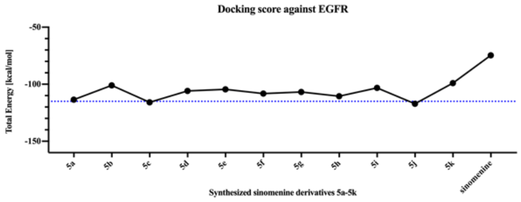
Docking score against EGFR
Total Energy [kcal/mol]
-50
-100
-150
5a 5b 5c 5d 5e 5f 5g 5h 5i 5j 5k sinomenine
Synthesized sinomenine derivatives 5a-5k

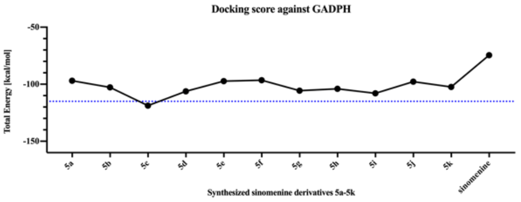
Docking score against GADPH
Total Energy [kcal/mol]
-50
-100
-150
5a 5b 5c 5d 5e 5f 5g 5h 5i 5j 5k sinomenine
Synthesized sinomenine derivatives 5a-5k

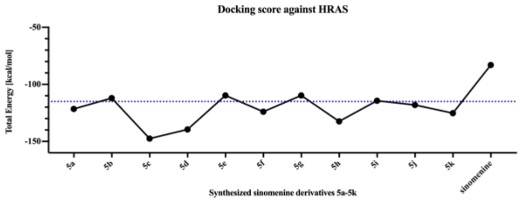
Docking score against HRAS
Total Energy [kcal/mol]
-50
-100
-150
5a 5b 5c 5d 5e 5f 5g 5h 5i 5j 5k sinomenine
Synthesized sinomenine derivatives 5a-5k

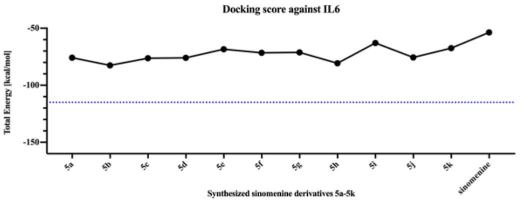
Docking score against IL6
Total Energy [kcal/mol]
-50
-100
-150
5a 5b 5c 5d 5e 5f 5g 5h 5i 5j 5k sinomenine
Synthesized sinomenine derivatives 5a-5k

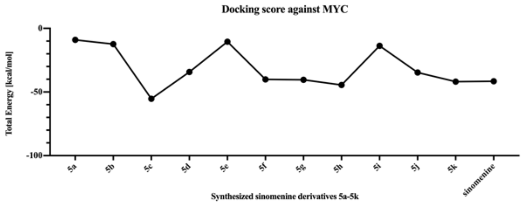
Docking score against MYC
Total Energy [kcal/mol]
0
-50
-100
5a 5b 5c 5d 5e 5f 5g 5h 5i 5j 5k sinomenine
Synthesized sinomenine derivatives 5a-5k

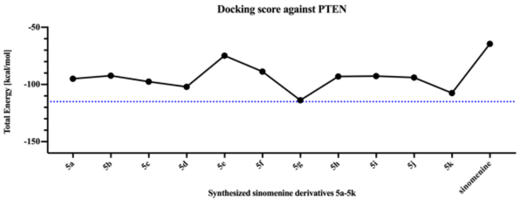
Docking score against PTEN
Total Energy [kcal/mol]
-50
-100
-150
5a 5b 5c 5d 5e 5f 5g 5h 5i 5j 5k sinomenine
Synthesized sinomenine derivatives 5a-5k

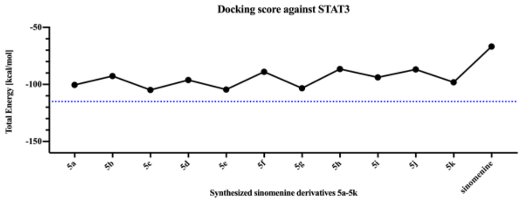
Docking score against STAT3
Total Energy [kcal/mol]
-50
-100
-150
5a 5b 5c 5d 5e 5f 5g 5h 5i 5j 5k sinomenine
Synthesized sinomenine derivatives 5a-5k

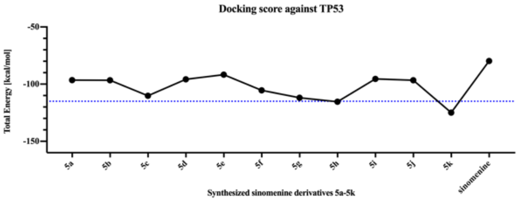
Docking score against TP53
Total Energy [kcal/mol]
-50
-100
-150
5a 5b 5c 5d 5e 5f 5g 5h 5i 5j 5k sinomenine
Synthesized sinomenine derivatives 5a-5k

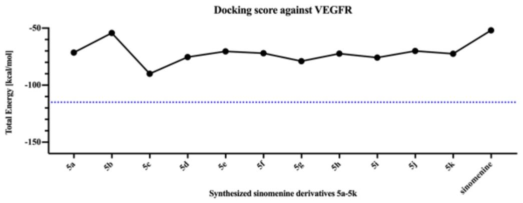
Docking score against VEGFR
Total Energy [kcal/mol]
-50
-100
-150
5a
5b
5c
5d
5e
5f
5g
5h
5i
5j
5k
sinomenine
Synthesized sinomenine derivatives 5a-5k

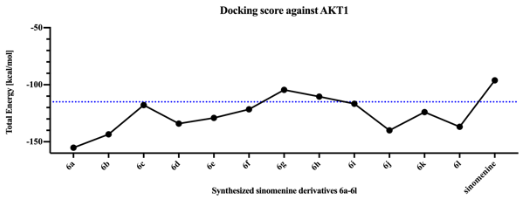
Docking score against AKT1
Total Energy [kcal/mol]
-50
-100
-150
6a
6b
6c
6d
6e
6f
6g
6h
6i
6j
6k
6l
sinomenine
Synthesized sinomenine derivatives 6a-6l

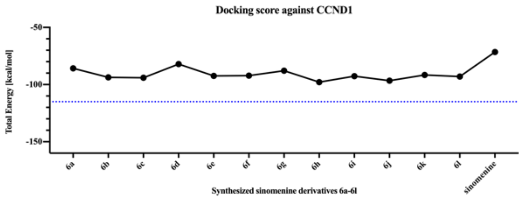
Docking score against CCND1
Total Energy [kcal/mol]
-50
-100
-150
6a
6b
6c
6d
6e
6f
6g
6h
6i
6j
6k
6l
sinomenine
Synthesized sinomenine derivatives 6a-6l

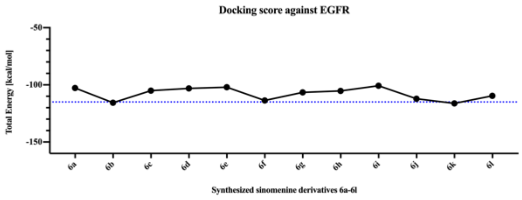
Docking score against EGFR
Total Energy [kcal/mol]
-50
-100
-150
6a
6b
6c
6d
6e
6f
6g
6h
6i
6j
6k
6l
Synthesized sinomenine derivatives 6a-6l

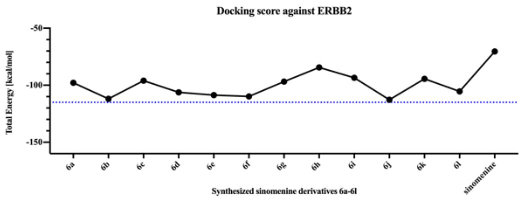
Docking score against ERBB2
Total Energy [kcal/mol]
-50
-100
-150
6a
6b
6c
6d
6e
6f
6g
6h
6i
6j
6k
6l
sinomenine
Synthesized sinomenine derivatives 6a-6l

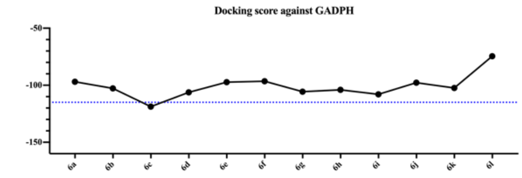
Docking score against GADPH
-50
-100
-150
6a
6b
6c
6d
6e
6f
6g
6h
6i
6j
6k
6l

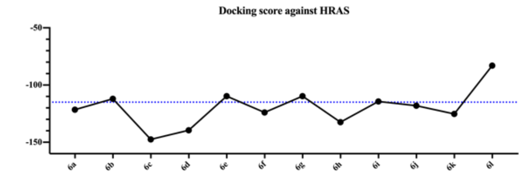
Docking score against HRAS
-50
-100
-150
6a
6b
6c
6d
6e
6f
6g
6h
6i
6j
6k
6l

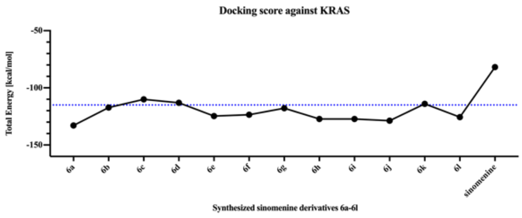
Docking score against KRAS
Total Energy [kcal/mol]
-50
-100
-150
6a
6b
6c
6d
6e
6f
6g
6h
6i
6j
6k
6l
sinomenine
Synthesized sinomenine derivatives 6a-6l

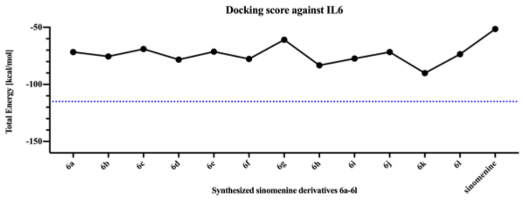
Docking score against IL6
Total Energy [kcal/mol]
-50
-100
-150
6a
6b
6c
6d
6e
6f
6g
6h
6i
6j
6k
6l
sinomenine
Synthesized sinomenine derivatives 6a-6l

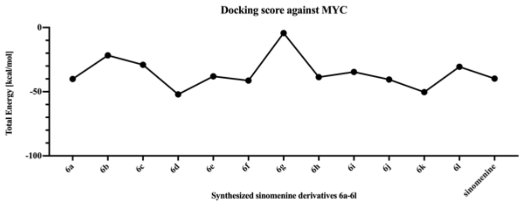
Docking score against MYC
Total Energy [kcal/mol]
0
-50
-100
6a
6b
6c
6d
6e
6f
6g
6h
6i
6j
6k
6l
sinomenine
Synthesized sinomenine derivatives 6a-6l

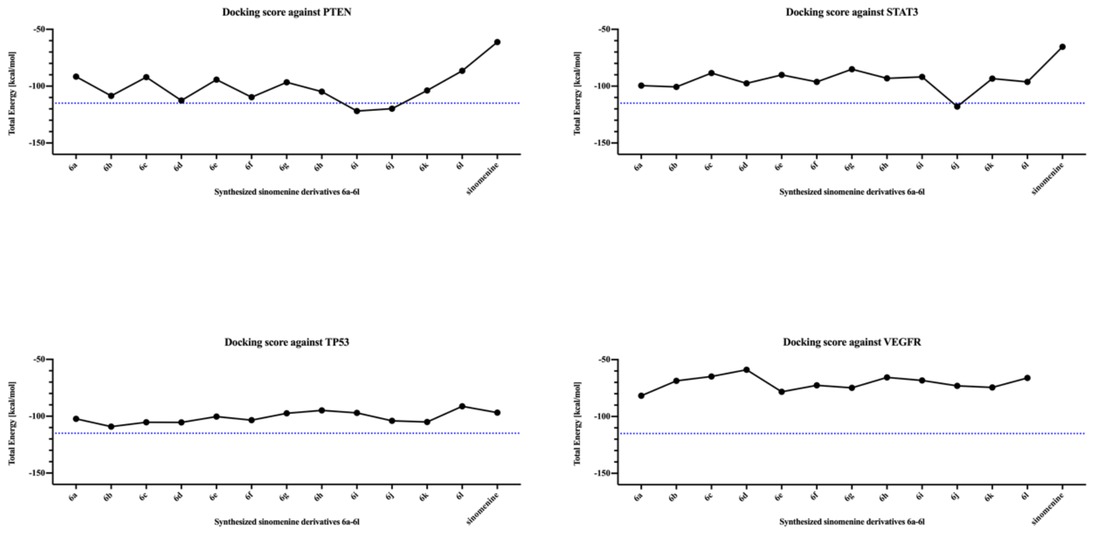

Figure S59. The total energy of molecular docking between compounds and each target.

Table S5. The various interactions for the most promising test compounds into the AKT1 binding pockets.

AKT1									
Comp. No	Hydrogen Bonds		Hydrophobic Interactions	Salt Bridges		π-Stacking		Halogen Bonds	
	Length Å	AA	AA	Length Å	AA	Length Å	AA	Length Å	AA
5g	2.52<br>2.86	ASN54A<br>GLN79A	GLN79A, TRP80A, LEU202A, LEU210A, LEU264A, VAL270A	-		-		-	
5i	3.42<br>2.7	ASN53A<br>GLN59A	ASN53A, TRP80A, VAL270A	-		-		-	
5j	2.51<br>2.99	ASN54A<br>GLN79A	THR82A, ILE84A, TYR272A, ARG273A, ASP274A, GLU298A	4.73	ASP274A	-		-	
6a	2.2<br>1.73	THR81A<br>THR211A	TRP80A, ILE84A, LEU210A, LEU264A, VAL270A, ASP292A	-		-		-	
6d	2.5<br>2.48<br>2.46	ASN54A<br>GLN79A<br>THR81A	TRP80A, THR82A, LEU210A, LEU264A, VAL270A, TYR272A, ASP274A	5.5	LYS179A	-		-	
6e	2.17<br>3.61<br>3.44<br>3.11	THR82A<br>LYS179A<br>PHE293A<br>GLY294A	GLN79A, TRP80A, THR82A, ILE84A, LEU264A, VAL270A, TYR272A, ASP274A	-		4.18	TRP80A	3.47	THR211A
6g	2.56<br>2.92	GLN59A<br>GLN79A	ASN53A, GLN79A, LEU202A	-		-		-	

AA: amino acids

Table S6. The various interactions for the most promising test compounds into the EGFR binding pockets.

EGFR							
Comp.	Hydrogen Bonds		Hydrophobic Interactions	Salt Bridges		Halogen Bonds	
No	Length Å	AA	AA	Length Å	AA	Length Å	AA
5i	2.34	ASP776A	LEU694A, PHE699A, VAL702A, LYS721A	-		-	
5j	2.71	GLY697A	LEU694A, PHE699A, VAL702A, ALA719A, LYS721A, LEU768A, LEU820A	-		3.31<br>3.67	ALA719A<br>LEU764A
5g	1.67	CYS773A	PHE699A, VAL702A, LYS721A, LEU768A, ARG817A, LEU820A	-		-	
6a	3.16	CYS773A	LEU694A, PHE699A, VAL702A, LYS721A, LEU764A, THR766A, LEU820A, THR830A	4.99	LYS721A	3.46	ARG817A
6d	1.91<br>2.52	MET769A<br>CYS773A	LEU694A, PHE699A, VAL702A, ALA719A, MET769A, ARG817A, LEU820A	-		3.22	ASP831A
6e	2.23<br>2.59	CYS773A<br>ASP776A	LEU694A, LEU694A, ALA719A, LEU768A, LEU820A	-		-	
6g	3.07	ARG817A	PHE699A, VAL702A, ARG817A, LEU820A	-		-	

AA: amino acids

Table S7. The various interactions for the most promising test compounds into the HRAS binding pockets.

HRAS									
Comp.	Hydrogen Bonds		Hydrophobic Interactions	Salt Bridges		Halogen Bonds		π-Stacking	
No	Length Å	AA	AA	Length Å	AA	Length Å	AA	Length Å	AA
5g	2.36<br>2.41	SER17A<br>ALA18A	-	3.71	LYS117A	-		-	
5i	2.48	LYS117A	TYR32A	4.01	LYS117A	3.98	GLU31A	-	
5j	-		ALA18A, PHE28A, VAL29A, TYR32A, LYS117A, LYS147A	2.47	LYS117A	-		-	
6a	3.32<br>2.69	SER17A<br>ALA18A	ALA18A, PHE28A, TYR32A, ALA146A	4.56	LYS117A	2.71	ASP119A	-	
6d	2.65<br>2.7<br>1.89<br>2.14<br>2.92<br>2.2	GLY13A<br>VAL14A<br>GLY15A<br>LYS16A<br>GLU31A<br>ASP33A	ALA18A, ILE21A, VAL29A, TYR32A, ASP33A	-		-		-	
6e	2.47<br>2.37	ASP30A<br>LYS117A	ALA18A, PHE28A, TYR32A, LYS117A, ALA146A, LYS147A	4.73	LYS117A	3.61	GLY15A	4.57	PHE28A
6g	3.32<br>2.37	ASP30A<br>ASP33A	LYS117A	3.54	LYS117A	2.96	ASN86A	-	

Table S8. The various interactions for the most promising test compounds into the KRAS binding pockets.

KRAS							
Comp. No	Hydrogen Bonds		Hydrophobic Interactions	Salt Bridges		Halogen Bonds	
	Length Å	AA	AA	Length Å	AA	Length Å	AA
6a	2.41	CYS12B	GLU63B, ARG68B, TYR96B	5.32	LYS16B	3.71	GLU63B
6d	3.01	ARG68B	VAL9B, ALA11B, TYR96B, ILE100B	4.57	HIS95B	-	
6e	-		VAL9B, LYS16B, GLU62B, GLU63B, ARG68B	4.04	ARG68B	-	
6g	-		ALA11B, GLU62B, ARG68B	4.55	LYS16B	-	

AA: amino acids
